# Predictive classifier models built from natural products with antimalarial bioactivity using machine learning approach

**DOI:** 10.1371/journal.pone.0204644

**Published:** 2018-09-28

**Authors:** Samuel Egieyeh, James Syce, Sarel F. Malan, Alan Christoffels

**Affiliations:** 1 South African Medical Research Council Bioinformatics Unit, South African National Bioinformatics Institute, University of the Western Cape, Cape Town, South Africa; 2 School of Pharmacy, University of the Western Cape, Cape Town, South Africa; International Centre for Genetic Engineering and Biotechnology, INDIA

## Abstract

In view of the vast number of natural products with potential antiplasmodial bioactivity and cost of conducting antiplasmodial bioactivity assays, it may be judicious to learn from previous antiplasmodial bioassays and predict bioactivity of these natural products before experimental bioassays. This study set out to harness antimalarial bioactivity data of natural products to build accurate predictive models, utilizing classical machine learning approaches, which can find potential antimalarial hits from new sets of natural products. Classical machine learning approaches were used to build four classifier models (Naïve Bayesian, Voted Perceptron, Random Forest and Sequence Minimization Optimization of Support Vector Machines) from bioactivity data of natural products with *in-vitro* antiplasmodial activity (NAA) using a combination of the molecular descriptors and two-dimensional molecular fingerprints of the compounds. Models were evaluated with an independent test dataset. Possible chemical features associated with reported antimalarial activities of the compounds were also extracted. From the results, Random Forest (accuracy 82.81%, Kappa statistics 0.65 and Area under Receiver Operating Characteristics curve 0.91) and Sequential Minimization Optimization (accuracy 85.93%, Kappa statistics 0.72 and Area under Receiver Operating Characteristics curve 0.86) showed good predictive performance for the NAA dataset. The amine chemical group (specifically alkyl amines and basic nitrogen) was confirmed to be essential for antimalarial activity in active NAA dataset. This study built and evaluated classifier models that were used to predict the antiplasmodial bioactivity class (active or inactive) of a set of natural products from interBioScreen chemical library.

## Introduction

The devastating effect of malaria is evidenced by 584,000 deaths of which 78 percent were children under five years of age in 2013 [1] and thousands of person-hours lost to morbidity [2,3]. Majority of deaths due to malaria are caused by *Plasmodium falciparum*, the most virulent amongst the species that cause the disease [4–6]. The growing resistance and failure of existing first-line antimalarial drugs have exacerbated the situation leading to an exigent need to develop novel antimalarial drug candidates [7–9]. Judging by the immense contribution of nature to existing antimalarial drugs [10–13] and the likelihood to encounter novel chemotypes in natural products, *in-vitro* malarial screen data of natural products may be the appropriate starting point for the discovery of new antimalarial drugs.

Recently a number of publications have reported the *in-vitro* antiplasmodial activities of natural products from plants [10–13] and marine life forms [14,15]. In addition, datasets of *in-vitro* antiplasmodial bioassays of natural products and synthetic compounds have been made available in public domain [16–18]. The availability of such data for malaria drug discovery has motivated us to create predictive models based on molecular properties using machine-learning approaches.

Machine Learning, an aspect of artificial intelligence, is the practice of using algorithms to analyze input data (training data), learn from it, and then make a prediction on another set of related or unrelated data. Machine learning approaches may be supervised or unsupervised if the algorithms learned from labelled or unlabeled data [19]. Unsupervised statistical learning allows learning of relationships and structure of input data. Supervised machine learning involves building a model for predicting an output based on one or more sets of input data.

It has been shown that machine learning approaches could accurately predict the activities in assorted sets of compounds with activities as diverse as anti-tubercular [20], antimalarial [21] and RNA-binders [22]. To our knowledge, there has not been any bioactivity predictive model specifically for natural products with antiplasmodial or antimalarial activities. Increasing number of natural products, mostly from ethnomedicine in malaria-endemic regions, show good *in-vitro* and/or *in-vivo* antiplasmodial activities [23–26]. The antiplasmodial bioactivity data for these natural products present a dais to build models that may be used to screen other natural products and predict their potential antiplasmodial activities.

This present study focused on the development of machine learning classification models for natural products with varying *in-vitro* antiplasmodial activities (NAA). Four classification models were built from the bioactivity class (Active or Inactive) and a combination of molecular descriptors (MD) and molecular fingerprints (MF) of the NAA dataset. The performances of the classification models were assessed with standard model evaluation parameters (including accuracy and area under the Receiver Operating Characteristic (ROC) curve). We also analyzed the chemical structures of the datasets to find molecular fragments or chemical features enriched within the active and inactive compounds. Finally, we showed that the machine learning models built in this study might be used to screen large natural compound libraries *in-silico* and identify potential antiplasmodial compounds. This may limit the need for *in-vitro* screening and drastically reduce the expense of finding hits from natural products for antimalarial drug discovery.

## Materials and methods

**Fig 1:**
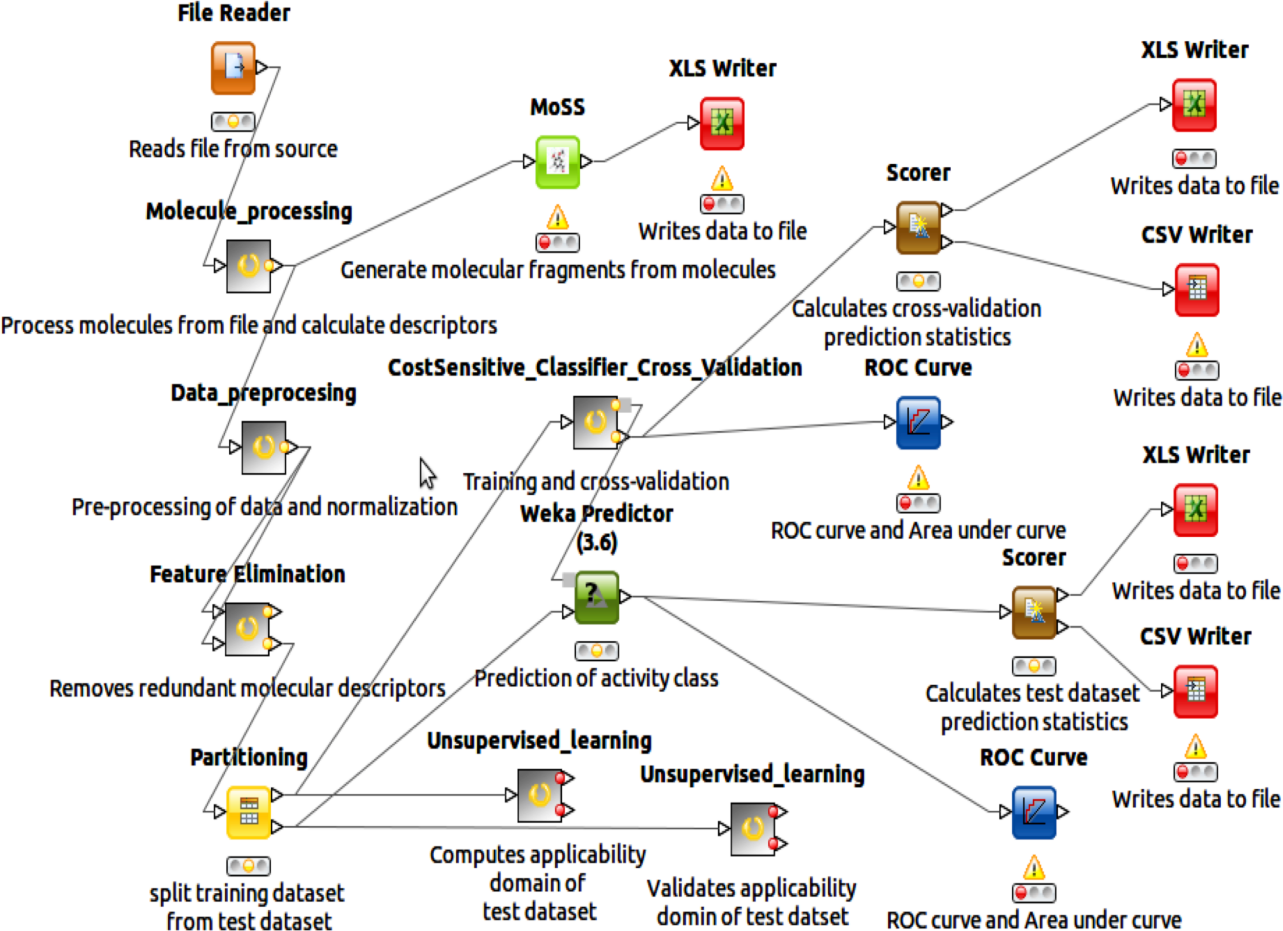
KNIME workflow. Screen-shot of the KNIME workflow used to build the classifier machine-learning models.

An original Konstanz Information Miner (KNIME) workflow [27,28] was set up (Fig 1) and used for the machine learning from our set of natural products with *in-vitro* antiplasmodial activities (NAA) in order to predict the activity class (active or inactive) of NAA.

### Data

The dataset used in this study consist of natural products that have been tested for *in-vitro* antiplasmodial activities (NAA) compiled in-house from literature, PhD and Masters Theses and public chemical databases. The chemical structures of the compounds in NAA were either downloaded in the SMILES format from public chemical databases (ChEMBL or PubChem) or drawn using Chemtool version 1.6.13 (http://ruby.chemie.uni-freiburg.de/~martin/chemtool) running on a Linux platform. The dataset (NAA) was subdivided into two groups based on their *in-vitro* antiplasmodial activities (IC_50_): Active (A) (IC_50_: < 10 μM) and Inactive (A) (IC_50_: ≥10 μM). A total of 1155 NAA compounds were used in this study, with 70% classified as active and 30% as inactive (S1 Table).

### Machine learning algorithms

Four classifier algorithms were used to learn from the dataset: Naïve Bayesian classifier [29,30], Sequential Minimization Optimization (SMO) classifier, a strategy for solving the quadratic problems during training with Support Vector Machine (SVM) [31,32], Random Forest (RF) classifier [33,34] and Voted perceptron (VP) classifier [35,36]. The specific classifiers were chosen in an attempt to represent four major types of classifiers models: Naïve Bayes represents the Bayes classifiers; Random Forest represents the tree-based classifiers; SMO represents the function-based classifier; and the Voted Perceptron represents the neural network classifiers. The classifier algorithms were executed with Waikato Environment for Knowledge Analysis (Weka 3.6) nodes [37] in Konstanz Information Miner (KNIME) [38].

### Dataset pre-processing and calculation of molecular descriptors and molecular fingerprints

The “RDKit Descriptor Calculation” and “RDKit Fingerprint” nodes [39] were used to calculate the molecular descriptors and molecular fingerprint. The “Data_preprocessing” node was then used to normalize the molecular descriptors using a minimum-maximum normalization model. The bit vector representing the molecular fingerprint was expanded into individual columns for each compound.

### Selection of descriptors or features

The objective of features selection is three-fold: improving the prediction performance of the predictive model, providing faster and more economical predictive models, and providing a better understanding of the underlying process that generated the data [40]. The “Feature Elimination” (FE) meta-node in KNIME was used to select descriptors that are beneficial to build efficient classifier models.

### Training of classifier models

The purpose of the classification algorithm was to build a classifier model that assigns a class (e.g. active/inactive) to molecules defined by a set of attributes (e.g. molecular descriptors). A metanode in the KNIME workflow (Fig 1) was designed to build the various classifier models that were earlier mentioned (i.e. Naïve Bayesian classifier, Sequential Minimization Optimization (SMO) classifier, Random Forest (RF) classifier and Voted perceptron (VP) classifier). The Partitioning node was used to split the data coming from the Feature Elimination meta-node into 80% training cum validation set and 20% independent test set by stratified sampling. The former was then piped into the “CostSensitive_Classifier_Cross_Validation” meta-node while the later was passed to the Weka Predictor (3.6) node. The Weka “Cost Sensitive Classifier” was used to build the classifier models and the Weka Predictor generated predictions from the test data. Regarding the features used to build the model, the molecular descriptors and molecular fingerprints were initially used separately to train the models. In an attempt to improve the accuracy of the model, we combined the molecular descriptors and the molecular fingerprints for each compound and used that combined feature to train the models.

### Class Imbalance and cost-sensitive classification

The imbalance bioactivity class (70% active and 30% inactive) was recognized as a major limitation to building a reliable model. Most bioassay datasets are imbalanced where one class is overly represented as observed in our datasets (approximately 70% active class (A) and 30% inactive class (N)). Prior to building the classifier model, the “SMOTE (Synthetic Minority Over-sampling Technique)” node within the KNIME was used to balance the bioactivity classes [38]. This node oversamples the input NAA dataset to enrich the inactive instances in the training dataset.

In addition, cost-sensitivity, which does not assume equality of the *costs* caused by different kinds of errors, was applied to the classifier algorithms used in this study. The Weka “meta-CostSensitiveClassifier” node in KNIME [38] was used to build the classifier models from NAA dataset. The Weka “meta-CostSensitiveClassifier” makes its base classifier cost sensitive and provide it with the capability to predict a class that leads to the lowest expected cost [37,41]. For our datasets that have two class representations (i.e. active (A)/inactive (N)), cost sensitivity was introduced by using a ‘2 ×2’ dimension cost matrix (Table 1).

**Table 1:** Cost matrix used by weka “meta-cost sensitive classifier”.

TP (0.0)	FN (2.0)
FP (1.0)	TN (0.0)

The cost values for each possible classification are in brackets. *True Positives (TP)*, *False Positives (FP)*, *True Negatives (TN) and False Negatives (FN)*

The four sections of a cost matrix can be read as True Positives (TP)—actives classified as actives; False Positives (FP)–inactives classified as actives; True Negatives (TN)—inactives classified as inactives; False Negatives (FN)—actives classified as inactive. The Weka “meta-CostSensitiveClassifier” enforces a penalty or weight on the base classifier for generating false positives (FP) or false negatives (FN) during learning. By default, the weight on the cost matrix is set to one for FP and FN. However, it has been reported that during the development of the classifier models the cost of misclassification may not always be the same [42]. In bioactivity prediction, the cost of FN (misclassification of an active compound as inactive) may be greater than the cost of FP (misclassification of an inactive compound as active) [20]. That is, the cost of missing a potential active compound is greater than the cost of predicting an inactive compound as active [20]. Therefore, the weight or penalty for FN was set to two (Table 1) to minimize the chance of FN misclassification. This cost matrix was used to build the NB, VP, SMO and RF classifier models.

### Classifier model performance evaluators

#### Accuracy statistics and receiver operating characteristic

The performances of the classifier models were assessed by accuracy statistics and Receiver Operating Characteristic graph after a 10-fold cross validation of a training set and prediction of the bioactivity class of an independent test set. In the KNIME workflow (Fig 1), the Scorer node and the ROC node were attached to the output from the Weka predictor nodes (from the cross-validation and the independent test data prediction). The outputs from the Scorer node include a confusion matrix and evaluation statistics (including accuracy of the prediction, Kappa statistic and mean absolute error). Accuracy indicates the proximity of measurement of results to the true value. This can be mathematically expressed as:

$$Accuracy=\frac{TP+TN}{TP+FP+TN+FN}*100$$ (1)

Where TP is True Positives; FP is False Positives; TN is True Negatives and FN is False Negatives.

The outputs from the ROC node encompass the Receiver Operating Characteristic (ROC) curve, which is a graphical plot of True Positive Rates (TPR) vs. False Positive Rates (FPR) for a binary classification system. The Area under Curve (AUC) value was also computed from the ROC curve and in our case, it denotes the probability that a classifier will rank a randomly chosen active compound higher than a randomly chosen inactive compound.

### Applicability domain (AD)

Generally, machine learning models methods are more likely to show good predictive performance for compounds that share similar properties to compounds in the training set. Thus, it is necessary to define the “applicability domain” (i.e. the boundary defined by the chemical space in the training set) of the models and to check if new test compounds fall within such domain [43]. One of the simplest and commonly applied methods used to define AD is based on range-based definition with a preliminary Principal Components **(**PC) rotation [44]. In the present study, we defined the AD of the models using the training data and evaluated the extent to which the independent test data fit into the AD. This will be helpful to explain the accuracy of prediction from models and assess whether a new compound is inside or outside the AD of the models.

Principal Component Analysis **(**PCA) of the molecular fingerprints of the compounds in the training/validation data was done with Unsupervised learning metanode in the machine learning KNIME workflow (Fig 1). The PCA was carried out for the training data from the Partition node in the KNIME workflow before cross- validation (Fig 1). PCA of the independent test dataset was also performed in order to validate if the compounds within the test dataset fall within the chemical space or applicability domain (AD) of the compounds in the training dataset.

### Enriched molecular fragments in the NAA datasets

The molecular fragments or substructures (or chemical features) enriched within the active and inactive compounds in the NAA dataset was searched with the Molecular Substructure (MoSS) node in KNIME (Fig 1) [27,28]. Minimum and maximum fragment sizes were set to 1 and 100 respectively. Pure carbon fragments were ignored and the ring mining option was enabled (set at 3 to 8 to avoid finding fragments with partial rings). The algorithm used is the Christian Borgelt’s MoSS implementation [45].

## Results and discussion

In the present study, we have trained and evaluated four antiplasmodial activity classification models based on a combination of molecular descriptors and molecular fingerprints of natural products with antiplasmodial activity (NAA).

### Molecular descriptors and molecular fingerprints

A total of 117 molecular descriptors were generated with RDKit Descriptors Calculation node in KNIME [46,47] for the compounds in the NAA dataset. The resultant data was then pre-processed, as described under the method section, before passing on to the “Feature Elimination” meta-node [48] to remove redundant molecular descriptors. Approximately 35% of the molecular descriptors were removed from the NAA dataset. The molecular fingerprints of the compounds in NAA datasets were also generated with RDKit Fingerprint node in KNIME [36,37]. The remaining 76 molecular descriptors were combined with the molecular fingerprints and used to train the classification models.

### Training of classifier models and cross-validation

Four classifier models were trained with natural products with in-vitro antiplasmodial activities (NAA) (using Weka version 3.6 node in KNIME): Voted Perceptron (VP), Naïve Bayesian (NB), Random Forest (RF) and Sequential Minimization Optimization (SMO). Running on a Dell Vostro laptop (Intel Core i3-2328M CPU @ 2.20 GHz x 4), SMO was the slowest in terms of program runtime to build one model (2.88 seconds); the NB was the fastest (0.12 seconds) followed by VP (1.05 seconds) and RF (1.31 seconds). A total of 1147 NAA (labelled as 70% active and 30% inactive) was used in this study. This was divided into 917 NAA for training cum 10-fold cross-validation of the classifier model and 230 NAA as the independent test dataset. Misclassification cost was set to two for false negatives (FN).

**Fig 2:**
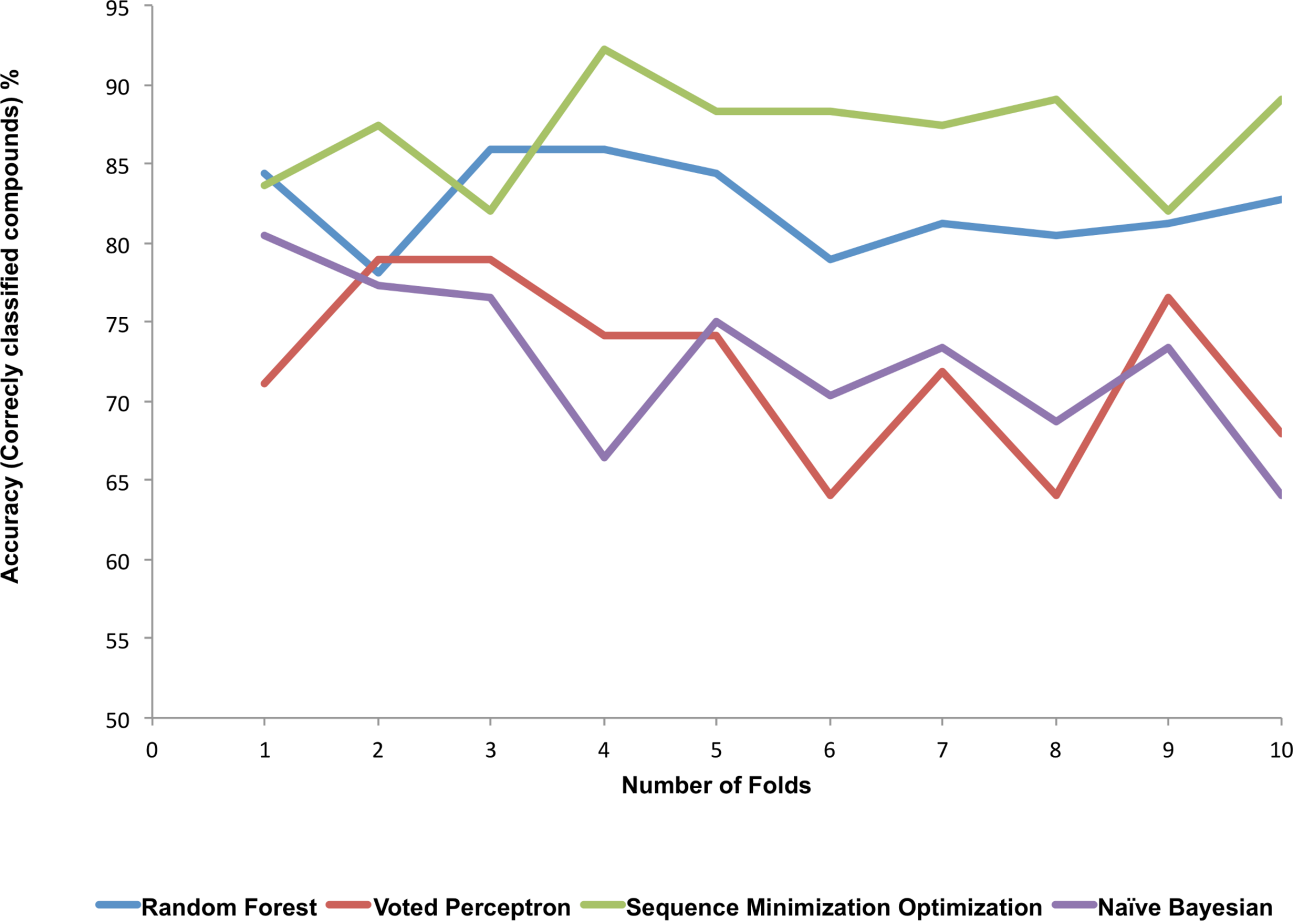
The graph shows values of the accuracy (percentage of correctly classified compounds) of the four classifier models over the 10 folds cross-validation. The sequence minimization optimization (SMO) and Random Forest (RF) classifier models showed greater predictive accuracy than the Naïve Bayesian (NB) and Voted Perceptron (VP) classifier models.

The values of the accuracy (percentage of correctly classified compounds) of the classifier models over the 10 fold cross-validation are presented in Fig 2. Accuracy may be defined, specifically for this study, as the proportion of compounds that were correctly classified as active and inactive (i.e. the number of compounds correctly classified divided by the total number of compounds classified multiply by 100). From the results (Fig 2), SMO and RF classifier models showed greater predictive accuracies than the NB and VP classifier models for the NAA dataset. Moreover, the values of the predictive accuracy were fairly consistent over the 10 fold cross-validation for SMO and RF judging by the slope of approximately 0.2 for the data points on the graph. This is an indication of the consistency of the predictive abilities of these classifier models.

The goal here is to see the performance of the trained classifier models to predict the activity class of 10 randomly selected test datasets. From the result (Fig 2), we may conclude that SMO and RF classifier models showed good and fairly consistent predictive performance of the 10 randomly selected test datasets. However, we used all classifier models generated to predict the bioactivity class of the independent NAA test dataset that was not included in the training and cross-validation dataset.

### Prediction of bioactivity class of an independent NAA test dataset

The classifier models (Sequential Minimization Optimization (SMO), Random Forest (RF), Voted Perceptron (VP) and Naïve Bayesian (NB)), previously trained and cross-validated as described earlier, were used to predict the bioactivity class of an independent test dataset of natural products with *in-vitro* antiplasmodial activities (NAA). The performances of the classifier models were evaluated using accuracy, Kappa statistics and Receiver Operating Characteristic curve.

#### Accuracy

From the results (Table 2), the Sequence Minimization Optimization (SMO) of Support Vector Machine model showed the highest accuracy (85.94%) followed by the Random Forest (RF), 82.81%. Naïve Bayes (NB) and Voted perceptron (VP) models displayed accuracy just above 70% (73.05% and 71.48% respectively). When we compared the results from the fusion model to the individual models (RF and SMO), we see that the accuracy did not change.

**Table 2:** Evaluation parameters from the prediction of bioactivity class of an independent NAA test dataset by the four classifier models used in this study.

Classifier Models	Accuracy (%)	Kappa Statistics	Area Under Curve (ROC)
Random Forest (RF)	82.81	0.65	0.91
Voted Perceptron (VP)	71.48	0.42	0.72
Sequence Minimization Optimization (SMO) of Support Vector Machine	85.94	0.72	0.86
Naïve Bayesian (NB)	73.05	0.45	0.74
Fused Model (RF and SMO)	82.03	0.68	0.92

**Comments:** RF: Random forest of 10 trees, each constructed while considering 11 random features. Out of bag error: 0.1797. SMO: The polynomial kernel. Fused Model (RF and SMO): The predictions from RF and SMO were combined (mean) using the “Prediction Fusion” node in KNIME [38].

The objective here was to identify the classifier model trained with both molecular descriptors and molecular fingerprints that best predict the bioactivity class of the independent NAA test dataset. From the results, we may conclude that SMO of SVM and RF models are the most suitable classifier models for NAA. Though accuracy provided an overall estimation of the performance of the classifier models, one limitation to the use of accuracy as a metric for assessing predictive performance of classifier models is “accuracy paradox” (i.e. a classifier model with a given level of accuracy may have greater predictive power than models with high accuracy). Therefore less biased metrics like the Kappa statistics and area under Receiver Operating Curve (ROC) were used as a more objective evaluator of the predictive powers of the classifier models.

#### Kappa statistics

The results (Table 2) showed that the kappa statistics of SMO and RF classifier models (0.72 and 0.65 respectively), like their accuracy values, were higher than that of the other classifier models in this study. The results also showed that the fusion model showed a very slight increase in its Kappa statistics when compared to the individual models (RF and SMO). The value of the Kappa statistics is often used as a measure of consistency or agreement between the *“ground truth”* (the actual class of each compound to be classified) and classifier models’ classification (the class assigned to the compounds by the classifier model). It accounts for the chance of random classification of compounds into the two bioactive classes (Active and Inactive). Kappa statistic values of 1 suggest a perfect agreement between the *“ground truth”* and classifier models’ classification. Judging by the kappa statistics of SMO and RF models (Table 2), which are closer to 1 than that of NB and VP models, we concluded that SMO and RF classifier models showed the best predictive power as similarly observed with the use of accuracy as the evaluator of the classifier models.

#### Receiver operating characteristic plot (ROC)

**Fig 3:**
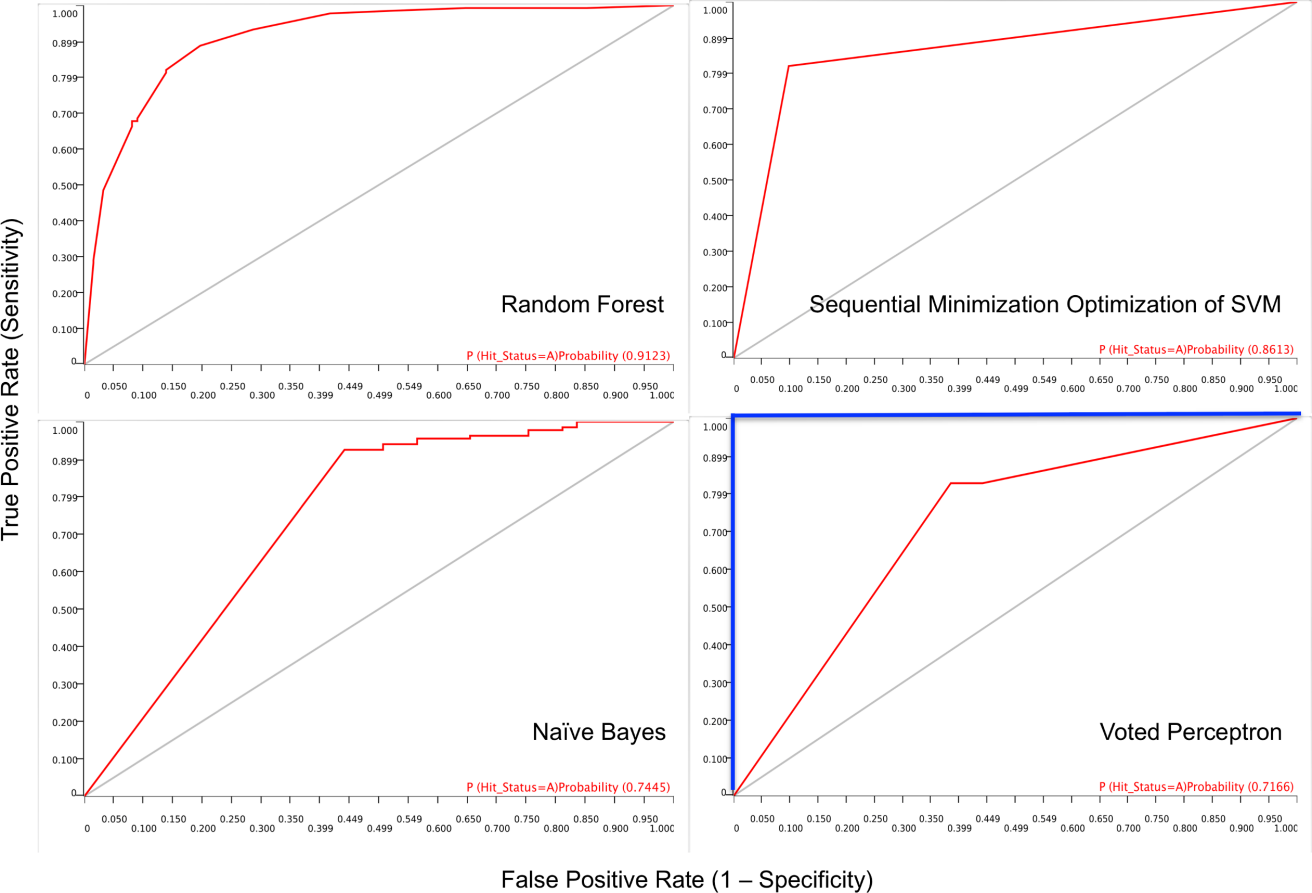
The Receiver operating characteristics (ROC) curve for the four classifier models. The diagonal grey line represents classifier models that randomly assign compounds to bioactivity class (and will have an area under the curve (AUC) of 0.5). The blue line shown in the ROC curve of Voted perceptron (will have an AUC of 1.0) represents classifier models that perfectly predict bioactivity class of compounds. The red line is the ROC curve from the predictions by the four classifier models. The area under the ROC curve (AUC), a measure of bioactivity class discriminatory power of a classifier model, is shown on each ROC curve.

Fig 3 shows the Receiver Operating Characteristic (ROC) curve of the classifier models trained and evaluated. Receiver Operating Characteristic (ROC) curve is a graphical plot that shows the performance of a binary classifier model as its discrimination threshold is varied.

ROC is a plot of the true positive rate (Sensitivity) against the false positive rate (1 –Specificity) at various threshold settings. The diagonal grey line represents classifier models that randomly assign compounds to bioactivity class. The blue line shown in the ROC plot of Voted perceptron represents classifier models that perfectly predict bioactivity class of compounds. The red line is the ROC curve from the predictions by the four classifier models in this study. In contrast to NB and VP classifier models, SMO, RF and the fusion classifier models showed ROC curves that were initially very close to the true positive rate axis (i.e. minimizing false positive rate (maximizing specificity) and maximizing true positive rates (maximizing sensitivity)). An optimum prediction aims to maximize sensitivity and specificity. However in all the models, as the threshold changes the false positive rate increases (i.e. the specificity decreases) and the true positive rate (i.e. sensitivity) approaches its maximum value. Hence low specificity values may lead to high incidence of false positives (i.e. detecting inactive compounds as active). It is therefore expedient to choose the threshold that will have good specificity and thus avoid investing resources to synthesize and conduct bioassays for compounds that may not be active and fail along the drug development pipeline [49–52].

#### Area under the receiver operating characteristic curve (AUC)

The values of the area under the ROC curve (AUC) are shown in Table 2. The RF had the highest value of AUC of 0.91 followed by SMO with an AUC value of 0.86. The VP and NB classifier models showed AUC values of 0.72 and 0.74 respectively. The fusion model showed AUC that is higher than AUC of SMO but similar to the AUC seen for RF. The ROC The area under the ROC curve (AUC) is a measure of how well a model can discriminate between two classes in a dataset (e.g. active and inactive compounds) [53]. In this study, AUC depicts the probability that the active class predicted by the classifier models for a randomly selected compound will exceed that of a randomly selected non-active class [54]. Where the prediction of the bioactivity class of compounds is purely random, the AUC will be equal to 0.5 (i.e. the ROC curve will coincide with the diagonal line). When the prediction results in perfect separation of the bioactivity class of the compounds, i.e. where there is no overlapping of the distributions of the bioactivity classes, the area under the ROC curve will be one.

The values of the AUC for the four classifier models indicate that the discriminatory or predictive power (separation of the bioactivity class of the compounds) of the models range from fair (0.7–0.8) to excellent (> 0.9). The discriminating powers of the classifier models, judging by the AUC, were thus: RF, SMO, NB and VP in decreasing order of discriminating power to predict a bioactive class of the compounds in the NAA dataset. Overall, the nature of the ROC plot and the higher AUC values of RF and SMO suggest their suitability as good classifier models for the NAA dataset used in this study.

### Applicability domain (AD) of the classifier models

Applicability Domain (AD) of the classifier models refers to the chemical space, defined by the training set, within which a test compound should be in order for its bioactivity class to be reliably predicted. In this present study, the AD of the models was defined with the training and cross-validation dataset and its validity evaluated on the independent test dataset. Principal Component Analysis (PCA) was used to define the AD of the models and to map the test dataset (active and inactive compounds) in their respective chemical spaces.

**Fig 4:**
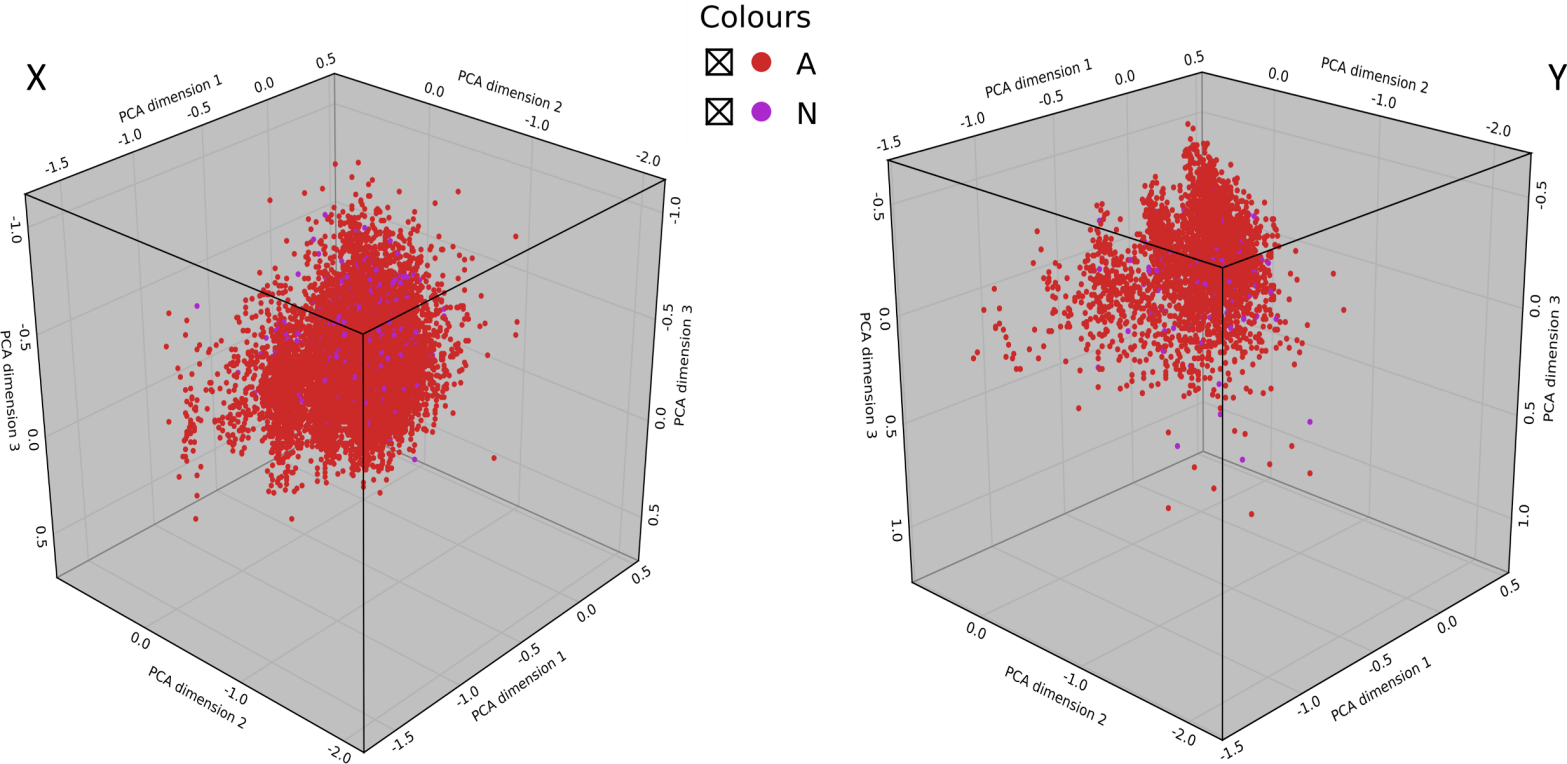
A visualization of the applicability domain (chemical space) of the of classifier models built in this study. Active compounds (red dots) and inactive compounds (purple dots) are represented using the first three Principal Components. Panel X depicts the range of Principal Components of compounds in the training set that define the applicability domain (AD). Panel Y shows that almost all compounds in the test set fell within the AD of the defined by the training set. Therefore, classifier models generated in this study can reliably predict the bioactivity class of new compounds that fall within this AD. *NAA*: *natural products with in-vitro antiplasmodial activity*.

Fig 4 is the visualization of the first three principal components of the compounds in the training and cross-validation dataset (Fig 4 (X)) and compounds in the independent test dataset (Fig 4 (Y)) for the NAA dataset. From Fig 4(Y) and 4(X), we observed that almost all compounds in the test dataset fell within the chemical space or AD of the training dataset used to build the classifier models.

The results also revealed no clear boundary between the active and inactive compounds in the training dataset and the independent test dataset for NAA. This implies some level of chemical structural similarity amid the active and inactive compounds in the datasets, which may pose a restriction on the discriminatory ability of the models. Overall, this analysis enabled the identification of the AD for the models built in this study. Therefore the models may reliably predict new compounds that fall within this AD.

### Enriched molecular fragments in the NAA datasets

We sought to understand the molecular substructures (or chemical features) associated with antiplasmodial activity and inactivity of compounds in the NAA (natural products with *in-vitro* antiplasmodial activities) dataset. To this end, we used the Molecular Substructure (MoSS) node in KNIME to search for most common molecular substructures in the active and inactive compounds in the NAA dataset.

**Fig 5:**
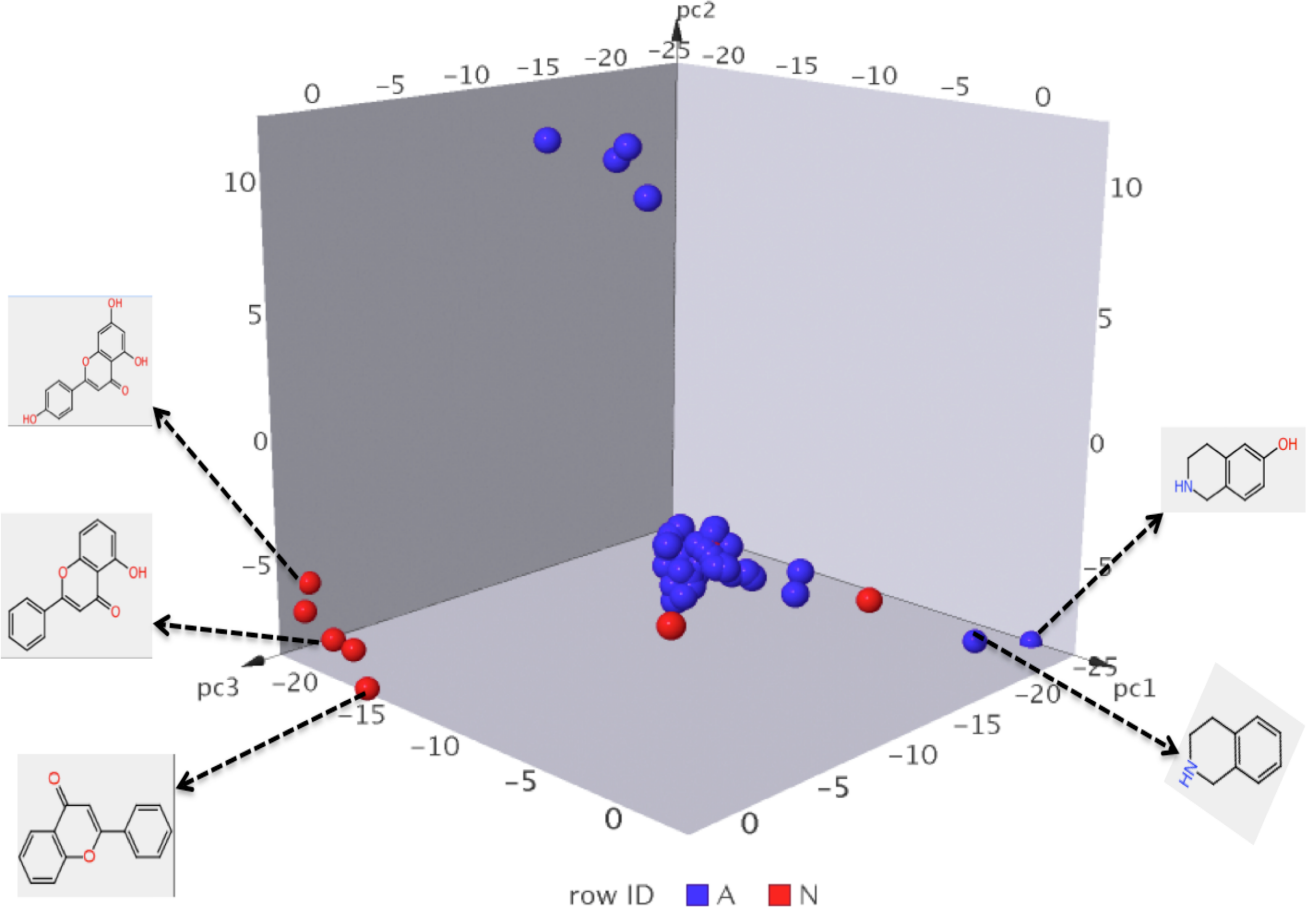
Chemical features from active and inactive compounds from NAA dataset. The blue markers represent most common substructures from active compounds (IC50 ≤ 10 μM) while the red markers represents most common substructures from inactive compounds (IC50 > 10 μM). The most common substructures were projected in a three-dimension (3D) space based on molecular similarity. Some of the most common substructures that are peculiar to the active and inactive compounds are highlighted. This may guide rational selection and design of active antiplasmodial compounds. *NAA*: *natural products with in-vitro antiplasmodial activity*.

A total of 52 most common molecular substructures from active compounds (717 compounds) and 48 most common molecular substructures from inactive compounds (323) were identified. The molecular similarities amongst the substructures from the active and inactive compounds were estimated and projected in a three dimensional (3D) space (Fig 5).

From these results, most of the substructures from the active and inactive compounds overlapped in the 3D space indicating their high molecular similarity. However, some of the substructures from the active and inactive compounds occupy a distinct region of the 3D space (Fig 5). These include hydroxyisoquinoline and isoquinoline substructures from active compounds and hydroxyflavone from inactive compounds. These substructures may be determinants of antiplasmodial activities and may guide rational selection and design of active antiplasmodial compounds. A closer at difference in the functional groups between the active NAA dataset (A) and inactive NAA dataset (N) revealed the following: Akylamine (29% in A, 13% in N); Aromatic amine (1.5% in A, 3% in N); Basic nitrogen (36% in A, 14% in N); Acidic oxygen (4% in A, 9% in N). In all, the amine chemical group (specifically alkyl amines and basic nitrogen) was confirmed to be essential for antimalarial activity in active NAA dataset.

### Benefits of models from machine learning (*in-silico* compound screening)

To illustrate the benefit of the machine learning and the resultant classifier models, the Sequential Minimization Optimisation (SMO) and Random Forest classifier models, adjudged the top classifier models in this study, were used to screen 450 natural compounds of a private natural product chemical library from InterBioScreen (http://www.ibscreen.com). The results (S2 Table) showed that the SMO classifier model predicted that 39% of the compounds will possess active antiplasmodial activities while Random Forest predicted a higher proportion of the natural product chemical library as active (87%). Although there was a significant difference in the proportion of compounds predicted as active by the classifier models the output from the RF classifier model may be less reliable due to the tendency for RF models to overfitting data [33,34]. The two classifier models showed consistent antiplasmodial bioactivity class prediction for 54% of the compounds in the natural product chemical library.

The natural compounds predicted as active, which are readily available from InterBioScreen and other chemical libraries, may be prioritized and readily purchased for *in-vitro* antiplasmodial screening. Overall, these results attest to the importance of bioactivity predictive models built with machine learning algorithms that are capable of effective and efficient learning from existing bioactivity data and predicting biological activities *in-silico* to modern drug discovery.

## Conclusions

In this study, we used machine learning as a method to build various antimalarial predictive models that can predict the bioactivity class of natural products. The classifier models that were most suitable for the dataset (natural products with *in-vitro* antiplasmodial activities) were identified. These models were used, *in-silico*, to annotate potential antimalarial compounds in a large natural product library. Such compounds may be prioritized for the more expensive *in-vitro* bioactivity screening. In addition, we generated a pool of chemical features that were present within active and inactive natural products with *in-vitro* antiplasmodial activities (NAA) used in this study. Such chemical features from active NAA in conjunction with the molecular scaffolds that may be identified from the active NAA could be valuable in designing antimalarial specific virtual compound library.

The knowledge of the classifier models that provide the most accurate prediction of the desired bioactivity for a particular class of compounds will enable medicinal chemist to pre-screen compounds prior to the expensive step of synthesis and *in-vitro* assay. Accurate prediction of bioactivity class of compounds will improve decision-making processes in antimalarial drug design and development to achieve better and cost-effective outcomes (i.e. drug candidate for malaria). Overall, knowledge provided by this study could contribute significantly to and accelerate the on-going efforts for antimalarial drug discovery, especially from natural products.

## Supporting information

S1 Table Natural products that have been tested for *in-vitro* antiplasmodial activities (NAA) compiled in-house from literature, PhD and Masters Theses and public chemical databases. (PDF)

S2 Table The antiplasmodial bioactivity class predictions of 450 natural compounds from a private natural product chemical library from InterBioScreen (http://www.ibscreen.com) by sequential minimization optimisation (SMO) and random forest classifier models. (PDF)
