# Supplementary material for: Predictive classifier models built from natural products with antimalarial bioactivity using machine learning approach
Source: PLoS One. 2018 Sep 28;13(9):e0204644. doi: 10.1371/journal.pone.0204644 (PMC6161899; doi:10.1371/journal.pone.0204644)
Supplement: S1 Table — (PDF) [file pone.0204644.s001.pdf]

S1 Table. Natural products that have been tested for *in-vitro* antiplasmodial activities (NAA) compiled in-house from literature, PhD and Masters Theses and public chemical databases.

ID	Hit_Status	pIC50	Smiles
N78	A	6.15490196	C1CC(C(CC(CN2C=Nc3ccccc3C2=O)=O)NC1)O
N79	A	5.920818754	[C-]#[N+]C1C2C(CCC1(C)O)C(C)(CCC2C1(C)CCC(C(C)(C)O1)[Cl])[N+]#[C-]
N80	A	5.468521083	C1CC2C(CC(CN3C=Nc4ccccc4C3=O)(O)O2)NC1
N81	A	4	CCC(C)C(C(CC(N1CCCC1C(C(C)C(NC(Cc1ccccc1)c1nccs1)=O)OC)=O)OC)N(C)C(C(C(C)C)NC(C(C(C)C)N(C)C)=O)=O
N82	A	2.836148955	CC(C)C(C)=CC(=O)OC1C2C34COC2(C(C(C3C2(C)CC(C(=C(C)C2CC4OC1=O)O)=O)O)O)C(=O)OC
N83	A	2.738805434	CC1C(C(C)(CC(O1)OC1CC(C(c2c1c(c1cc(C)c3C4C5C(C6(CO6)C(C(OC)OC)(O4)O5)(OC4CC(C(C(C)O4)(C(C)=O)O)O)Oc3c1c2O)OC)=O)O)O)OC(C)=O
N84	A	2.717469423	CC1C(C(C)(CC(O1)OC1CC(C(c2c1c(c1cc(C)c3C4C5C(C6(CO6)C(C(OC)OC)(O4)O5)(OC4CC(C(C(C)O4)(C(C)=O)O)O)Oc3c1c2O)OC)=O)O)O)O
N85	A	2.698970004	CC(c1cc2C(c3ccccc3C(c2o1)=O)=O)=O
N86	A	2.698970004	CC1C(C(C(C(O1)OC1=C(c2ccc(c(c2)O)O)Oc2cc(cc(c2C1=O)O)O)O)O)O
N87	A	2.698970004	CC1C=C2C(=CC(=O)O2)C2(C)C1CC1C34COC(C4C(C(=O)O1)OC(C)=O)(C(C(C23)O)O)C(=O)OC
N88	A	2.638272164	C([C@@H]1[C@H]([C@@H]([C@H]([C@H](OC2=C(c3ccc(c(c3)O)O)Oc3cc(cc(c3C2=O)O)O)O1)O)O)O)O
N89	A	2.537602002	C[C@@H]1[C@@H]([C@H]([C@H]([C@H](OC2=C(c3ccc(c(c3)O)O)Oc3cc(cc(c3C2=O)O)O)O1)O)O)O[C@H]1[C@@H]([C@H]([C@@H]([C@@H](CO)O1)O)O)O
N90	A	2.239320936	CC(C)=CC(=O)OC1C2C34COC2(C(C(C3C2(C)CC(C(=C(C)C2CC4OC1=O)O)=O)O)O)C(=O)OC
N91	A	2.2046155	CCC(C)C(=O)OC1C2C34COC2(C)C(C(C3C2(C)C(CC4OC1=O)C(C)=CC(C2O)O)O)O
N92	A	2.202789815	CCC(C)C(=O)OC1C2C34COC2(C)C(C(C3C2(C)C(CC4OC1=O)C(C)=CC(C2O)=O)O)O
N93	A	2.15490196	[H]C(=C1C(c2c(cc(cc2OC(C)=O)OC(C)=O)O1)=O)c1

			cc(c(c(c1)OC)OC(C)=O)OC
N94	A	2.098528764	CC1=CC(C(C2(C)C1CC1C34COC(C24)(C(C(=C)C3CC(=O)O1)O)O)O)=O
N95	A	2.052754154	CC(=CC(=O)OC1C2C34COC2(C(C(C3C2(C)CC(C(=C(C)C2CC4OC1=O)O)=O)O)O)C(=O)OC)C(C)(C)O
N96	A	1.974694135	CC(=C)C(CC=C(C)C)Cc1c(cc(c2C(CC(c3c(cccc3O)O)Oc12)=O)O)OC
N97	A	1.973712796	C1CCN2CCC3C(=CC(CCC=CC1)(C1C3(CC3C=CCCCN13)C2)O)c1c2c(ccn1)c1cccc(c1[nH]2)O
N98	A	1.966576245	CC(=C)C(CC=C(C)C)Cc1c(cc(c2C(CC(c3c(cccc3O)O)Oc12)=O)O)O
N99	A	1.962573502	CC(CC=CC(C(C(CC1C(c2cc(cc(c2C(=O)O1)O)OC)O)O)O)O)O
N100	A	1.93930216	CC12C(CC[Cl])C(NC1(C(C1CCCC=C1)O)C(=O)O2)=O
N101	A	1.903089987	C1CCN2CCC3C(=CC(CCC=CC1)(C1C3(CC3C=CCCCN13)C2)O)c1c2c(ccn1)c1ccccc1[nH]2
N102	A	1.899111024	CCC1CN2CCc3cc(c(cc3C2CC1CC1c2c(CCN1)c1cc(ccc1[nH]2)O)OC)OC
N103	A	1.895947674	CC1Cc2ccc(c3c(CO)cc(c4c(cccc34)OC)OC)c(c2C(C)N1)O
N104	A	1.853871964	[C-]#[N+]C1(C)CCC2C(C)CC3CC(C)C(C)(C4CCC1C2C34)[N+]#[C-]
N105	A	1.839231438	[C-]#[N+]C1(C)CCC2C(C)CC3CC(C)C(C)(C4CCC1C2C34)[N+]#[C-]
N1	A	1.839231438	[H][C@@]12CC[C@]3([H])C([C@H](C)C[C@]4([H])C[C@H](C)[C@]([H])(CCC1=O)[C@@]2([H])[C@]34[H])=O
N106	A	1.823908741	CC1=CC(C(C2(C)C1CC1C34COC(C)(C24)C(C(C3C(C(=O)O1)OC)O)O)O)=O
N107	A	1.769551079	C[C@@]1(C)[C@H]2CC[C@@]34CC(=C)[C@@H](CC3=O)C[C@H]4[C@]2(C)CC([C@H]1O)=O
N108	A	1.721246399	CC[C@@H](C)C(=O)O[C@H]1[C@H]2C(CC([C@@H](C)CCC[C@@]1(C)O)=O)C(=C)C(=O)O2
N109	A	1.721246399	CC[C@@H](C)C(=O)O[C@H]1[C@H]2C(CC([C@@H](C)CCC[C@@]1(C)O)=O)C(=C)C(=O)O2
N110	A	1.721246399	CC1C2C(Cc3c1cco3)C1(C)CCCC(C)(C)C1C(C2OC(C)=O)OC(C)=O
N111	A	1.721246399	CCC12CCCN3CCC4(C(CC1)N(C(C)=O)c1c4ccc(c1O)OC)C23
N112	A	1.698970004	[H][C@@]12C=C(C)CC(C)(C)[C@@]2([H])[C@]([H])(c2c(CCN(C)C)c3ccccc3[nH]2)n2c1c(CCN(C)C)c1cccc12

N114	A	1.698970004	C[C@H]1CC=C[C@@H]([C@H]([C@H](CC=Cc2cc(cc(c2C(=O)O1)O)OC)O)O)O
N113	A	1.698970004	CC(C)=CCc1c2c(C=CC(C)(C)O2)c(c2C(C3C(C=C(C)C)Oc4cc(c(cc4C=3Oc12)O)O)=O)O
N115	A	1.676728094	CC(C)CC(=O)OC1C2C34COC2(C(C(C3C2(C)CC(C(=C(C)C2CC4OC1=O)O)=O)O)O)C(=O)OC
N116	A	1.6402662	CC1C2CC3C45COC(C5C(C(=O)O3)OC(C)=O)(C(C(C4C2(C)CC(C=1O)=O)O)O)C(=O)OC
N117	A	1.628932138	CC1=CC2=CC(C=C3C2=C(C=C(C)O3)N1)=O
N118	A	1.602059991	C[C@H]1CC[C@H]([C@@H](C)[C@H]2CC[C@H]3[C@@H]4CC=C5C[C@H](CC[C@]5(C)[C@H]4CC[C@]23C)N)NC1
N119	A	1.568636236	CC1C2=CC=C3[C@@]4(C)CC[C@]5(CCC(=C)C[C@H]5[C@]4(C)CC[C@@]3(C)C2=CC(C=1O)=O)C(=O)OC
N120	A	1.522878745	[H]C(=C1C(c2c(cc(cc2O1)O)O)=O)c1cc([H])c(c(c1)OC)O
N121	A	1.450760382	CC1CCC2C(C)C(=O)OC3C24C1CCC(C)(O3)OO4
N122	A	1.437132011	CC1=CC(C(C2(C)C1CC1C34COC(C)(C(C(C23)O)O)C4(C(C(=O)O1)O)O)O)=O
N123	A	1.408935393	CC1(C)CCC2(CCC3(C)C(=CCC4C5(C)CCC(C(C)(C)C5CCC34C)O)C2C1)C(O)=O
N124	A	1.369815734	CCCCCCCc1cc(C=C2C(=CC(=C3C=CC=N3)N2)OC)[nH]c1C
N125	A	1.327902142	C1CCN2CCC3C(=CC(CCC=CC1)(C1C3(CC3C=CCCCN13)C2)O)c1c2c(ccn1)c1cccc(c1[nH]2)O
N126	A	1.326743909	CC1=CC(C(C2(C)C1CC1C34C2C(CO4)(C(C2(CO2)C3(C(C(=O)O1)O)O)O)O)O)=O
N127	A	1.301029996	CC(C)CC(=O)OC1C2C34COC2(C(C(C3C2(C)C(CC4OC1=O)C(C)=CC(C2O)=O)O)O)C(=O)OC
N128	A	1.301029996	CCC12CCCN3CCc4c5ccccc5n(C(C1)c1c(ccc5c1c1CC[N+]6(C)CC(=CC)C(CCO)CC6c1[nH]5)O)c4C23
N129	A	1.289276468	C[n+]1cc2cc(c(cc2c2ccc3cc(c(cc3c12)OC)O)OC)OC
N130	A	1.27572413	C1CN=C2C3=C(C(c4c2c1c[nH]4)=O)NC1CC32C=C(C(CC2S1)=O)[Br]
N131	A	1.27572413	CCCCC#CC(CO)C(=O)OCCc1ccc(c2cccs2)s1
N132	A	1.27572413	CCCCCC#CC(CO)C(=O)OCCc1ccc(c2cccs2)s1
N133	A	1.26609352	CC1C2=CC=C3C(C)(CCC4(C)C5CC(CCC5(C)CCC34C)CO)C2=CC(C=1O)=O
N134	A	1.259637311	CCC(C)C1C(N2CCCCC2C(NC(CCCCCC(CC)=O)C(NC(Cc2cn(c3ccccc23)OC)C(N1)=O)=O)=O)=O
N135	A	1.23759243	CCC1CN2CCc3cc(c(cc3C2CC1CC1c2cc(c(cc2CCN1

		4	)O)OC)OC)OC
N136	A	1.198942059	CC1Cc2c(C(C)N1)c(cc(c2c1ccc(c2c(cc(C)cc12)OC)O)O)O
N137	A	1.148741651	CC1=CC(C(C2(C)C1CC1C34COC(C)(C24)C(C(C3C(C(=O)O1)O)O)O)O)=O
N138	A	1.13667714	Cc1c2cnccc2c(C)c2c1c1ccccc1[nH]2
N139	A	1.102372909	C1C(C(c2cc(c(c(c2)O)O)O)Oc2cc(cc(c12)O)O)OC(c1cc(c(c(c1)O)O)O)=O
N140	A	1.096910013	CC(C)=C[C@H]1C[C@@](C)(C=Cc2c(CCNC)c3ccccc3[nH]2)n2c1c(CCN(C)C)c1ccccc12
N141	A	1.070581074	C1C2C3C=C(C4CC56C7CC8C9C5N(C(CC9OCC=C8CN47)=O)c4ccccc46)C(N4C3C3(CCN(CC2=CCO)C13)c1ccccc14)=O
N142	A	1.068416584	CN1CCc2cc(c3cc2C1Cc1ccc(cc1)Oc1cc(CC2c4c(CCN2C)cc(c(c4O3)O)OC)ccc1OC)OC
N143	A	1.064492734	CC1CCC(c2cc(C)ccc12)=O
N145	A	1.045757491	CC(C)=C[C@@H]1C[C@](C)(C=Cc2c(CCN(C)C)c3ccccc3[nH]2)n2c1c(CCN(C)C)c1ccccc12
N146	A	1.045757491	CC(C)=C[C@H]1C[C@](C)(C=Cc2c(CCN(C)C)c3ccccc3[nH]2)n2c1c(CCN(C)C)c1ccccc12
N147	A	1.045757491	CC1CC(C)(C)C2C(C=1)c1c(CCNC)c3ccccc3n1C2c1c(CCNC)c2ccccc2[nH]1
N148	A	1.026872146	CC1C2=CC=C3[C@@]4(C)CC[C@]5(CCC(=C)C[C@H]5[C@]4(C)CC[C@@]3(C)C2=CC(C=1O)=O)C=O
N149	A	1.022276395	CC[C@@H]1[C@](C)([C@@H]([C@@H](C)N(C)C[C@H](C)C[C@](C)([C@@H]([C@@H](C)[C@@H]([C@@H](C)C(=O)O1)O[C@H]1C[C@](C)([C@H]([C@H](C)O1)O)OC)O[C@H]1[C@@H]([C@H](C[C@@H](C)O1)N(C)Cc1ccc(cc1)c1cn(CCCCCCC(NO)=O)nn1)O)O)O)O
N150	A	1.009024851	CC1=CC(C(C2(C)C1CC1C34COC(C23)(C(C(=C)C4(C(C(=O)O1)O)O)O)O)O)=O
N151	A	1.008773924	[H]C12CCC3=CC4(CCC3(C2(C)CCCC1(C)CO)OO4)C(C)C
N152	A	1	CC=C1CN2C(CC34C2CC1C1=CCC(N(C13)c1ccccc14)=O)C1=CC2C3CC4C5(CCN4CC3=CCO)C2N(C1=O)c1ccccc15
N153	A	0.999559272	CC(C)=CC(=O)OC1C2C34COC2(C)C(C(C3C2(C)C(CC4OC1=O)C(C)=CC(C2O)=O)O)O
N154	A	0.974954995	CN1CCc2cc(c(c3c2C1Cc1ccc(cc1)Oc1cc(CC2c4cc(c(cc4CCN2)OC)O3)ccc1OC)O)OC
N155	A	0.971506569	CN1CCc2cc(c3cc2C1Cc1ccc(cc1)Oc1cc(CC2c4c(CCN2C)cc(c(c4O3)O)OC)ccc1OC)OC

N156	A	0.958607315	CCc1c[n+]2ccc3c4ccccc4[nH]c3c2cc1CCO
N157	A	0.93998315	C[n+]1cc2cc(c(cc2c2ccc3cc4c(cc3c12)OCO4)OC)OC
N160	A	0.920818754	CC(C)=CCC1=C(c2cc(c(cc2O)OC)O)Oc2cc(cc(c2C1=O)O)O
N161	A	0.892747838	CC=C(C)C(=O)O[C@@H]1[C@H]2C(C)=CC([C@H]([C@]2(C)[C@H]2C3([C@@H]([C@H](C)[C@@H]4CC(=O)O[C@H]1[C@]24CO3)O)O)O)=O
N162	A	0.892731713	CC1C2=CC=C3C(C)(CCC4(C)C5CC(CCC5(C)CCC34C)CO)C2=CC(C=1O)=O
N163	A	0.886056648	C1CN=C2C3=C(C(c4c2c1c[nH]4)=O)NCCC31C=C(C(C(=C1)[Br])O)[Br]
N164	A	0.874649898	CN1C2C(=Cc3ccccc13)N=c1ccccc1=2
N165	A	0.853871964	[H]C1C([H])C(C)(C)C2(C(C(C3([H])C(c4ccoc4CC3([H])C2(C)C1=O)=O)O)OC(C)=O)O
N166	A	0.853871964	CCC1CN2CCc3c4ccccc4[nH]c3C2CC1CC1c2c(CCN1)c1ccccc1[nH]2
N167	A	0.838631998	CC(C1CCC(C)(C)C23CCC(C2)C(C)(C)C=13)=O
N168	A	0.823908741	CC(=C)c1cc2C(c3ccccc3C(c2o1)=O)=O
N13	A	0.804898024	c1cc(ccc1[C@@H]1[C@H](C(c2ccc(cc2O1)O)=O)[C@@H]1C(c2ccc(cc2O[C@H]1c1ccc(cc1)O)O)=O)O
N169	A	0.804100348	[H][C@]1(C(c2ccc(cc2O[C@@]1([H])c1ccc(cc1)O)O)=O)[C@@]1([H])C(c2ccc(cc2O[C@]1([H])c1ccc(cc1)O)O)=O
N170	A	0.795880017	CC1C2=CC=C3C(C)(CCC4(C)C5CC(CCC5(C)CCC34C)CO)C2=CC(C=1O)=O
N171	A	0.795880017	CN1CCc2cc(c(cc2C1Cc1ccc(cc1)Oc1cc(CC2c3cc(c(cc3CCN2C)OC)O)ccc1O)O)OC
N172	A	0.793420088	CC1C2=CC=C3C(C)(CCC4(C)C5CC(CCC5(C)CCC34C)CO)C2=CC(C=1O)=O
N173	A	0.785156152	[H][C@@]1(c2cccc(c2C(c2c(cc(C)c(c3c(cc(c(C(C)=O)c3O)OC)O)c12)O)=O)O)c1ccc2C(c3c(c(C)cc(c3C(c2c1O)=O)O)c1c(cc(c(C(C)=O)c1O)OC)O)=O
N174	A	0.769551079	CC(=O)OC1CC2(C)C(CC3C2(C2(C)C1C1(C)C=CC(C(C)(C)C1=C(C2=O)O)=O)O3)c1ccoc1
N175	A	0.769551079	CN1CCc2cc(c(c3c2C1Cc1ccc(c(c1)Oc1ccc(CC2c4cc(c(cc4CCN=2)OC)O3)cc1)OC)OC)OC
N176	A	0.764888241	CN1CCc2cc(c3cc2C1Cc1ccc(c(c1)Oc1ccc(CC2c4c(CCN2C)cc(c(c4O3)O)OC)cc1)OC)OC
N177	A	0.763470341	CN1CCc2cc3c4cc2C1Cc1ccc(c(c1)Oc1ccc(CC2c5c(CCN2)cc(c(c5O4)O3)OC)cc1)OC
N179	A	0.744727495	CC(C)=CCc1c(cc(c2C(C3CC4c5c(C=3Oc12)c(cc(c5OC4(C)C)OC)O)=O)O)O

N180	A	0.744727495	CC1CCC2C(C3=C(C)CCC13C2(C)C)=O
N178	A	0.744727495	CCCCCCCCCCCCCCCC1CC(NCCCN(C)CCCCN(C)CCCN1C)=O
N181	A	0.721820802	CC1Cc2c(C(C)N1)c(cc(c2c1ccc(c2c(cc(C)cc12)OC)O)O)O
N182	A	0.721246399	[H]C1([H])[C@@H](C)[C@]2([H])C=CC([C@@]2(C)[C@H]([C@@H]2C(=C)C(=O)O[C@H]12)OC(C(=CC)[C@@]([H])(C)O)=O)=O
N187	A	0.698970004	C[C@@H]1[C@@H](C)C(c2cc(c(cc2[C@H]1c1ccc(c(c1)OC)OC)OC)OC)=O
N188	A	0.698970004	C[C@@H]1[C@@H](C)C(c2cc(c(cc2[C@H]1c1ccc(c(c1)OC)OC)OC)OC)=O
N183	A	0.698970004	C[n+]1cc2c(c3ccccc3[nH]2)c2ccccc12
N184	A	0.698970004	CC(=O)OC1C(C(C)(C)C2(CCC3C(Cc4c(cco4)C3=C)C2(C)C1OC(C)=O)O)OC(C)=O
N185	A	0.698970004	CC=C1CN2CCC34C2CC1C1C=C(C2CC56C7CC(C(CN27)=CC)C2=CCC(N(C25)c2ccccc26)=O)C(N(C13)c1ccccc14)=O
N186	A	0.698970004	CCCCCCCCCCCCCCCC1CC(NCCCN(C)CCCCN(C)CCCN1)=O
N189	A	0.690807656	CC1=CC(C(C2(C)C1CCC13COC4(C)C3C(=O)OC4C(C12)O)O)=O
N190	A	0.677780705	CC1=CC(C(C2(C)C1CC1C34COC(C)(C24)C(C(C3CC(=O)O1)O)O)O)=O
N191	A	0.670476574	CN1CCc2cc(c3cc2C1Cc1ccc(c(c1)Oc1ccc(CC2c4c(CCN2C)cc(c(c4O3)OC)O)cc1)OC)OC
N192	A	0.66756154	C(C(C(c1cnc(N)[nH]1)O)[Cl])N
N193	A	0.644740319	CC1C(C2(C3C4(C)C(CC5C3(CO2)C1(C(C(=O)O5)O)O)C(C)=CC(C4O)=O)O)O
N194	A	0.63685127	CN1CCc2cc(c3cc2C1Cc1ccc(c(c1)Oc1ccc(CC2c4c(CCN2C)cc2c(c4O3)OCO2)cc1)OC)OC
N195	A	0.633881109	CC1CC2C(C)(CCC3(C)C4=CC=C5C(=CC(C(=C5C)O)=O)C4(C)CCC23C)CC=1
N196	A	0.624827087	CC1Cc2cc(c(c3c(C)cc4cccc(c4c3O)OC)c(c2C(C)N1C)OC)OC
N197	A	0.619788758	[H]C1C([H])C(C)(C)C2(C(C(C3([H])C(=C([H])C(=O)OC)c4ccoc4CC3([H])C2(C)C1=O)O)OC(C)=O)O
N198	A	0.619788758	CC[C@H]1C=CCC[C@@]2(C[C@@H]3CC[C@@H]4[C@H](C(=O)OCCCCCCCCCCCCCCCCC(N(CCCN)C[C@H](CCN)O)=O)[C@]5(CCC[C@@H](C)O5)N=C(N2)N34)O1
N199	A	0.619788758	CCCCCCCCCCCCCCCC1CC(NCCCN(C)CCCCNCCCN1C)=O

N200	A	0.610129809	CC1Cc2cc(c(c3c(C)cc4cccc(c4c3O)OC)c(c2C(C)N1C)OC)O
N201	A	0.607976056	CC1Cc2cc(c(c3c(C)cc4cccc(c4c3O)OC)c(c2C(C)=N1)OC)OC
N202	A	0.602059991	CC1(C=O)C2CCC3(C)C2C=C2C(c4cc(ccc4C(C12O3)=O)O)=O
N205	A	0.585026652	C[C@@H]1C(c2cc(c(cc2[C@@H](c2ccc3c(c2)OCO3)[C@H]1C)OC)OC)=O
N204	A	0.585026652	CC(=O)OC1CC(C2(C)C3Cc4c(cco4)C(C3CCC2(C1(C)C)O)=O)OC(C)=O
N206	A	0.585026652	COc1ccc(cc1)C1=CC(=C)c2c(cc(c(c3cc(ccc3O)C3=CC(c4c(cc(cc4O3)OC)O)=O)c2O1)OC)O
N203	A	0.585026652	COc1ccc(cc1)C1=CC(c2c(cc(c(c3cc(ccc3O)C3=CC(c4c(cc(cc4O3)OC)O)=O)c2O1)OC)O)=O
N207	A	0.576829708	CN1CCc2cc(c3cc2C1Cc1ccc(cc1)Oc1cc(CC2c4c(CCN2C)cc(c(c4O3)OC)OC)ccc1OC)OC
N208	A	0.568636236	[H][C@@]1(c2cccc(c2C(c2c(cc(C)c(c3c(cc(c(C(C)=O)c3O)OC)O)c12)O)=O)O)c1ccc2C(c3c(c(C)cc(c3C(c2c1O)=O)O)c1c(cc(c(C(C)=O)c1O)OC)O)=O
N209	A	0.568636236	CC=C1CN2CCC34CC5C6CC7c8c(CC(C6COC5(C)OC45C2CC1C(C(=O)OC)N5c1ccccc13)N7C)c1ccccc1n8C
N210	A	0.568636236	CC1=CC(c2c(cccc2O)C1=O)=O
N211	A	0.568636236	CN1C2C(=C(C3=C4C(=c5ccccc5=N4)N(C)c4ccccc34)c3ccccc13)N=c1ccccc1=2
N212	A	0.566947523	CC1C2=CC=C3C(C)(CCC4(C)C5CC(=C)CCC5(CCC34C)CO)C2=CC(C=1O)=O
N213	A	0.552841969	CC(c1c(cc(c(c2c(C)cc(c3C(c4c(cccc4O)C(c23)=O)=O)O)c1O)OC)O)=O
N214	A	0.537602002	CCCCCCCC(CCCCCC1CC(NCCCN(C)CCCCN(C)CCCN1C)=O)O
N216	A	0.537117509	C[C@H]1[C@@H]([C@@H](C)[C@@](C)(O)O[C@@H]1[C@H]1CC[C@H]([C@@H]2CC[C@H]([C@]3(C)[C@@H]([C@@H](C)[C@]4([C@@H](C)[C@H](C[C@@H]([C@H]([C@](C)([C@H](C)OC)OC)O)O4)OC)O3)OC)O2)O1)O
N215	A	0.537117509	CC1C(C(C)C(C)(O)OC1[C@H]1CC[C@H]([C@@H]2CC[C@H]([C@]3(C)[C@@H]([C@@H](C)C4(C(C)C(CC([C@H]([C@](C)([C@H](C)OC)OC)O)O4)OC)O3)OC)O2)O1)O
N217	A	0.522878745	[H][C@@]1(CCCCCCCCC(O)=O)C[C@@](C)(CC(=O)OC)OO1
N218	A	0.522878745	[H]c1ccc(c2c1C(c1c(c(c(c(CCC=C(C)C)c1O)O)OC)N2C)=O)O
N12	A	0.52287874	c1c2C(=O)Oc3c4c(cc(c3O)O)C(=O)Oc(c24)c(c1O)O

		5	
N220	A	0.522878745	COc1ccc(cc1c1c(cc(c2C(=C)C=C(c3ccc(cc3)O)Oc12)O)O)C1=CC(c2c(cc(cc2O1)O)O)=O
N219	A	0.522878745	COc1ccc(cc1c1c(cc(c2C(C=C(c3ccc(cc3)O)Oc12)=O)O)O)C1=CC(c2c(cc(cc2O1)O)O)=O
N221	A	0.520947842	CCC1CN2CCc3cc(c(cc3C2CC1CC1=C2C=C(C(C=C2CCN1)=O)OC)OC)OC
N222	A	0.508505748	[H]C12CCC34C[C@@H](CCC3(C2(C)CCCC1(C)CO)OO4)C(C)C
N223	A	0.494850022	C[C@@H]1C(c2cc(c(cc2[C@@H](c2ccc(c(c2)OC)OC)[C@H]1C)OC)OC)=O
N224	A	0.494850022	C[C@@H]1C(c2cc(c(cc2[C@@H](c2ccc(c(c2)OC)OC)[C@H]1C)OC)OC)=O
N225	A	0.482489239	[H][C@@]12C[C@@H](C)CC[C@]2([H])C(C)(C)Oc2cc(C=Cc3ccccc3O)cc(c12)O
N226	A	0.48148606	CCCCCCCCCC(CCCCCC1CC(NCCCN(C)CCCCN(C)CCCN1)=O)O
N227	A	0.471564032	CC1C2=CC=C3C(C)(CCC4(C)C5CC(=C)CCC5(CCC34C)CO)C2=CC(C=1O)=O
N228	A	0.471548569	CCC1CC2CC3(C1N(CCc1c4cc(c(cc4[nH]c13)C1CC3C(C(Cc4c5ccccc5[nH]c14)N(C)CC3=CC)C(=O)OC)OC)C2)C(=O)OC
N229	A	0.46777671	CN1CCc2c(cc(c(c2O)OC)OC)C1Cc1ccc(cc1)Oc1c2CC3c4c(CCN3C)c(c(c(c4c2cc(c1OC)OC)OC)OC)O
N230	A	0.464001773	CC1Cc2c(C(C)N1C)c(cc(c2c1ccc(c2c(cc(C)cc12)OC)O)OC)O
N231	A	0.463441557	[H][C@@]12CCC3=C[C@]4(CC[C@@]3([C@@]1(C)CCC[C@@]2(C)CO)OO4)C(C)C
N232	A	0.462180905	CC1CCC2C(CC2(C)C)C(=C)CCC=1
N233	A	0.455931956	CN1CCc2cc(c3cc2C1Cc1ccc(cc1)Oc1cc(CC2c4c(CCN2C)cc(c(c4O3)OC)OC)ccc1OC)OC
N234	A	0.452225295	CN1CCc2cc(c3cc2C1Cc1ccc(cc1)Oc1c2C(Cc4ccc(c(c4)O3)O)N(C)CCc2cc(c1O)OC)OC
N235	A	0.448735828	CC1Cc2c(C(C)N1C)c(cc(c2c1ccc(c2c(cc(C)cc12)OC)OC)OC)O
N236	A	0.447699418	CC=C1CN2CCc3c4ccccc4[nH]c3C2CC1Cc1c2c(ccn1)c1cc(ccc1[nH]2)O
N237	A	0.446085152	C[C@@]12C[C@H]3[C@]4([C@@]5(C)C(C=C[C@@]6(C)CO[C@H]([C@H]56)[C@H]([C@@]4(C)C1=CC[C@H]2C1=CC(O)OC1=O)OC(c1ccccc1)=O)=O)O3
N238	A	0.443697499	CC=C1CN2CCC3c4ccccc4N4C(C1CC2C34C=CC1=COCC2C1CC1c3c(CC2N1C)c1ccccc1n3C)C(=O)OC
N239	A	0.443697499	CN1CCc2cc3c(c4c5ccccc5cc1c24)OCO3

N240	A	0.431798276	CC1=CC(C(=CC2=C1CC1CC(C3(C)C(CC4C(C)=CC(C(=CC=4O3)O)=O)CC(C)(C)C=CCC1(C)O2)O)O)=O
N241	A	0.430701672	CN1CCc2cc(c3cc2C1Cc1ccc(c(c1)Oc1ccc(CC2c4c(CCN2C)cc(c(c4O3)OC)OC)cc1)OC)OC
N242	A	0.429098948	CN1C(C(c2ccnc3c4cc(ccc4c(c1c23)OC)OC)=O)=O
N243	A	0.411037227	CC1CCC2C(C)(C)C(CCC2(C)C12Cc1c(cc(C=O)c(C=O)c1O2)O)O
N244	A	0.397940009	[H]C1C[C@@H]([C@]2(C)[C@@]3([H])Cc4c(cco4)[C@]([H])(C)C3([H])[C@H](C([H])[C@]2(C1(C)C)O)O)OC(C)=O
N245	A	0.397940009	CCC=CC(CC)CC1(C)CC(CC)C(CC(=O)OC)OO1
N246	A	0.396361892	CC1C2=CC=C3C(C)(CCC4(C)C5CC(C)(CCC5(C)CCC34C)C(O)=O)C2=CC(C=1O)=O
N247	A	0.387446099	CC1C2=CC=C3C(C)(CCC4(C)C5CC(C)(CCC5(C)CCC34C)C(=O)OC)C2=CC(C=1O)=O
N248	A	0.387216143	[H]C1([H])C(C)C2([H])C=CC([C@@]2(C)C([C@@H]2C(C)C(=O)O[C@H]12)OC(C(=C)[C@@]([H])(C)O)=O)=O
N249	A	0.384049948	CC1CCC2C(C)C3C(CC4C5CC=C6CC(CCC6(C)C5CCC34C)OC3C(C(C(C(CO)O3)O)OC3C(C(C(C(CO)O3)O)O)O)OC3C(C(C(C(C)O3)O)O)O)N2C1
N250	A	0.376559997	CC1C2=CC=C3C(C)(CCC4(C)C5CC(=C)CCC5(C)CCC34C)C2=CC(C=1O)=O
N251	A	0.332924511	COC1CC2C3(CCN2Cc2cc4c(cc23)OCO4)C2C1O2
N252	A	0.329096441	CC1Cc2c(C(C)N1C)c(cc(c2c1ccc(c2c(cc(C)cc12)OC)O)OC)O
N253	A	0.323928577	CCCCCC=CC(=O)OC1CC2C(CCCCC(CCC)O)CCCC2NC1C
N254	A	0.323880809	Cc1cc(c2c(ccc(c3c(cc(c4c3C[C@@H](C)N[C@@H]4C)O)O)c2c1)O)OC
N255	A	0.318758763	C1=C(c2ccc(cc2)Oc2c(cc(c3C(C=C(c4ccc(cc4)O)Oc23)=O)O)O)Oc2cc(cc(c2C1=O)O)O
N256	A	0.318758763	C1=C(c2ccc(cc2)Oc2c(cc(c3C(C=C(c4ccc(cc4)O)Oc23)=O)O)O)Oc2cc(cc(c2C1=O)O)O
N257	A	0.301029996	[H]c1c(C)cc(c2C(c3c(ccc(c3O)[C@@]3([H])c4cccc(c4C(c4c3cc(C)cc4O)=O)O)C(c12)=O)=O)O
N7	A	0.301029996	C[C@@]12CCC[C@]3(C)c4cc5C(C6=C(C(c5cc4C([C@]([C@H]23)(O)OC1)=O)=O)S(CCN6)(=O)=O)=O
N258	A	0.301029996	c1c2C(=O)Oc3c4c(cc(c3O)O)C(=O)Oc(c24)c(c1O)O
N259	A	0.301029996	CC1CC2C(Cc3c[nH]c4cccc2c34)N(C)C1
N260	A	0.30102999	CCC(C)C1C(C)C[C@H](C)C(C2=C(C(=CN(C2=O)O)

		6	c2ccccc2)O)O1
N261	A	0.301029996	CCN(CC)C(NC1CC2C(Cc3c[nH]c4cccc2c34)N(C)C1)=O
N262	A	0.301029996	CN1CCCC1c1c(ccc2c3CC[n+]4cc(C=C)c(CC5c6c(CCN5C)c5ccccc5[nH]6)c(c4c3[nH]c12)O)O
N263	A	0.294992041	CN1CCCC1c1c(ccc2c3CC[n+]4cc(C=C)c(CC5c6c(CCN5C)c5ccccc5[nH]6)c(c4c3[nH]c12)O)O
N265	A	0.293058232	CN1CCc2cc(c3cc2C1Cc1ccc(c(c1)Oc1ccc(CC2c4c(CCN2C)cc(c(c4O3)OC)O)cc1)OC)OC
N264	A	0.293058232	CN1CCc2cc(c3cc2C1Cc1ccc(c(c1)Oc1ccc(CC2c4c(CCN2C)cc(c(c4O3)OC)OC)cc1)O)OC
N266	A	0.276854339	CC1Cc2c(c3ccc(c4c(cc(C)cc34)O)OC)c(cc(c2C(C)N1)OC)O
N267	A	0.259637311	CC(C)=CCc1c2c(C=CC(C)(C)O2)c(c2C(C3CC4c5c(C=3Oc12)c(cc(c5OC4(C)C)O)O)=O)O
N268	A	0.251811973	C[C@@]12CC[C@@H]3[C@@]4(C)C(C=C[C@@]5(C)CO[C@H]([C@H]45)[C@H]([C@@]3(C)C1=CC[C@H]2c1ccoc1)O)=O
N269	A	0.250541126	COc1ccc2CC3c4cc5c(cc4CCN=3)Oc3c(cc4CCN[C@@H](Cc6ccc(cc6)Oc1c2)c4c3O5)OC
N270	A	0.249486661	C[n+]1ccc2c3cc(ccc3[nH]c2c1)[Cl]
N272	A	0.240558803	CC1CCC2(C(C)C3C(CC4C5CC=C6CC(CCC6(C)C5CCC34C)OC3C(C(C(C(CO)O3)O)OC3C(C(C(C(CO)O3)O)O)O)OC3C(C(C(C(C)O3)O)O)O)O2)NC1
N274	A	0.235749279	CC1C2=CC=C3C(C)(CCC4(C)C5CC(C)(CCC5(C)CCC34C)C(=O)OC)C2=CC(C=1O)=O
N276	A	0.22184875	C[C@@]12CC[C@@H]3[C@@]4(C)C(C=C[C@@]5(C)CO[C@H]([C@H]45)[C@H]([C@@]3(C)C1=CC[C@H]2c1ccoc1)O)=O
N277	A	0.22184875	CC(C)CC(=O)O[C@H]1C(=O)O[C@@H]2C[C@H]3C(C)=CC([C@H]([C@]3(C)[C@H]3[C@H]([C@@H]([C@]4(C(O)=O)[C@H]1[C@]23CO4)O)O)O)=O
N279	A	0.207608311	CC(C)CC(=O)O[C@@H]1[C@H]([C@H]2C(=C)C(=O)O[C@@H]2C[C@@H](C)C=CC([C@@]1(C)O)=O)O
N278	A	0.207608311	CC(C)CC(=O)O[C@H]1[C@H]2C(=C)C(=O)O[C@@H]2C[C@@H](C)C=CC([C@](C)([C@@H]1O)O)=O
N280	A	0.207608311	CC(C)CC(=O)OC1C2C(CC(C)C=CC(C(C)(C1OC(C)=O)O)=O)OC(C2=C)=O
N281	A	0.207608311	CCC(C)C(=O)OC1CC2C(C)(CC=C(C)C=C)C(C)CC(C23C(OC(C3=C1)OC(C)=O)OC(C)=O)O
N282	A	0.200659451	C[C@@H]1C(c2cc(c(cc2[C@@H](c2ccc(c(c2)OC)OC)[C@@H]1C)OC)OC)=O
N283	A	0.199838209	CC1=CC(C(C2(C)C1CC(C13COC4(C)C3C(=O)OC4C(C12)O)O)O)=O

N284	A	0.193820026	CC(C)[C@@H]1CCC2(C)C3C1C2C(C=C3C)=O
N285	A	0.193820026	COc1cc2c(CC[n+]3cc4cc(c(cc4cc23)OC)OC)cc1O
N286	A	0.193625017	CC(C)C=Cc1c(cc(C=Cc2ccc(cc2O)O)cc1O)O
N23	A	0.193625002	CC(C)C=Cc1c(cc(C=Cc2ccc(cc2O)O)cc1O)O
N287	A	0.187822226	CC1CCC2(C(C)C3C(CC4C5CC=C6CC(CCC6(C)C5CCC34C)OC3C(C(C(C(CO)O3)OC3C(C(C(C(C)O3)O)O)O)O)OC3C(C(C(C(C)O3)O)O)O)O2)NC1
N288	A	0.187086643	[H][C@]12[C@H](C[C@@]3([H])C(C)(C)[C@H](CC[C@]3(C)[C@@]2([H])CC=C(C=C)[C@@H]1C)O)O
N289	A	0.187086643	[H]C1C([H])C(C)(C)C2(C(C([H])C3([H])C(=C([H])C(=O)OC)c4ccoc4CC3([H])C2(C)C1=O)OC(C)=O)O
N290	A	0.185419484	CC1CCC2(C(C)C3C(CC4C5CCC6CC(CCC6(C)C5CCC34C)OC3C(C(C(C(CO)O3)OC3C(C(C(C(CO)O3)O)OC3C(C(C(CO3)O)O)O)OC3C(C(C(C(CO)O3)O)O)O)O)O)O2)NC1
N291	A	0.180456065	CC(C)=CCC1=C(c2cc(c(cc2OC)OC)O)Oc2cc(cc(c2C1=O)O)O
N292	A	0.178493785	C[C@]12C(C=C[C@@]3(C)COC(=C[C@]4(C)[C@@]25[C@H](C[C@@]2(C)[C@@H](C[C@@H]6[C@@]24O6)C2=CC(O)OC2=O)O5)[C@H]13)=O
N293	A	0.173925197	CC(C)=CCCC(C)=CCCC(C)(C=C)c1ccc(c(c1)O)O
N294	A	0.173925197	CC(C)=CCCC(C)=CCCC(C)(C=C)c1ccc(c(c1)O)O
N295	A	0.15490196	CC(C)=CCc1c(cc2c(C(c3c4C=CC(C)(C)Oc4c(c(CC=C(C)C)c3O2)O)=O)c1O)O
N296	A	0.15490196	CCC(C)C1C2=NC(C(C)O2)C(NCc2nc(cs2)C(NC(C(C)CC)c2nc(cs2)C(N1)=O)=O)=O
N297	A	0.152518716	CC(c1c(cc(c(c2c(C)cc(c3C(c4c(cccc4O)C(c23)=O)=O)O)c1O)O)O[C@H]1[C@@H]([C@H]([C@@H]([C@@H](CO)O1)O)O)O)=O
N298	A	0.148741651	CN1CCc2cc(c(c3c2C1CC31CCC2(C(C1)OC)c1c(CCN2)c2cc(c(cc2[nH]1)OC)OC)O)OC
N299	A	0.146423456	CC1CCC2C(C)C3C(CC4C5CC=C6CC(CCC6(C)C5CCC34C)OC3C(C(C(C(CO)O3)OC3C(C(C(C(C)O3)O)O)O)O)OC3C(C(C(C(C)O3)O)O)O)N2C1
N300	A	0.128614762	C1CCN2CCC3C(=CC(CCC=CC1)(C1C3(CC3C=CCCCN13)C2)O)c1c2c(ccn1)c1cc(ccc1[nH]2)O
N302	A	0.099882403	COc1cc2CC[n+]3cc4c(ccc(c4OC)OC)cc3c2cc1OC
N303	A	0.096910013	[H]C1CC(C)(C)C2(C(C3C4([H])C(C(=O)O3)c3ccoc3CC4([H])C2(C)C1=C([H])OC(C)=O)OC(C)=O)O

N305	A	0.096910013	C(C(=O)O[C@H]1[C@@H]([C@@H](COC(CC2(C=CC(C=C2)=O)O)=O)O[C@H]([C@@H]1OC(Cc1ccc(cc1)O)=O)OC(CC1(C=CC(C=C1)=O)O)=O)O)c1ccccc1
N304	A	0.096910013	C(C(=O)O[C@H]1[C@@H]([C@@H](COC(CC2(C=CC(C=C2)=O)O)=O)O[C@H]([C@@H]1OC(Cc1ccccc1)=O)OC(CC1(C=CC(C=C1)=O)O)=O)O)c1ccc(cc1)O
N306	A	0.096910013	C1CCN[C@H]2[C@@]3(CCC=CCCCCN4CC[C@@H](C(=C3)[C@@H]3c5c(CCN3CCc3c6c(ccn3)c3ccccc3[nH]6)c3ccccc3[nH]5)[C@]2(CCC=CC1)C4)O
N308	A	0.096910013	CC(=O)OC1CC(C)(C)C2(CCC3C(Cc4c(cco4)C3=O)C2(C)C1OC(C)=O)O
N309	A	0.096910013	CC(=O)OC1CCC(C)(C)C2(CC3C4C(C(=O)O3)c3ccoc3CC4C12C)O
N307	A	0.096910013	CC(C)=CCc1cc(C(C=Cc2ccc(cc2)O)=O)c(c(CC=C(C)C)c1O)O
N310	A	0.096910013	CC=C(C)C(=O)OC1CC2(C)C(C=C(C(CO)=CC3C1C(=C)C(=O)O3)O2)=O
N311	A	0.096910013	CC1CC2C(C)(CCC3(C)C4=CC=C5C(=CC(C(=C5C)O)=O)C4(C)CCC23C)C(C1=O)O
N313	A	0.096910013	CN1C2=CC(c3ccccc3NC2c2ccccc12)=O
N312	A	0.096910013	CN1C2C(=C(c3ccccc13)n1c3ccccc3c3c1cc1ccccc1n3)c1ccccc1N=2
N314	A	0.049635146	CN1CCc2cc(c(c3c2C1Cc1ccc(cc1)Oc1c2C(Cc4ccc(cc4)O3)N(C)CCc2cc(c1O)OC)O)OC
N315	A	0.045757491	C[C@@]12CCC[C@]3(C)c4cc5C(C6=C(C(c5cc4C([C@]([C@H]23)(O)OC1)=O)=O)S(CCN6)(=O)=O)=O
N316	A	0.045757491	COc1cc2c(CC[n+]3cc4cc(c(cc4cc23)OC)OC)cc1O
N317	A	0.036212173	CC(C)CC(=O)O[C@@H]1C[C@@](C)(C(C=C[C@H](C)C[C@@H]2[C@@H]1C(=C)C(=O)O2)=O)O
N318	A	0.023552065	CC(C)C(=O)OC1CC(C)(C=CC(C(C)=CC2C1C(=C)C(=O)O2)=O)O
N319	A	0.022898582	CCCCCC=CC(=O)OC1CC2C(CCCCC(CCC)O)CCCC2NC1C
N320	A	0.017728767	[H][C@]1(C(c2c(cc(cc2O[C@@H]1c1ccc(cc1)O)O)O)=O)[C@@]1([H])C(c2c(cc(cc2O[C@@H]1c1ccc(cc1)OC)O)O)=O
N321	A	0.013228266	CC(C)=CCC(=O)OC1CC2C(CC=C3CC(CCC23C)N(C)C)C2CCC3CN(C)CC123
N322	A	0.013084098	COc1ccc(cc1)C1C(C2C(c3ccc(cc3)O)Oc3cc(cc(c3C2=O)O)O)C(c2c(cc(cc2O1)O)O)=O
N323	A	0.008773924	[H][C@]12CC[C@]3(C)[C@]([H])(CC[C@]4([H])[C@@]5([H])[C@@H](CC[C@@]5(CC[C@@]34C)C(O)=O)C(C)=C)[C@@]2(C)CC[C@@H](C1(C)C)OC(C)C=

			Cc1ccc(c(c1)O)O
N335	A	0	[C-]#[N+]C(C)(C)C[C@H]1CC(C)C2CCC(C)C3(CCC(=C)C1[C@H]23)[N+]#[C-]
N326	A	0	[H][C@@]1(C(c2c(cc(cc2O[C@@H]1c1ccc(cc1)O)O)O)=O)[C@@]1([H])C(c2c(cc(cc2O[C@@H]1c1ccc(cc1)OC)O)O)=O
N327	A	0	[H]C1C([H])C(C)(C)C2(C(C(C3([H])C(=C([H])C(=O)OC)c4ccoc4CC3([H])C2(C)C1=O)OC(C)=O)OC(C)=O)O
N328	A	0	[H]C1C([H])C(C)(C)C2(C(C(C3([H])C(c4ccoc4CC3([H])C2(C)C1=O)=O)OC(C)=O)OC(C)=O)O
N331	A	0	C(C(=O)O[C@@H]1[C@H]([C@@H]([C@@H](COC(CC2(C=CC(C=C2)=O)O)=O)O[C@H]1OC(CC1(C=CC(C=C1)=O)O)=O)O)O)c1ccccc1
N332	A	0	C1=C(c2c[nH]c3ccc(cc23)O)NC(C1=C1C(Nc2ccccc12)=O)=O
N333	A	0	C1C(C(c2ccc(c(c2)OC(c2cc(c(c(c2)O)O)O)=O)O)Oc2cc(cc(c12)O)O)O
N329	A	0	CC(=C)C[C@@H]1C[C@H](C)[C@H]2CC[C@](C)(C#N)[C@H]3CCC(=C)[C@@H]1[C@H]23
N330	A	0	CC(C)=CC1C[C@H](C)[C@H]2CC[C@H](C)[C@]3(CC[C@H](C)C=1[C@H]23)C#N
N334	A	0	CC1=CC(C(C2(C)C1CC1C34COC(C23)(C(C(=C)C4(C(C(=O)O1)O)O)O)O)O)OC(C1C(C(C(C(O1)OC1(CO)C(C(C(CO)O1)O)O)O)O)O)O
N336	A	-0.002710169	COc1ccc(cc1)C1C(C2C(c3ccc(cc3)O)Oc3cc(cc(c3C2=O)O)O)C(c2c(cc(cc2O1)O)O)=O
N337	A	-0.012281092	CC1C2CC3C45COC(C5C(C(=O)O3)O)(C(C(C4C2(C)CC(C=1O)=O)O)O)C(=O)OC
N338	A	-0.01466708	COc1cc2c(CC[n+]3cc4c(ccc(c4OC)OC)cc23)cc1O
N339	A	-0.015046813	CCCC(=O)O[C@H]1[C@H]2c3cc4c(cc3CN3CCC(=C[C@@H]1O)[C@H]23)OCO4
N340	A	-0.017033339	CC(C)=CCC1=C(c2cc(c(cc2O)O)O)Oc2c(CC=C(C)C)c3c(C=CC(C)(C)O3)c(c2C1=O)O
N341	A	-0.017033339	CC1C2CCC3C4CC=C5CC(CCC5(C)C4CCC23CN1C)N(C)C
N342	A	-0.028421338	COc1cnc2c3ccccc3C(c3c2c1ccn3)=O

N343	A	-0.041392685	[H]N1CCS(C2=C1C(c1cc3c(cc1C2=O)C(c1c2c(co1)C(CC[C@]23C)=O)=O)=O)(=O)=O
N344	A	-0.041392685	CC(C)=CCc1c(c2C(c3ccc(c(c3Oc2c(c1O)C(C)(C)C=C)O)O)=O)O
N346	A	-0.041392685	CC12CCCc3coc(C(c4cc5C(C=CC(c5cc14)=O)=O)=O)c23
N345	A	-0.041392685	CCC=C(C)C=CCCCC1(C[C@H](C)[C@H](C(C)C(=O)OC)OO1)OC
N347	A	-0.041392685	CN1CCc2cc3c4cc2C1Cc1ccc(c(c1)Oc1ccc(CC2c5c(CCN2C)cc(c(c5O4)O3)OC)cc1)O
N348	A	-0.049218023	CC(C)=CCC1=C(c2cc(c(cc2O)O)O)Oc2c(cc(CCC=C(C)C)c(c2CCC=C(C)C)O)C1=O
N349	A	-0.053078444	CC(C)=CCC(=O)OC1CC2C(CC=C3CC(CCC23C)N(C)C)C2CCC3CN(C)CC123
N350	A	-0.056904851	CN1CCc2cc(c3cc2C1Cc1ccc(c(c1)c1cc(CC2c4c(CCN2C)cc(c(c4O3)OC)OC)ccc1OC)O)OC
N351	A	-0.0651105	COc1ccc2c3ccnc(CCC(O)=O)c3[nH]c2c1
N352	A	-0.079181246	C1C([C@@H](c2ccc(c(c2)O)O)Oc2cc(cc(c12)OC(c1cc(c(c(c1)O)O)O)=O)O)O
N353	A	-0.079181246	CC(C)(C=C)c1c2c(C=CC(C)(C)O2)c(c2C(c3ccc(c(c3Oc12)O)O)=O)O
N354	A	-0.079181246	CC(C)CC(=O)O[C@H]1C(=O)O[C@@H]2C[C@H]3C(C)=CC([C@H]([C@]3(C)[C@H]3[C@H]([C@@H]([C@]4(C(=O)OC)[C@H]1[C@]23CO4)O)O)O)=O
N355	A	-0.079181246	COc1ccc(cc1O)C1=COc2cc(ccc2C1=O)O
N19	A	-0.08278537	CC(=O)OC1CC2C(C)(C)C(C=CC2(C)C2CCC3(C)C(CC=C3C12C)c1ccoc1)=O
N356	A	-0.105873535	CC1Cc2c(C(C)N1)c(cc(c2c1ccc(c2c(cc(C)cc12)OC)O)OC)OC
N357	A	-0.10720997	[H][C@]1(Cc2cc(C)c(c(c2)Oc2ccc(C[C@]3([H])c4cc(c(cc4CCN3C)OC)O)cc2)OC)c2cc(c(cc2CCN1C)OC)O
N358	A	-0.11394335	[H][C@@]12c3c(cc(c(c3O)O)O)C(=O)O[C@@]1([H])[C@@H]([C@H]([C@H](CO)O2)O)OC(c1cc(c(c1)O

		2	C)O)O)=O
N362	A	-0.113943352	CC(C)=CCc1c(c(CC=C(C)C)c2c(C(c3cc(c(c(c3O2)O)OC)OC)=O)c1O)O
N359	A	-0.113943352	CC(C)=CCc1c(cc2c(C(C3=C(C=C(C(C3(CC=C(C)C)CC=C(C)C)=O)O)O2)=O)c1O)O
N360	A	-0.113943352	CC(C)=CCCC(C)=CCc1c(cc(c2C(c3cc(ccc3Oc12)O)=O)O)O
N361	A	-0.113943352	Cc1cc(c2c(ccc(c3ccc4cc(C)nc(C)c4c3O)c2c1)OC)OC
N363	A	-0.117271296	CC(C)=CCc1c(cc(c2C(CC(c3c(cc(cc3OC)OC)OC)Oc12)=O)O)O
N364	A	-0.117271296	CC(C)=CCCC(C)=CCc1c2c(C=CC(C)(C)O2)c(c2C(C3CC4c5c(C=3Oc12)c(cc(c5OC4(C)C)O)O)=O)O
N365	A	-0.118641915	CC(C)(C=C)C1=Cc2c(c3C=CC(C)(C)Oc3c(c2OC1=O)C(C)(C)C=C)O
N366	A	-0.119202473	CN1CCc2cc(c(cc2C1Cc1ccc(cc1)Oc1c2CC3c4c(CCN3C)c(c(c(c4c2cc(c1O)OC)OC)OC)OC)OC)O
N367	A	-0.119976167	CC1CC(C=C(C)C)OC12CCC1(C)CC3C(C(=CCC12)C=O)C(CC3(C)O)=O
N368	A	-0.120652467	CC1Cc2c(c3ccc(c4c(cc(C)cc34)O)OC)c(cc(c2C(C)N1C)OC)O
N369	A	-0.125951907	CC=C1CN(C)C2Cc3c4ccccc4[nH]c3C(CC1C2C(=O)OC)=O
N370	A	-0.131595572	CCC=CC(CC)CCCC1(CC)CC(CC)C(CC(=O)OC)OO1
N371	A	-0.136720567	CC(C)=CCCC(C)=CCCC(C)=CCc1cc(ccc1O)O
N373	A	-0.146128036	C=CC12CC(C3C(C2C(=C)C(=O)OC1)OC(C3=C)=O)OC(C(=C)CO)=O
N372	A	-0.146128036	CC(C)=CCc1c(c2C(c3ccc(c(c3Oc2c2c1O[C@@H](C)C2(C)C)O)O)=O)O

N374	A	-0.146128036	CC(C)c1cc2C(CC3C(C)(C)CCCC3(C)c2cc1O)=O
N375	A	-0.146128036	CCCCCCCCCC(C#CC#CC(C=C)OC(C)=O)O
N376	A	-0.146128036	CCCCCCCCCC(C#CC#CC(C=C)OC(C)=O)O
N377	A	-0.146128036	CNCCc1cc(c(c(c1)[Br])OCCCNC(C1CC2(CC(=C(C(=CO2)[Br])OC)[Br])ON=1)=O)[Br]
N378	A	-0.146128036	COc1ccc(cc1)C1=CC(c2c(cc(c(c3cc(ccc3OC)C3=CC(c4c(cc(cc4O3)OC)O)=O)c2O1)O)O)=O
N379	A	-0.148943737	CC1CC(C)OC(CC(c2ccc(cc2)O)NC(C(Cc2c3ccccc3[nH]c2[Br])N(C)C(C(C)NC(C(C)CC(C)=C1)=O)=O)=O)=O
N380	A	-0.150315299	CC=C1CN2C3CC1C(COC(c1cc(c(c(c1)OC)OC)OC)=O)(C(=O)OC)C14CC2OC34N(C)c2cc(c(cc12)OC)OC
N381	A	-0.16774337	CCC1CC2CC3(C1N(CCc1c4ccccc4[nH]c13)C2)C(=O)OC
N382	A	-0.173776353	CC(=O)OC1CC2C(C)(C)C(C=CC2(C)C2CCC3(C)C(c4ccoc4)OC(C4C3(C12C)O4)=O)=O
N384	A	-0.176091259	C(C(=O)O[C@H]1[C@@H]([C@@H](COC(CC2(C=CC(C=C2)=O)O)=O)O[C@H]([C@@H]1OC(Cc1ccccc1)=O)OC(CC1(C=CC(C=C1)=O)O)=O)O)c1ccccc1
N383	A	-0.176091259	C[C@H]1C[C@H]2C[C@@H](C)[C@@](C)(C#N)[C@H]3CC[C@H]4[C@H]([C@H]23)[C@@H]1CC[C@]4(C)NC=O
N395	A	-0.176091259	CC(=C)C1CCC2(CCC3(C)C(C)(CCC4C3(C)CCC3C(C)(C)C(C(C[C@@]34C)O)OC(C=Cc3ccc(cc3)O)=O)C12)C(O)=O
N396	A	-0.176091259	CC(C)[C@]12CC[C@](C)(C3=C([C@H](C)CC3=O)[C@H]1O)OO2
N394	A	-0.176091259	CC(C)=CCc1c(cc2cc3CC(C)(CC(c3c(c2c1O)O)=O)O)OC
N387	A	-0.176091259	Cc1c2c(ccn1)c1ccccc1[nH]2
N386	A	-0.17609125	CC1CCC(C(=C)CC2C(C(C=1)O)C(=C)C(=O)O2)O

		9	
N385	A	-0.176091259	CC1CCC(C(C)=CC2C(C(C=1)O)C(=C)C(=O)O2)O
N391	A	-0.176091259	CN1CCc2cc(c(c3c2C1Cc1ccc(cc1)Oc1c2C(Cc4ccc(cc4)O3)N(C)CCc2cc(c1OC)OC)OC)OC
N388	A	-0.176091259	CN1CCc2cc(c(cc2C1Cc1ccc(c(c1)O)OC)Oc1cc(c(cc1CC1c2cc(c(cc2CCN1C)OC)O)OC)OC)OC
N389	A	-0.176091259	CN1CCc2cc(c(cc2C1Cc1ccc(cc1)Oc1cc(CC2c3cc(c(cc3CCN2C)OC)OC)ccc1OC)O)OC
N392	A	-0.176091259	CN1CCc2cc(c3c(c2C1Cc1ccc(c(c1)Oc1cc2c(ccnc2Cc2ccc(CO3)cc2)cc1OC)O)O)OC
N393	A	-0.176091259	CN1CCc2cc(c3c(c2C1Cc1ccc(c(c1)Oc1cc2c(ccnc2Cc2ccc(CO3)cc2)cc1OC)OC)O)OC
N390	A	-0.176091259	CN1CCc2cc(c3cc2C1Cc1ccc(c(c1)Oc1ccc(CC2c4c(CCN2C)cc(c(c4O3)OC)OC)cc1)O)OC
N9	A	-0.178318047	C[C@H]([C@H]1[C@H](C=C[C@H]2[C@H]([C@@H](C=C)O)O2)O1)O
N397	A	-0.181843588	COc1ccc2CC3c4cc5c(cc4CCN=3)Oc3c(cc4CCN[C@@H](Cc6ccc(cc6)Oc1c2)c4c3O5)OC
N398	A	-0.185238094	CC(C)(C=C)c1c2c(C=CC(=O)O2)c(c2C=CC(C)(C)Oc12)OC
N399	A	-0.187520721	CC(C)[C@@H]1CCC(C)=CCC[C@](C)(C=C1)O
N400	A	-0.188190065	CC(c1c(cc(c(c2c(C)cc(c3C(c4c(cccc4O)C(c23)=O)=O)O)c1O)O)OC)=O
N401	A	-0.193246024	CCC(CC1(CC)C=C(CC)C(=CC(=O)OC)O1)C=CC(C)=O
N402	A	-0.195148636	CCCC(=O)O[C@H]1C=C2CCN3Cc4cc5c(cc4[C@@H]([C@@H]23)[C@@H]1OC(CCC)=O)OCO5
N403	A	-0.195274365	C[C@@]12CC[C@@]3(C[C@@]2(CCC2[C@@]14CCC[C@@]2(C)C(=O)O4)C(C3=C)=O)OC

N404	A	-0.201162722	CC1C(CC2C=1C1(C)C(CC(O)=O)C3(C)C(C(C1O2)O)C(C)(C=CC3=O)C(=O)OC)c1ccoc1
N405	A	-0.204119983	[H][C@@]12C3CCC1=C[C@@H]([C@H]([C@@]2([H])c1cc2c(cc1C3)OCO2)OC(C[C@H](C)O)=O)O
N406	A	-0.204119983	CC(=O)OC(C)(C)C=CC(C(C)(C1C(CC2(C)C3CC=C4C(CC(C(C4(C)C)=O)O)C3(C)C(CC12C)=O)O)O)=O
N407	A	-0.204119983	CC(=O)OC1C2C(C(=O)OC)c3ccoc3CC2C2(C)C(CCC(C)(C)C2(C1O)O)=O
N409	A	-0.204119983	CC1(CC(c2c(C1)cc1cc(c3C=CC(C)(C)Oc3c1c2O)OC)=O)O
N408	A	-0.204119983	COc1cc2C3Cc4cc(c(cc4CN3CCc2cc1O)OC)OC
N410	A	-0.205977299	C[C@@H](CC(=O)O[C@H]1[C@H]2c3cc4c(cc3CN3CCC(=C[C@@H]1O)[C@H]23)OCO4)O
N411	A	-0.212187604	CC(C)[C@@H]1CCC(=C)[C@@H](CC[C@@](C)(C=C1)O)O
N412	A	-0.212187604	CN1CCc2cc(c3c4c2C1Cc1ccc(cc1)Oc1cc(CC2c5cc(c(cc5CCN=2)O3)O4)ccc1O)OC
N17	A	-0.217483944	C(c1c2c(cc[nH]2)c2ccccc2n1)n1cnc2c(N)ncnc12
N413	A	-0.217577675	CC(=O)OC1CC2C(C)(C)C(C=CC2(C)C2CCC3(C)C(C(C4C3(C12C)O4)=O)c1ccoc1)=O
N414	A	-0.220217738	C[C@@H](CC(=O)O[C@H]1[C@H]2c3cc4c(cc3CN3CCC(=C[C@@H]1O)[C@H]23)OCO4)O
N415	A	-0.220270953	CC1Cc2cc(c(c3c(C)cc4cccc(c4c3O)OC)c(c2C(C)N1C)OC)OC
N416	A	-0.225309282	CCC=C(C)CCC=C(C)Oc1cc2Cc3cc(C)cc(c3C(c2c(c1)O)=O)O
N417	A	-0.230448921	[H]C1C([H])[C@]2(C(C)(C)C([H])[C@H](CC(C)=O)C(=C([H])OC(C)=O)[C@]2(C)[C@@]2([H])Cc3c(cco3)C(=C)C12[H])O
N420	A	-	CC(C)=CCCC(C)=CCc1c(cc2cc3CC(C)(CC(c3c(c2c1

		0.230448921	O)O)=O)O)OC
N418	A	-0.230448921	CCC(C)C(=O)OC1(c2cccc(c2C(c2c1cc(c(C(=O)OC)c2C)O)=O)O)c1ccc2C(c3cc(c(C(=O)OC)c(C)c3C(c2c1O)=O)O)=O
N419	A	-0.230448921	COc1cc(ccc1O)C1C(CO)Oc2ccc(cc2O1)C1=C(C(c2c(cc(cc2O1)O)O)=O)O
N421	A	-0.234968231	CC1Cc2cc(c(c3c(C)cc4cccc(c4c3O)OC)c(c2C(C)N1C)OC)O
N422	A	-0.235976009	[H]c1ccccc1C=Cc1cc(c2c(c1)OC(C)(C)[C@@]1([H])CC[C@H](C)C[C@@]12[H])O
N423	A	-0.237121983	CC1Cc2cc(c(c3c(C)cc4cccc(c4c3O)OC)c(c2C(C)=N1)OC)OC
N20	A	-0.240549248	CC(=O)OC1CC(C(C)(C)C2CC(C3(C)C(CCC4(C)C(CC=C34)C3CC(O)OC3)C12C)OC(C)=O)=O
N424	A	-0.242114053	[H][C@@]1(C[C@@H](C)CC[C@]1([H])C(C)C)c1c(cc(C=Cc2ccccc2O)cc1O)O
N425	A	-0.246673236	CC(=CCO)C1CC2C(C)(C)C(C=CC2(C)OO1)=O
N426	A	-0.255272505	[H][C@](CO)(C#CC#CC#CC#CC#CC)O
N427	A	-0.255272505	C1CCN2CCC3C(=CC(CCC=CC1)(C1C3(CC3C=CCCCN13)C2)O)c1c2c(ccn1)c1ccccc1[nH]2
N428	A	-0.255272505	CC(C)=CCC1=C(C(CC=C(C)C)(CC=C(C)C)c2cc3c(c(C)cc(c3c(c2C1=O)O)O)O)O)O
N429	A	-0.257318214	c1ccc2c(c1)C(c1c3c(ccn1)ccnc23)=O
N430	A	-0.264817823	CC1=C(C(C=C(C1=O)OC)=O)[Cl]
N431	A	-0.265200973	C[C@@H]1CC[C@@]2(CC[C@]3(C)C(=CC[C@@H]4[C@@]5(C)C[C@H]([C@@H](C(C)(C)[C@@H]5CC[C@@]34C)O)O)[C@@H]2[C@]1(C)O)C(O)=O
N432	A	-0.27067883	C[C@]12CC[C@H]3C(=C)C(=O)O[C@@H]3[C@H]2C(=C)C(C=C1)=O

		6	
N434	A	-0.286478362	CC(=O)OC1CC2C(C)(C)C(C=CC2(C)C2CCC3(C)C(CC=C3C12C)C1CC(C2C(C)(C)O2)OC1OC(C)=O)=O
N435	A	-0.29666519	CC(C)=CCOc1cc2Cc3cc(C)cc(c3C(c2c(c1)O)=O)O
N3	A	-0.301029996	C[C@]12C[C@H]3[C@H](C[C@H]4[C@@](C)(CC[C@H]1O2)O4)C(=C)C(=O)O3
N436	A	-0.301029996	C1=C(C(c2c(cc(cc2O1)O)O)=O)c1ccc(cc1)O
N437	A	-0.301029996	C1CN=C2C3=C(C(c4c2c1c[nH]4)=O)NCCC31C=C(C(C(=C1)[Br])=O)[Br]
N438	A	-0.301029996	CC(=O)O[C@H]1[C@H](C(C)(C)[C@@]2(CC[C@H]3C(c4ccoc4C[C@@H]3[C@@]2(C)[C@H]1OC(C)=O)=O)O)OC(C)=O
N439	A	-0.301029996	CC1=CC(C(C2(C)C1CC1C34COC(C23)(C(C(=C)C4(C(C(=O)O1)O)O)O)O)O)O
N443	A	-0.301029996	CC12CCC(C)(CC2C2(C)CCC3=C(CCC4C(C)(C)C(CCC34C)O)C2(C)CC1)C(O)=O
N442	A	-0.301029996	Cc1cc(c2c(ccc(c3c(cc4C[C@H](C)N(C)[C@H](C)c4c3OC)OC)c2c1)OC)OC
N440	A	-0.301029996	CCCCC(C)CC(C)C(N1CCCC1C(=O)OC(C(C)CC)C(=O)OC(C(C)CC)C(O)=O)=O
N441	A	-0.301029996	COc1cc(c2C(C=C(c3ccc(c(c3)c3c(cc(c4C(C=C(c5ccc(cc5)O)Oc34)=O)O)O)OC)Oc2c1)=O)O
N444	A	-0.303383901	COC(c1cc(c(c(c1)O)O)O)=O
N445	A	-0.307011106	C[C@@]12CC[C@@]3(C[C@@]2(CCC2[C@@]14CCC[C@@]2(C)C(=O)O4)C(C3=C)=O)O
N446	A	-0.309630167	COc1ccc(cc1OC)C(CNC(C=Cc1ccccc1)=O)OC(C=Cc1ccccc1)=O
N447	A	-0.309832294	CC(C)C1C(N(C)C(Cc2ccccc2)C(=O)OC(C(C)C)C(N(C)C(Cc2ccccc2)C(=O)OC(C(C)C)C(N(C)C(Cc2ccccc2)C(=O)O1)=O)=O)=O
N448	A	-	CC(C)=CCCC(C)=CCc1c(CC=C(C)C)c2C(c3c(cc(cc3

		0.315238666	Oc2c(c1O)O)O)O)=O
N449	A	-0.319799783	C1CN2Cc3cc4c(cc3C3C2C1=CC(C3O)O)OCO4
N450	A	-0.322219295	[H]C1CC([H])[C@]2(C)[C@@]3([H])Cc4c(cco4)[C@]([H])(C)C3([H])[C@H](C([H])[C@]2(C1(C)C)O)O
N452	A	-0.322219295	CC(=C)C(CC=C(C)C)Cc1c(cc(c2C(C=C(c3ccc(cc3OC)O)Oc12)=O)O)O
N451	A	-0.322219295	CC(=C)C(CC=C(C)C)Cc1c(cc(c2C(CC(c3ccc(cc3OC)O)Oc12)=O)O)O
N453	A	-0.322219295	COc1cc(ccc1O)[C@@H]([C@H](CO)Oc1c(cc(CCCO)cc1OC)OC)O[C@H]1[C@@H]([C@@H](C(C(CO)O1)O)O)O
N454	A	-0.328379603	C[C@H]1[C@H]([C@H](C(C(Oc2cc3c(C(CC(c4ccc(c(c4)O)O)O3)=O)c(c2O)O)O1)O)O)O
N455	A	-0.336459734	CCCCCCCCCC(C#CC#CC(C=C)OC(C)=O)O
N15	A	-0.336834312	C1C(O)OCC2=C1C(c1ccccc1C2=O)=O
N456	A	-0.342422681	CC(=C)C(Cc1cc(C(C=Cc2ccc(c(CC=C(C)C)c2)O)=O)c(cc1O)O)O
N459	A	-0.342422681	CC(=O)OCCCCCCCCCCC(C#CC#CC(C=C)OC(C)=O)O
N458	A	-0.342422681	CC(C(CCCCCCCCCC(C#CC#CC(C=C)OC(C)=O)O)=O)=O
N457	A	-0.342422681	CC(C)=CCC1CC23CC(CC=C(C)C)C(C)(C)OC2=C(C(c2ccc(c(c2)O)O)=O)C(C(CC=C(C)C)(C3=O)C1(C)C)=O
N460	A	-0.342422681	CC(C)C[C@@H]([C@@H]1Cc2cccc(c2C(=O)O1)O)NC([C@H]([C@@H]1[C@@H]2CC(N[C@](C)([C@@](C)(O)O1)N2)=O)O)=O
N461	A	-0.348304863	[H]C(=C)C1=CCCC(CO)=C[C@H]2[C@H](C(=C)C(=O)O2)[C@H](CC(C(C)CC)=O)[C@@H]1OC(C)=O
N462	A	-0.35554216	CC1Cc2c(c3ccc(c4c(cc(C)cc34)O)OC)c(cc(c2C(C)N1C)OC)O

		3	
N463	A	-0.358235642	CC=C1CN2CCC34C(C2CC1C4(COC(C)=O)C(=O)OC)=Nc1ccccc13
N464	A	-0.361727836	CC1CC2=CC(C=C3C2=C(C=C(C)O3)O1)=O
N465	A	-0.361727836	CCC(C)C1c2nc(cs2)C(NC(C)C2=NC(C(C)O2)C(NC(C(C)C)c2nc(co2)C(N1)=O)=O)=O
N466	A	-0.366045493	COC1CC2C3(C=C1)C(CN2Cc1cc2c(cc13)OCO2)O
N467	A	-0.371664619	CC=C1CN2CCC34c5cc(ccc5N(C)C45C2CC1C3(CO5)C(=O)OC)O
N468	A	-0.374748346	C1Oc2cc3ccnc4C(c5ccccc5c(c2O1)c34)=O
N470	A	-0.380211242	CC(=C)C(CC=C(C)C)Cc1c(cc(c2C(C=C(c3ccc(cc3OC)O)Oc12)=O)OC)O
N469	A	-0.380211242	CC(=C)C(CC=C(C)C)Cc1c(cc(c2C(CC(c3ccc(cc3OC)O)Oc12)=O)OC)O
N472	A	-0.380211242	CC(C1CCC2C3CCC4CC(CCC4(C)C3CCC12C)N(C)C)N(C)C
N471	A	-0.380211242	COc1cc(c(C(C=Cc2ccccc2)=O)c(c1OC)O)O
N473	A	-0.397482747	[H]C(C1CCC(COC(C)=O)=C[C@H]2C(C(=C)C(=O)O2)[C@H](CC=1)O)=O
N475	A	-0.397940009	CC1C=C(C2C(C=CC=C2O)C=1c1c(cc(c2c1C[C@H](C)N[C@H]2C)OC)O)OC
N476	A	-0.400158583	CC(C)c1c2CC[C@H]3C(C)(C)CCC[C@]3(C)c2cc(c1O)O
N477	A	-0.403120521	CCC=C(C)CCC=C(C)c1c(cc2C(=C3c4cc(C)cc(c4C(c4c3cc(c(C(C)=CCCC(C)=CCC)c4O)O)=O)O)c3cc(C)cc(c3C(c2c1O)=O)O)O
N478	A	-0.407173509	CC=C1CN2C3CC1C(CO)(C2Cc1c2ccccc2[nH]c13)C(=O)OC

N479	A	-0.407173509	CC1C2CN3CCc4c5ccccc5[nH]c4C3CC2C(=CO1)C(=O)OC
N480	A	-0.412170596	CC1C2CN3C=CC4C(=C3CC2C(=CO1)C(=O)OC)N=c1ccccc1=4
N481	A	-0.414973348	[H]c1c2c(C(c3c(CCC=C(C)C)ccc(c3N2C)O)=O)c(c(CCC=C(C)C)c1O)O
N483	A	-0.414973348	CC(=C)C(CC=C(C)C)Cc1c(cc(c2C(C=C(c3ccc(cc3O)O)Oc12)=O)O)O
N482	A	-0.414973348	CC(=C)C(CC=C(C)C)Cc1c(cc(c2C(CC(c3ccc(cc3O)O)Oc12)=O)O)O
N484	A	-0.414973348	CN1C2C(=Cc3ccccc13)c1ccccc1N=2
N485	A	-0.422236551	Cc1c(cc2CCc3c(ccc(c3O)OC)C3CC(C)(C)Oc1c23)O
N486	A	-0.42434997	CC=C(C)C(=O)OC1(C)C(CC2C(C3C4(C)C=CC13OO4)OC(C2=C)=O)O
N488	A	-0.431363764	CC(C)=CCc1c(C)cc(c2c(c3C(=CC(C(CC=C(C)C)(CC=C(C)C)c3cc12)=O)O)O)O
N487	A	-0.431363764	CC(C)=CCOc1c2C=CC(=O)Oc2cc2c1cco2
N489	A	-0.444044796	CN1CCc2cc3c(cc2C(Cc2ccc4c(c2C1)OCO4)=O)OCO3
N490	A	-0.445846291	CC=C1CN2CCC34C2CC1C(=C3Nc1ccccc14)C(=O)OC
N491	A	-0.462397998	[H]C(C1C[C@@H](CC(C(C)CC)=O)[C@H]2C(=C)C(=O)O[C@H]2C=C(CCC=1)COC(C)=O)=O
N492	A	-0.462397998	[H]C1C([H])C(C)(C)C2(C(C(C3([H])C(=C)c4ccoc4CC3([H])C2(C)C1=O)OC(C)=O)OC(C)=O)O
N493	A	-0.462397998	CC(=O)OC(C)(C)C=CC(C(C)(C1C(CC2(C)C3CC=C4C(CC(C(C4(C)C)=O)O)C3(C)C(CC12C)=O)O)O)=O
N494	A	-0.46239799	CC(=O)OC(C)(C)C=CC(C(C)(C1C(CC2(C)C3CC=C4C(CC(C(C4(C)C)=O)O)C3(C)C(CC12C)=O)O)O)=O

		8	
N495	A	-0.46834733	CC(C)=CCCC(C)=CCc1c(cc2C(C3c4cc(C)cc(c4C(c4c3cc(cc4O)OCC=C(C)C)=O)O)c3cc(C)cc(c3C(c2c1O)=O)O)O
N496	A	-0.471291711	CC(c1cc2C(c3ccc(c(c3C(c2o1)=O)OC)OC)=O)=O
N500	A	-0.477121255	[H][C@@]1(C(=C)C(C)=O)[C@@H]2[C@H](C(=C)C(=O)O2)[C@H](C[C@@]1(COC(C)=O)C=C)OC(C(=C)CO)=O
N498	A	-0.477121255	[H][C@@]1(CCCCCCCCCCCCCCC)C[C@@](C)(CC(=O)OC)OO1
N499	A	-0.477121255	[H][C@@]12C(=C)C(=O)OC[C@]2(C[C@@H]([C@@H](C(=C)C(=O)OC)[C@@H]1O)OC(C(=C)CO)=O)C=C
N503	A	-0.477121255	C=CC12CC(C3C(C2C(=C)C(=O)OC1)OC(C3=C)=O)OC(C(=C)CO)=O
N505	A	-0.477121255	CC(=O)OCC1C[C@@H]([C@H]2C(=C)C(=O)O[C@@H]2C=C(CCC=1)C=O)OC(C(=C)CO)=O
N506	A	-0.477121255	CC(C(=C)C1[C@@H]2C(C(=C)C(=O)O2)[C@H](C[C@@]1(COC(C)=O)C=C)OC(C(=C)CO)=O)=O
N513	A	-0.477121255	CC(C)=CCc1c2c(c(c3c1c(c1ccoc1n3)OC)O)OCO2
N514	A	-0.477121255	CC(C)=CCc1c2c(c(c3c1c(c1ccoc1n3)OC)O)OCO2
N508	A	-0.477121255	CC(C)=CCCC(C)=CCc1c(cc(c2C(c3c(ccc(c3Oc12)O)O)=O)O)O
N504	A	-0.477121255	CC(C)=CCCC(C)=CCc1c(cc2c(C(c3c(ccc(c3O2)O)O)=O)c1O)O
N509	A	-0.477121255	CC(C)=CCCC1(C)C=Cc2c(cc3c(C(c4c(ccc(c4O3)O)O)=O)c2O)O1
N507	A	-0.477121255	CC(C1CCC=C(C[C@@H]([C@H]2C(=C)C(=O)O[C@@H]2C=1)OC(C(=C)CO)=O)COC(C)=O)=O
N501	A	-0.477121255	CC1(C)[C@H](Cc2c(cc(c3C(c4c(ccc(c4Oc23)O)O)=O)O)OC)[C@@]2(C)CC[C@@H]1O2

N510	A	-0.477121255	CC12CCCc3coc(C(c4cc5C(C=CC(c5cc14)=O)=O)=O)c23
N502	A	-0.477121255	Cn1ccc2cc3c(cc2c1Cc1ccc(cc1)O)OCO3
N511	A	-0.477121255	COc1cc2c(c3ccoc3nc2cc1OC)OC
N515	A	-0.482346027	CC(=O)O[C@H]1[C@H]2c3cc4c(cc3CN3CCC(=C[C@@H]1O)[C@H]23)OCO4
N516	A	-0.488550717	COc1cc2CCNC3Cc4ccc(c(c4c(c23)c1O)OC)OC
N518	A	-0.491361694	[H]C1C([H])C(C)(C)[C@@]2(C([H])[C@@H](C3([H])C(c4ccoc4C[C@]3([H])[C@@]2(C)C1=C([H])OC(C)=O)=O)OC(C)=O)O
N520	A	-0.491361694	CC(C)=CC(c1c(cc2c(c1C)OC(c1c(C)cc(c(C=O)c1O2)O)=O)OC)=O
N519	A	-0.491361694	CC1(C)C2CC(C3(C)C(CCC4(C)C(c5ccoc5)OC(C5C34O5)=O)C2(C)C=CC1=O)O
N521	A	-0.491361694	COC1C(C(CC23CCNC(C(c4ccc(c(c34)O)OC)O)C=12)O)O
N522	A	-0.505149978	CC(C)=CCCC1(C)C(CC=C(C)C)CC2(CC=C(C)C)C(C(=C(c3ccc(c(c3)O)O)O)C(C1(CC=C(C)C)C2=O)=O)=O
N523	A	-0.505149978	COc1cc(cc(c1OC)OC)OC1C(C(C(C(COC(c2cc(c(c(c2)O)O)O)=O)O1)O)O)O
N524	A	-0.509886892	CC(C)=CCCC(C)=CCc1c2C(c3c(cc(c(CC=C(C)CO)c3O)O)Oc2cc(c1OC)O)=O
N525	A	-0.51054501	CC(C)=CCc1ccc2c(C(CC(c3ccc(cc3)O)O2)=O)c1O
N526	A	-0.516075037	CC[C@H](CCC(C)C1CCC2C3CC=C4CC(CC[C@]4(C)C3CC[C@]12C)[C@H]1C(C([C@@H](C(CO)O1)O)O)O)C(C)C
N527	A	-0.516889046	CN1CCc2c3cc(c(c2Oc2cc4c(CCN(C)[C@H]4Cc4ccc(c(c4)Oc4ccc(CC13)cc4)O)c(c2OC)OC)OC)OC
N528	A	-0.51851394	C(CNC(c1cc(c[nH]1)[Br])=O)C=C1C(NC(N)=N1)=O

N529	A	-0.51851394	CC(=C)C(=O)OC1CC2(C)C(C=C(C(CO)=CC3C1C(=C)C(=O)O3)O2)=O
N530	A	-0.51851394	CC[C@@H]1[C@](C)([C@@H]([C@@H](C)N(C)C[C@H](C)C[C@](C)([C@@H]([C@@H](C)[C@@H]([C@@H](C)C(=O)O1)O[C@H]1C[C@](C)([C@H]([C@H](C)O1)O)OC)O[C@H]1[C@@H]([C@H](C[C@@H](C)O1)N(C)Cc1ccc(cc1)c1cn(CCCCCCCCCC(NO)=O)nn1)O)O)O)O
N531	A	-0.51851394	CC[C@@H]1C[C@]2(CC)[C@@H]3[C@H]1[C@@H](CC)OO[C@]3(CC)C(=O)O2
N532	A	-0.519344991	CC(C)c1c2CC[C@H]3C(C)(C)CCC[C@]3(C)c2cc(c1O)O
N533	A	-0.520672353	CC(C)c1cc2CCC3C(C)(C)CCCC3(C)c2cc1O
N534	A	-0.530199698	CC1C2CCC3C4CC=C5C[C@H](CCC5(C)C4CCC23CN1C)NC
N535	A	-0.531478917	C1=CN2C(c3c1c1ccccc1[nH]3)=Nc1ccccc1C2=O
N536	A	-0.531478917	C1CNC(c2c(C1=C1C(NC(N)N1)=O)c(c[nH]2)[Br])=O
N537	A	-0.531478917	CC(=O)O[C@H]1CCC(C)(C)[C@@]2([C@H]([C@@H]([C@H]3C(=C)c4ccoc4C[C@@H]3[C@@]12C)OC(C)=O)OC(C)=O)O
N538	A	-0.531478917	CC=C(C)C(NC1CCC2(C)C3CCC4(C)C(CCC4C(C)NC)C3CCC2C1OC(C)=O)=O
N540	A	-0.531478917	CC1(C)C=Cc2c(cc3c(C(C(=C(c4ccc(cc4)O)O3)OC)=O)c2O)O1
N539	A	-0.531478917	CC1(C)C=Cc2c(ccc(C(C=Cc3ccc(cc3)O)=O)c2O)O1
N541	A	-0.542948749	CC(C)c1cc2CC[C@H]3C(C)(C)CCC[C@]3(C)c2cc1O
N546	A	-0.544068044	CC(=C)C(=O)O[C@H]1C[C@]23C(O)OCC(CC[C@H]2O3)=C[C@@H]2[C@@H]1C(=C)C(=O)O2
N547	A	-0.544068044	CC(C)=CCc1c2c(C=CC(C)(C)O2)c(c2C(c3cc(c(c(c3Oc12)O)OC)OC)=O)O

N543	A	-0.544068044	CC(C1CCC2C3CCC4C(C(=CCC4(C)C3CCC12C)NC=O)=O)N(C)C
N544	A	-0.544068044	CCCCCCCCCCCCCCCCOC1C(C(C(C(COC(C)=O)O1)O)O)OC1C(C(C(C(C)O1)OC(C)=O)OC(C)=O)OC(C)=O
N545	A	-0.544068044	COc1ccc(cc1)C1=CC(c2c(cc(c(c3cc(ccc3OC)C3=CC(c4c(cc(cc4O3)O)O)=O)c2O1)O)O)=O
N548	A	-0.548178915	CC1(C)C(CC[C@@]2(C)[C@H]1CC[C@]1(C)[C@@H]2CC[C@@H]2[C@H]3[C@@H](CC[C@]3(C)CC[C@@]12C)C(=C)C=O)=O
N550	A	-0.556302501	[H]C(C(=C)[C@@H]1CC[C@]2(C)CC[C@]3(C)[C@]([H])(CC[C@]4([H])[C@@]5(C)CCC(C(C)(C)[C@]5([H])CC[C@@]34C)(O)O)[C@@]12[H])=O
N551	A	-0.556302501	CC12CCCC3(C=[N+](CCO)C1)C2CCC12CCC(CC13)C(=C)C2O.[Cl-]
N552	A	-0.556302501	CCCC(=O)OC1CC2C(C)(CC=C(C)C=C)C(C)C(C(C23C(OC(C3=C1)OC(C)=O)OC(C)=O)O)OC(C=Cc1ccc(cc1)O)=O
N554	A	-0.556302501	COc1cc(cc(c1O)OC)C1=CC(c2c(cc(c(c2OC)OC)OC)O1)=O
N553	A	-0.556302501	COc1ccc2c(CC3c4c(CCN3)c(c3c(c24)OCO3)OC)c1OC
N555	A	-0.562820799	CC(C)=CCCC(C)=CCc1c(cc2c(C(c3cc(c(cc3O2)O)OC)=O)c1O)O
N556	A	-0.565857046	CC=C1CC2C3CC1C(CO)N2Cc1c2ccccc2n(C)c13
N557	A	-0.568201724	[H][C@@]1(C)C[C@@H](C2C(C[C@@]3(C)CC[C@]1(O)O3)OC(C=2COC(C)=O)=O)OC(C(=C)CO)=O
N558	A	-0.568201724	[H][C@]12CCC3=C(CC[C@]4(C)[C@]3(C)CC[C@@]3(C)CC[C@@](C)(C[C@]34[H])C(O)=O)[C@@]2(C)CCC(C1(C)C)=O
N559	A	-0.568201724	[H]C1=C(C([H])(CC=C(C)C)c2cc3c(CC=C(C)C)c(C)c(CC=C(C)C)c(c3c(c2C1=O)O)O)O
N563	A	-0.568201724	CC(C)=CCc1c(cc(c2C(c3c(Cc12)c(CC=C(C)C)c(C)c(CC=C(C)C)c3O)=O)O)O
N560	A	-	CC(C)=CCCC(C)=CCCC(C)=CCCC(C)=CCO

		0.568201724	
N561	A	-0.568201724	CC(C)CNC(C=CC=CCCCCCCC=Cc1ccc2c(c1)OCO2)=O
N562	A	-0.568201724	CC1(C)C=Cc2c(cc(c3C(C4CC5c6c(C=4Oc23)c(cc(c6OC5(C)C)O)O)=O)O)O1
N564	A	-0.568201724	CCOC(C1=C2C(=c3ccccc3=N2)N(C)c2ccccc12)=O
N565	A	-0.571676282	CC1C(CC2C=1C1(C)C(CC(=O)OC)C3(C)C4C(C1O2)OC(C4(C)C=CC3=O)=O)c1ccoc1
N566	A	-0.574575378	CC(C)C1CCC2(C)CC3C(=CC(C12OO3)O)C=O
N567	A	-0.57863921	CC(=O)OCCCCCCCCCCC(C#CC#CC(C=C)OC(C)=O)O
N568	A	-0.579783597	CC(=O)OC1CCC(C)(C)C2(C(C(C3C(Cc4c(cco4)C3=C)C12C)O)OC(C)=O)O
N569	A	-0.584599383	COC1=C(C(C(=CC1=O)C1Cc2cc(c(c(c2OC1)OC)OC)OC)=O)OC
N570	A	-0.584599383	COC1=C(C(C(=CC1=O)C1Cc2cc(c(c(c2OC1)OC)OC)OC)=O)OC
N571	A	-0.591064607	[H][C@@]1(C)C[C@@H](C2C(C[C@@]3(C)CC[C@]1(O)O3)OC(C=2COC(C)=O)=O)OC(C(C)=CC)=O
N572	A	-0.591064607	C[C@]12CCC(c3coc(C(c4cc5C(C6=C(C(c5cc14)=O)S(CCN6)(=O)=O)=O)=O)c23)=O
N573	A	-0.591064607	CC(C)=CCc1c(c(CC=C(C)C)c2c(C(CC(c3ccc(cc3)O)O2)=O)c1O)O
N574	A	-0.591064607	CC(C)=CCc1c(c(CC=C(C)C)c2c(C(CC(c3ccc(cc3)O)O2)=O)c1O)O
N575	A	-0.591064607	CC(C)=CCc1c(c2C(c3cc(c(c(c3Oc2c2C=CC(C)(C)Oc12)O)OC)OC)=O)O
N576	A	-0.593728427	CC(C)=CCCC(C)=CCCC(C)=CCCC1(C)C(CCCO)C(CCC1(C)O)=C(C)C=O

N577	A	-0.599883072	COc1cc(c2C(C=C(c3ccccc3)Oc2c1)=O)O
N578	A	-0.602059991	[H]C12Cc3ccc(c(c3c3c1c(CCN2([H])[H])cc(c3OC)OC)O)OC
N579	A	-0.602059991	[H]CC1(C)C2=C[C@@H]([C@]3([H])[C@@](C)(C[C@H]([C@]4(C)[C@H](CC[C@@]34C)[C@H](C)CC=CC(C)(C)O)O)[C@]2([H])CC[C@@H]1O)O
N584	A	-0.602059991	CC(=O)OC1CC(C2(C)c3cc4c(cco4)c(C)c3CCC2(C1(C)C)O)OC(C)=O
N581	A	-0.602059991	CC(=O)OC1CC2C(C)(C=CC(=O)OC2(C)C)C2CCC3(C)C(c4ccoc4)OC(C4C3(C12C)O4)=O
N580	A	-0.602059991	CC1(C)C2=CC[C@H]3[C@]4(C)C[C@H]([C@@H]([C@@]4(C)CC([C@@]3(C)[C@@H]2C[C@@H](C1=O)O)=O)[C@](C)(C(C=CC(C)(C)O)=O)O)O
N582	A	-0.602059991	Cc1c2C(c3c(ccc(c3O)C3(c4cccc(c4C(c4c3cc(c(C(=O)OC)c4C)O)=O)O)O)C(c2cc(c1C(=O)OC)O)=O)=O
N583	A	-0.602059991	Cc1c2C(c3c(cccc3O)C(c2cc(c1C(=O)OC)O)=O)=O
N585	A	-0.602059991	Cc1cc2C(c3cc(cc(c3C(c2c(c1)O)=O)O)O)=O
N586	A	-0.602059991	CCCCCCCCCCCCCCCC[C@]1(C)C[C@@](C)(CC(=O)OC)OO1
N588	A	-0.612783857	[H]C([H])=C1[C@@]23C([H])([H])C(=O)O[C@@]([H])(C4=C([H])[C@]([H])(O[H])OC4=O)[C@]2(C([H])([H])[H])C([H])([H])C([H])([H])[C@]1([H])[C@@]1(C([H])([H])[H])[C@]([H])(C([H])([H])C(C(C([H])([H])[H])(C([H])([H])[H])[C@]1([H])[C@@]([H])(C(=O)OC([H])([H])[H])OC(C([H])([H])[H])=O)=O)O3
N589	A	-0.612783857	CC(C)=CCc1c(cc2c(C(c3c(cc(c(c3CC=C(C)C)OC)O)O2)=O)c1O)O
N590	A	-0.612783857	CC=CC(c1c(cc2cc(cc(c2c1OC)OC)OC)OC)=O
N591	A	-0.612783857	CC1(C)C2CC(C3(C)C(CCC4(C)C(c5ccoc5)OC(C5C34O5)=O)C2(C)C=CC1=O)=O
N592	A	-	CCCC[C@@H](CCCCCCCC[C@H](CC(O)=O)O)OC

		0.612783857	1[C@@H]([C@H]([C@@H]([C@@H](CO[C@@H]2[C@H]([C@H]([C@@H](C(C)O2)O)O)O)O1)O)O)O[C@@H]1C([C@@H]([C@H]([C@@H](CO)O1)O)O)OC1[C@@H]([C@@H]([C@@H](C(C)O1)O)O)O
N593	A	-0.620136055	CC(C)C1CC2C(C)(CC=1)C(CCC2(C)O)O
N596	A	-0.62324929	CC(=O)OC1[C@H](C2C(C)(C)C(C=C[C@]2(C)C2CC[C@]3(C)C(CC=C3[C@]12C)c1ccoc1)=O)OC(C)=O
N594	A	-0.62324929	CC=C(C)C(=O)O[C@H]1[C@H]2C(=C)C(=O)O[C@@H]1[C@]1(C)[C@H](CC[C@@](C)([C@H]1[C@H]2OC(C(C)C)=O)O)O
N595	A	-0.62324929	COc1cc2C3Cc4cc(c(cc4C[N+]3(CCc2cc1O)[O-])OC)OC
N597	A	-0.632457292	CN1CCc2cc3c(cc2C(Cc2ccc4c(c2C1)OCO4)=O)OCO3
N598	A	-0.632457292	COc1cc2CCN3Cc4c(CC3c2cc1O)ccc1c4OCO1
N599	A	-0.632457292	COc1cc2CCN3Cc4c(CC3c2cc1O)ccc1c4OCO1
N600	A	-0.633468456	CC(CCCCC#C)CC(C)C(N(C)C(Cc1ccccc1)C(NC(C)C(N(C)C(C)C(N(C)C(Cc1ccc(cc1)OC)C(N)=O)=O)=O)=O)=O
N601	A	-0.633468456	CC1(C)[C@H](CC[C@]2(C)C3CC[C@@]4(C)[C@@H]5C[C@](C)(CC[C@]5(C)CC[C@]4(C)C=3CC[C@@H]12)C(O)=O)O
N602	A	-0.643452677	CC(=C)C(=O)OC1CC2(C)C(C=C(C(=C)C(C3C1C(=C)C(=O)O3)O)O2)=O
N603	A	-0.643452677	CC1C2=CC([C@@](C)(C[C@@H]([C@H]3C(=C)C(=O)O[C@@H]3[C@H]1O)OC(C(C)=C)=O)O2)=O
N604	A	-0.643452677	CC1c2c(cc(c(c3ccc(c4c(cc(C)cc34)OC)OC)c2C[C@H](C)N=1)O)OC
N605	A	-0.643452677	CC1c2c(cc(c(c3ccc(c4c(cc(C)cc34)OC)OC)c2C[C@H](C)N1C)OC)OC
N606	A	-0.64738297	CC(C)C(=O)O[C@H]1C[C@@H]2[C@@](C)(CC=C(C)C=C)[C@@H](C)C[C@H]([C@]23C(=C1)[C@@H](OC(C)=O)O[C@H]3OC(C)=O)OC(c1ccccc1)=O
N607	A	-0.65062037	CC(=C)C(=O)O[C@H]1C[C@H](C)C2=CC([C@](C)(C=C3C1=C(COC(C)=O)C(=O)O3)O2)=O

N608	A	-0.651278014	CC=C1CN2CCC34C2CC1C1=CN2C5C(=CN(C13)c1ccccc14)C1CC3C5(CCN3CC1=CC)c1ccccc12
N609	A	-0.651470745	[H][C@]12Cc3ccc(cc3)Oc3cc(C[C@@]4([H])c5c(CCN4C)cc(c(c5Oc4c(CCN2C)c1cc(c4OC)OC)OC)O)ccc3O
N614	A	-0.653212514	[H][C@]12CC[C@@]3([H])[C@@H](C(=O)OCCCCCCCCC[C@@H]4C(=C5CCCN5C(=N)N4)C(=O)OCCCCNC(N)=N)[C@@H](C)NC(N[C@H](CCCCCCCCC)C1)=N23
N610	A	-0.653212514	[H][C@]12CC[C@]3([H])C[C@H](C)NC(=NC(CCCCCCC(C)OC([C@H]4[C@@H](C)N=C5N[C@H](CCCCCCCC)C[C@]6([H])CN[C@]4([H])N56)=O)C1)N23
N611	A	-0.653212514	[H][C@]12CC[C@]3([H])C[C@H](C)NC(=NC(CCCCCCC(C)OC([C@H]4[C@@H](C)N=C5N[C@H](CCCCCCCCC)C[C@]6([H])CN[C@]4([H])N56)=O)C1)N23
N613	A	-0.653212514	[H][C@]12CCc3c(C(=O)OCCCCNC(N)=N)c(CCCCC)nc(NC(CCCCCCCCC)C1)n23
N612	A	-0.653212514	[H][C@]12CCc3c(C(O)=O)c(C)nc(N[C@H](CCCCCCC)C1)n23
N616	A	-0.653212514	C[C@H]1Cc2c(c3ccc(c4c(cccc34)OC)OC)c(cc(c2[C@@H](C)N1C)OC)OC
N615	A	-0.653212514	CC(=C)C(=O)O[C@H]1C[C@H](C)[C@@]2(CC[C@](C)(CC3C1=C(COC(C)=O)C(=O)O3)O2)O
N617	A	-0.653212514	CC(C)=CCC1=C(c2ccc(cc2O)O)Oc2c3C=CC(C)(C)Oc3cc(c2C1=O)O
N621	A	-0.653212514	CC[C@H]1C=CCC[C@@]2(C[C@@H]3CC[C@@H]4[C@H](C(=O)OCCCCCCCCCCCCCCCCC(N(CCCCN)CCCN)=O)[C@]5(CCC[C@@H](C)O5)N=C(N2)N34)O1
N618	A	-0.653212514	CCCCCCC[C@@H]1C[C@@H]2CC[C@H]3[C@@H](C(=O)OCCCCCCCCC[C@@H]4C(=C5CCCN5C(=N)N4)C(=O)OCCCCNC(N)=N)[C@@H](C)N=C(N1)N23
N619	A	-0.653212514	CCCCCCCC[C@@H]1C[C@@H]2CC[C@H]3[C@@H](C(=O)OCCCCCCCCC[C@@H]4C(=C5CCCN5C(=N)N4)C(=O)OCCCCNC(N)=N)[C@@H](C)N=C(N1)N23
N622	A	-0.653212514	CCCCCCCCCCCCCCCCCCCC(=O)OC(COC(CCCCCCCCCCCCCCC)=O)CO[C@H]1[C@@H]([C@H]([C@@H]([C@@H](CN)O1)O)O)O

N620	A	-0.653212514	COC1C(C(CC23CCNC(Cc4ccc(c(c34)O)OC)C=12)O)O
N623	A	-0.656098202	CC1CCC(C2(C)CCC(CC12O)C(C)=C)=O
N625	A	-0.662757832	[H]C12C(C(C=O)C3=C(C(C(=C(C(C)C)C3=O)O)=O)C2(C)CCCC1(C)C)O
N626	A	-0.662757832	CC(=O)OC1C(Cc2ccc(cc2)OC)NCC1O
N627	A	-0.662757832	CC1C(C(C(C(O1)OC1C(c2ccc(c(c2)O)O)Oc2cc(cc(c2C1=O)O)O)O)OC(C=Cc1ccccc1)=O)O
N628	A	-0.667072309	CC(C)=CCc1c(c2C(C3CC4c5c(C=3Oc2c2C=CC(C)(C)Oc12)c(cc(c5OC4(C)C)O)O)=O)O
N629	A	-0.668385917	[H][C@@]12C[C@@H](C=C3C(OC([C@@]13[C@H](C[C@@H](C)[C@@]2(C)CC=C(C)C=C)OC(c1ccccc1)=O)OC(C)=O)OC(C)=O)OC(C(C)CC)=O
N631	A	-0.672097858	CC(C)C1=CC(c2c(cc3c(CCCC3(C)C)c2C)C1=O)=O
N632	A	-0.672097858	CC(C)C1=CC(c2c(cc3c(CCCC3(C)C)c2C)C1=O)=O
N630	A	-0.672097858	CC(C)C1=CC2=CC=C3C(C)(C)CC(CC(C)c4ccc(c(c4)O)O)CC3(C)C2=C(C1=O)O
N633	A	-0.672097858	CC1=CC2=CC(C=C3C2=C(C=C(C)O3)O1)=O
N634	A	-0.672097858	CCCCCCC=CCCCCCCCC(C[C@]1(C[C@@H](C=CC1=O)O)O)O
N635	A	-0.672097858	CCCCCCC=CCCCCCCCC1C[C@]2(C[C@@H](C=CC2(O)O1)O)O
N636	A	-0.672097858	CCCCCCCCCCCCCCCCOC1C(C(C(C(COC(C)=O)O1)O)OC(C)=O)OC1C(C(C(C(C)O1)OC(C)=O)OC(C)=O)OC(C)=O
N637	A	-0.674244837	CC(C)C1=CC(c2c(cc3c(CCCC3(C)C)c2C)C1=O)=O
N638	A	-	CC1Cc2c(C(C)=N1)c(cc(c2c1c(C)cc(c2c(cccc12)OC)

		0.678284821	OC)OC)OC
N639	A	-0.681241237	CC(C)=CCc1c(c2C(C3CC4c5c(C=3Oc2c2C=CC(C)(C)Oc12)c(cc(c5OC4(C)C)O)O)=O)O
N640	A	-0.681241237	Cc1cc(c2c(cccc2c1c1c(cc2C[C@H](C)N[C@H](C)c2c1OC)O)OC)OC
N641	A	-0.683947131	CC(=C)C(=O)OC1CC2C(C)(C(C3C4C(C)(CO3)C=CC(C24C)=O)O)C2=CCC(c3ccoc3)C12C
N642	A	-0.688419822	CC(C)=CCC1=C(c2ccccc2C(C1=O)=O)O
N643	A	-0.69019608	[H]C12C=C(C)C(C(CC(C(C)(C)CC=C(C)C(C2(C(C(C)C1OC(c1ccccc1)=O)OC(C)=O)OC(C)=O)OC(C)=O)OC(C)=O)OC(C)=O)=O
N644	A	-0.69019608	CC(C)c1cc2CCC3C(C)(C)CCCC3(C)c2cc1O
N645	A	-0.69019608	CN1Cc2cc(c(cc2c2ccc3cc4c(cc3c12)OCO4)OC)OC
N646	A	-0.690900187	CC1Cc2c(c(cc(c2C(C)N1)OC)O)c1c(C)cc(c2c(cccc12)OC)OC
N647	A	-0.691277242	CCC1CC2CC3(C1N(CCc1c4cc(ccc4[nH]c13)OC)C2)C(=O)OC
N648	A	-0.69292509	CCCCCc1cc2c(c3cc(C)ccc3C(C)(C)O2)c(c1C(=C)CC1(CCC(C)=CC1)C(C)C)O
N649	A	-0.696356389	CC1(C)CCC[C@@]2(C)C1CC[C@@]1(CO1)[C@@H]2CC=C(C1CO1)C(=O)OC
N650	A	-0.696356389	CC1(C)CCC[C@@]2(C)C1CC[C@@]1(CO1)[C@@H]2CC=C(C1CO1)C(=O)OC
N657	A	-0.698970004	CC(=O)OC1CC(C)(C)C2(CCc3c(cc(c(C=O)c3C)O)C2(C)C1OC(C)=O)O
N653	A	-0.698970004	CC(=O)OCC[C@H]1C[C@H]2c3c(CCN2C[C@@H]1C=C)c1cc(ccc1[nH]3)O
N654	A	-0.698970004	CC(C)=CC[C@@H]1C[C@]23C[C@H](C=CC(C)(C)O)C(C)(C)OC2=C(C(c2ccc(c(c2)O)O)=O)C([C@@](CC=C(C)C)(C3=O)C1(C)C)=O
N655	A	-0.69897000	CC(C)=CC[C@@H]1C[C@]23C[C@H](CCC(C)(C)O)C(C)(C)OC2=C(C(c2ccc(c(c2)O)O)=O)C([C@@](CC

		4	=C(C)C)(C3=O)C1(C)C)=O
N652	A	-0.698970004	CC(C)=CC[C@@H]1C[C@]23CC4CCC(C)(C)C[C@@]4(C)OC2=C(C(c2ccc(c(c2)O)O)=O)C([C@@](CC=C(C)C)(C3=O)C1(C)C)=O
N656	A	-0.698970004	CC(C)=CC[C@H]1C[C@@]23C[C@@H](CC(C(C)(C)O)O)C(C)(C)[C@@](CC=C(C)C)(C(C(=C2OC1(C)C)C(c1ccc(c(c1)O)O)=O)=O)C3=O
N651	A	-0.698970004	CC(C)=CC[C@H]1C[C@@]23C[C@@H](CC=C(C)C)C(C)(C)[C@@](CC=C(C)C)(C(C(=C2OC1(C)C)C(c1ccc(c(c1)O)O)=O)=O)C3=O
N658	A	-0.698970004	CC(C)=CCc1c(C)cc2cc3c(C(=CC(C3(CC=C(C)C)CC=C(C)C)=O)O)c(c2c1O)O
N662	A	-0.698970004	Cc1cc(c2c(ccc(c3c(cc4C[C@H](C)N(C)[C@H](C)c4c3OC)O)c2c1)OC)OC
N659	A	-0.698970004	CC1CC(CCCC(C=C(C)C(C)O)O)(OC)OOC1CC(=O)OC
N660	A	-0.698970004	CC1CC(CCCC=CC(C)=O)(OC)OOC1CC(=O)OC
N661	A	-0.698970004	CCC(C)C(=O)OC1(c2cccc(c2C(c2c1cc(c(C(=O)OC)c2C)O)=O)O)c1ccc2c(C(c3c(cc(c(C(=O)OC)c3C)O)C2(c2ccc3C(c4cc(c(C(=O)OC)c(C)c4C(c3c2O)=O)O)=O)OC(C(C)CC)=O)=O)c1O
N663	A	-0.704150517	CC12COC3C2C(C)(C2CCC4(C)C(CC=C4C2(C)C3O)C2=CCOC2=O)C(C=C1)=O
N664	A	-0.704922639	[H][C@]12C=C(C)C([C@@H]([C@@H](C(C)(C)CC=C(C)[C@H]([C@]2([C@H]([C@H](C)[C@@H]1Oc1ccccc1)OC(C)=O)OC(C)=O)OC(C)=O)OC(C)=O)OC(C)=O)=O
N665	A	-0.706112306	Cc1cc(c2c(ccc(c3c(cc(c4c3C[C@@H](C)N[C@@H]4C)OC)O)c2c1)OC)O
N666	A	-0.707570176	[H]C1(C(=C)C(CC)=O)[C@@]2(C)C=C(C)C[C@]1(C)C=C(C)[C@@H]2C(C)=C[C@H]1CC(C=C1)=O
N670	A	-0.707570176	CC(C)C=Cc1cc(C=CC(c2cc(C=CC(C)C)c(cc2O)O)=O)ccc1O
N667	A	-0.707570176	Cc1cc2C(C3C(C(C(C(O3)OC(c3ccccc3)=O)O)O)O)c3cccc(c3C(c2c(c1)O)=O)O
N668	A	-	Cc1cc2C(C3C(C(C(C(O3)OC(c3ccccc3)=O)O)O)O)c

		0.707570176	3cccc(c3C(c2c(c1)O)=O)O
N669	A	-0.707570176	CCCCCCCCCC=CC(N[C@H]1C[C@]2(C(C=C[C@H]([C@@H]2O[C@H]1O)[Cl])=O)O)=O
N674	A	-0.716003344	CC(=O)O[C@H]1[C@H]2[C@H]3[C@H](C(=O)O2)c2ccoc2C[C@@H]3[C@@]2(C)[C@H](CCC(C)(C)[C@]12O)O
N672	A	-0.716003344	CC(C)=CCC1=C(c2ccc(cc2O)O)Oc2cc3c(C=CC(C)(C)O3)c(c2C1=O)O
N671	A	-0.716003344	CC(C)=CCc1c(cc(c(c1CC=C(C)C)O)O)C1CC(c2c(cc(cc2O1)O)O)=O
N673	A	-0.716003344	CC(C)CC(=O)OC1CC2C(C)(CC=C(C)C=C)C(C)C(C(C23C(OC(C3=C1)OC(C)=O)OC(C)=O)O)OC(C)=O
N675	A	-0.717988466	CC(C)=CCc1c(cc2c(C(c3c(CC=C(C)C)c(c(c(CC=C(C)C)c3O2)O)OC)=O)c1O)O
N676	A	-0.72427587	CC(C)=CCc1cc(cc(CC=C(C)C)c1O)C1CC(c2c(cc(c(CC=C(C)C)c2O1)O)O)=O
N677	A	-0.72427587	CC(C)C1=CC2=CC=C3C(C)(C)CC(CC3(C)C2=C(C1=O)O)OC(c1ccc(cc1)O)=O
N678	A	-0.727541257	CCC=C(C)CCC=C(C)c1c(cc2C(c3cc(C)cc(c3C(c2c1O)=O)O)=O)O
N679	A	-0.73239376	C(C[C@@H]1c2c(CCN1)c1ccc(cc1[nH]2)[Br])CNC(N)=N
N18	A	-0.73239376	C(CC1c2c(CCN1)c1ccc(cc1[nH]2)[Br])CN=C(N)N
N681	A	-0.73239376	CC1c2c(cc(c(c3ccc(c4c(cc(C)cc34)OC)O)c2C[C@H](C)N=1)O)OC
N680	A	-0.73239376	CCCCCCC(CO)C=C(C)[C@H]([C@@H](C)C(=O)O[C@@H]1CC[C@H](C)[C@@]2(C)CC3C(=C[C@]12O)OC(C=3C)=O)O
N682	A	-0.734589152	CC(=O)OC1CC2C(C)(C)C(CCC2(C)C2CCC3(C)C(c4ccoc4)OC(C4C3(C12C)O4)=O)=O
N683	A	-0.735793924	CC(C)C=Cc1c(cc(C=Cc2ccc(cc2O)O)cc1O)O
N684	A	-0.738776306	CC(COC(C)=O)=CC(=O)O[C@H]1C[C@H](C)C2=CC([C@](C)(C=C3C1=C(COC(C)=O)C(=O)O3)O2)=O
N686	A	-	CC(C)CC(=O)OC1CC2C(C)(CCC(=C)C=C)C(C)CC(

		0.74036269	C23C(OC(C3=C1)OC(C)=O)OC(C)=O)OC
N685	A	-0.74036269	CC(CC(=O)O[C@H]1C[C@H](C)[C@@]2(CC[C@](C)(CC3C1=C(COC(C)=O)C(=O)O3)O2)O)COOC(C)=O
N687	A	-0.74036269	COc1cc(cc(c1O)O)C1C(CO)Oc2cc3c(C=CC(=O)O3)cc2O1
N688	A	-0.744754413	CCC1CC2CC3(C1N(CCc1c4cc(c(cc4[nH]c13)OC)OC)C2)C(=O)OC
N689	A	-0.747609272	CC1CCC=C2C(CC3C(C12C)C(C)(C)OO3)=O
N694	A	-0.748188027	CC(C)C1c2nc(cs2)C(NC(C)c2nc(C(NC(C(C)O)c3nc(cs3)C(N1)=O)=O)c(C)o2)=O
N695	A	-0.748188027	CC(C)C1c2nc(cs2)C(NC(C)c2nc(C(NC(C(C)O)c3nc(cs3)C(N1)=O)=O)c(C)o2)=O
N693	A	-0.748188027	CC(C)C1CCC(C)=CC1c1c(cc(c(C(CCc2ccccc2)=O)c1O)O)OC
N690	A	-0.748188027	CC(Cc1cc(cc2c1C(C=C(CC1(C)CC(c3c(CC(C)=O)cc(cc3O1)O)=O)O2)=O)O)=O
N691	A	-0.748188027	CC(Cc1cc(cc2c1C(C=C(CC1(C)CC(c3c(CC(C)=O)cc(cc3O1)O)=O)O2)=O)O)=O
N692	A	-0.748188027	CC=C(C)C(=O)OC1CC2(C)C(C=C(C(=C)C(C3C1C(=C)C(=O)O3)O)O2)=O
N696	A	-0.751279104	CC(C)C1CCC(C)=CC1c1c(cc(c(C(CCc2ccccc2)=O)c1O)O)OC
N697	A	-0.751279104	CC(C)C1CCC(C)=CC1c1c(cc(c(C(CCc2ccccc2)=O)c1O)O)OC
N698	A	-0.755874856	[H]C1C=C2C[C@H](CC[C@]2(C)C2C[C@H]([C@]3(C)[C@H](CC[C@@]3(C12)O)C(C)OC(C)c1ccccc1)OC(C)c1ccccc1)O[C@H]1[C@@H]([C@@H]([C@@H]([C@@H](CO)O1)O[C@H]1[C@@H]([C@@H]([C@@H]([C@@H](CO)O1)O[C@H]1[C@@H]([C@@H]([C@@H]([C@@H](CO)O1)O[C@H]1[C@@H]([C@@H]([C@@H]([C@@H](CO)O1)O)O)O)OC)O)OC)O)OC)O
N700	A	-0.755874856	CC(=O)O[C@@H]1[C@@H]([C@H]2[C@@](C)(C)C(C=C[C@]2(C)[C@H]2CC[C@]3(C)C(=CC([C@H]3c3ccoc3)=O)[C@]12C)=O)OC(C)=O

N699	A	-0.755874856	CC(CC1c2c(ccc(c2OC)OC)c2ccc3cc4c(cc3c2N1C)OCO4)=O
N701	A	-0.759883191	CC(=O)OC1CC2(C)C(c3ccoc3)OC(C3C2(C2(C)C1C1(C)C=CC(C(C)(C)C1CC2OC(C)=O)=O)O3)=O
N702	A	-0.762678564	CC1=CC(c2c(ccc(c2C1=O)O)O)=O
N703	A	-0.763427994	[H]C12C(C(C)=C(C(CC(C(C)(C)CC=C(C)C(C2(C(C(C)C1OC(c1ccccc1)=O)OC(C)=O)OC(C)=O)OC(C)=O)OC(C)=O)OC(C)=O)OC(C)=O)O
N704	A	-0.763427994	CC(=C)C1CCC2(CCC3(C)C(CCC4C5(C)C(C(C(C)(C)C5CCC34C)OC(c3ccc(c(c3)OC)O)=O)C(O)=O)C12)C(O)=O
N705	A	-0.763427994	CC(=O)OC1C2C(Cc3c(cco3)C2=C)C2(C)C(CCC(C)(C)C2(C1O)O)=O
N706	A	-0.763427994	CCC(C)C(C(NC(C(C)C)C(NC(C(C)C)C(N(C)C(C(C)C)C(=O)OC(C(C)CC)C(N1CCCC1C(=O)OC)=O)=O)=O)=O)N(C)C(CCCC(CCCC#C)OC)=O
N707	A	-0.763427994	COC1=CC(C(=CC1=O)C(C=C)c1ccccc1)=O
N708	A	-0.763427994	COC1=CC(C(=CC1=O)C(C=C)c1ccccc1)=O
N709	A	-0.763427994	COC1=CC(C2=C(CCc3ccccc3O2)C1=O)=O
N710	A	-0.770817478	CCC1C(=O)OC2(C)CC(C3C(C4C(C)(CCC12O)O4)OC(C=3COC(C)=O)=O)OC(C(=C)CO)=O
N711	A	-0.770852012	[H]C12C=C(C)C(C(C)CC(C(C)(C)C=CC(C)(C(C2(C(C(C)C1OC(c1ccccc1)=O)O)OC(C)=O)O)O)OC(C)=O)OC(c1ccccc1)=O
N712	A	-0.770852012	CCC(C)C(C(NC(C(C)C)C(N1CCC2C1C(N1CCCC1C(NC=Cc1cc(ccc1OC)O2)=O)=O)=O)=O)N(C)C
N713	A	-0.775974331	CC(C)(C=C)c1cc(C=CC(c2ccc(cc2)O)=O)c(cc1O)OC
N716	A	-0.77815125	[H][C@@]12C[C@@H](C=C3[C@@H](OC([C@@]23[C@@H](C[C@H](C)[C@]1(C)CC=C(C)C=C)O)OC(C)=O)OC(C)=O)OC(C(C)CC)=O
N715	A	-	[H][C@]12C[C@]3(C)[C@@H](C)CC[C@H]([C@@]3

		0.77815125	4[C@@H]([C@]2(O)OCC1=C)O4)OC([C@H](C)[C@@H](C(C)=CC(CCCCCC)CO)O)=O
N717	A	-0.77815125	[H]C12C(C(C)=C(C(CC(C(C)(C)CC=C(C)C(C2(C(C(C)C1OC(c1ccccc1)=O)O)OC(C)=O)OC(C)=O)OC(C)=O)OC(C)=O)OC(C)=O)O
N721	A	-0.77815125	CC(=O)OC(C1C(C)(C)C(C=CC1(C)C1CCC2(C)C(c3ccoc3)OC(CC2(C1=C)O)=O)=O)C(=O)OC
N730	A	-0.77815125	CC(C)=CCc1c(cc2c(C(c3c(cc(c(c3CC=C(C)C)OC)O)O2)=O)c1O)O
N722	A	-0.77815125	CC(C)=CCc1c(cc2c(C(c3c(CC=C(C)C)c(c(c(CC=C(C)C)c3O2)O)O)=O)c1O)O
N723	A	-0.77815125	CC(C)=CCc1c(cc2c(C(c3c4C=CC(C)(C)Oc4c(c(CC=C(C)C)c3O2)O)=O)c1O)O
N718	A	-0.77815125	CC(C)=CCc1c(cc2c(C(c3c4CC(C(C)(C)Oc4c(c(CC=C(C)C)c3O2)O)O)=O)c1O)O
N719	A	-0.77815125	CC(C)=CCc1c2C(c3c(cc4c(C=CC(C)(C)O4)c3O)Oc2c(CC=C(C)C)c(c1O)O)=O
N731	A	-0.77815125	CC(C)=CCc1c2C(c3c(cc4c(C=CC(C)(C)O4)c3O)Oc2cc(c1OC)O)=O
N732	A	-0.77815125	CC(C)=CCc1c2C(c3c(cc4c(CCC(C)(C)O4)c3O)Oc2cc(c1OC)O)=O
N720	A	-0.77815125	CC(C)=CCCC(C)=CCc1c(cc2c(C(c3c(cc(c(c3CC=C(C)C)OC)O)O2)=O)c1O)O
N729	A	-0.77815125	CC(C)=CCCC(C)=CCc1c2C(c3c(cc(cc3Oc2cc(c1OC)O)O)O)=O
N724	A	-0.77815125	CC(C)C1=CC2=CC=C3C(C)(CCCC3(C)C2=C(C1=O)O)COC(C(C)C)=O
N725	A	-0.77815125	CC(CCCCC#C)C(N(C)C(Cc1ccccc1)C(NC(C)C(N(C)C(C)C(N(C)C(Cc1ccc(cc1)OC)C(N)=O)=O)=O)=O)=O
N726	A	-0.77815125	CC(CCCCC#C)C(N(C)C(Cc1ccccc1)C(NC(C)C(N(C)C(C)C(N(C)C(Cc1ccc(cc1)OC)C(N)=O)=O)=O)=O)=O
N727	A	-0.77815125	CC=C(C)C=CCCCC1(CC(C)C(CC(=O)OC)OO1)OC
N728	A	-0.77815125	CC1(C)C2CC(C3(C)C(CCC4(C)C(c5ccoc5)OC(C5C34O5)=O)C2(C)C=CC1=O)=O
N734	A	-0.77815125	COc1ccc(cc1)[C@@H]1CC(c2c(cc(c(c2O1)[C@@H]1C(c2c(cc(cc2O[C@H]1c1ccc(cc1)O)O)O)=O)O)O)=O
N733	A	-0.77815125	COc1ccc(cc1)C1CC(c2c(cc(c(C3=C(c4ccc(cc4)O)Oc4cc(cc(c4C3=O)O)OC)c2O1)O)O)=O
N735	A	-0.781036939	CCC(C)C(=O)O[C@@H]1C[C@@H]2[C@](C=O)(C(=C1)C=O)[C@H](C[C@@H](C)[C@@]2(C)CC=C(C)C=C)OC
N736	A	-	CC(=O)OC(C1C(C)(C)C(C=CC1(C)C1CCC2(C)C(c3c

		0.782030428	coc3)OC(CC2(C1=C)O)=O)=O)C(=O)OC
N737	A	-0.785329835	CC(C)=CCc1cc(cc(CC=C(C)C)c1O)C1CC(c2c(cc(cc2O1)O)O)=O
N738	A	-0.785329835	CN1c2c(cccc2O)C(c2c1c(c(c(c2O)OC)OC)OC)=O
N740	A	-0.785329835	COc1cc(c2c(C=O)c(c3ccc(c(c3OC)O)OC)oc2c1)O
N739	A	-0.785329835	COc1ccc(cc1OC)C(CNC(C=Cc1ccccc1)=O)OC(C=Cc1ccccc1)=O
N741	A	-0.787298101	Cc1c(cc2CCc3c(ccc(c3O)O)C3CC(C)(C)Oc1c23)O
N742	A	-0.79239169	CC1c2c(cc(c(c3ccc(c4c(cc(C)cc34)O)OC)c2C[C@H](C)N1C)OC)OC
N743	A	-0.79716974	CC(C)=CCCC(C)=CCc1c2C(c3c(cc(c(CC=C(C)C)c3O)O)Oc2cc(c1OC)O)=O
N744	A	-0.797959644	[H][C@]12CC[C@H]([C@H](C)C=C[C@H](C)C(C)C)[C@@]2(C)CC[C@@]2([H])[C@]13C=C[C@]1(C[C@H](CC[C@@]12C)O)OO3
N745	A	-0.79934055	CC(C)=CC[C@]12C([C@]3(C[C@@H](C(C)(C)O2)[C@]12C(=C3)C(c1c(cccc1O2)O)=O)OC)=O
N748	A	-0.79934055	CC(C)=CCc1ccc(C2=COc3cc(cc(c3C2=O)O)O)c(c1OC)O
N746	A	-0.79934055	CC(C)=CCCC(C)=CCc1c2C(c3c(cc(c(CC=C(C)C)c3O)O)Oc2cc(c1O)O)=O
N747	A	-0.79934055	CC1C2C(NCc3nc(cs3)C(NC(CCSC)C3=NC(CS3)C(NC(Cc3ccccc3)C(=N2)O1)=O)=O)=O
N752	A	-0.806179974	C[C@H]1CC[C@@]2(C)[C@@H](CC=C3C(=O)O[C@H]([C@]23O)O)C1=C
N750	A	-0.806179974	CC(=O)OC1CC2C(C)(C)C(C=CC2(C)C2CCC3(C)C(C(C=C3C12C)=O)c1ccoc1)=O
N749	A	-0.806179974	CC(C)=CCC1=C(c2cc(c(cc2O)O)O)Oc2c3C=CC(C)(C)Oc3cc(c2C1=O)O
N751	A	-0.806179974	CCC(C)C(C(NC(CC(C)C)C(N1CCC2C1C(N1CCCC1C(NC=Cc1cc(ccc1OC)O2)=O)=O)=O)=O)N(C)C
N753	A	-0.80888586	CC=C1CN2CCc3c4ccccc4n4C(C1CC2c34)C(=O)OC

		7	
N754	A	-0.812913357	[H]C1CC(C2(C)C3([H])Cc4c(cco4)C([H])(C(=O)OC)C3([H])C([H])C(C2(C1(C)C)O)OC(C)=O)OC(C)=O
N755	A	-0.812913357	[H]C1Cc2c(cc3c(cco3)c2C)C2(C)C(C(C(C(C)(C)C12O)OC(C)=O)OC(C)=O)OC(C)=O
N756	A	-0.812913357	CC(C)=CCc1cc2C3C(COc2cc1O)c1ccc(c(CC=C(C)C)c1O3)O
N757	A	-0.812913357	CC(c1ccccc1)O[C@@H]1CC2C(C(C=C3C[C@H](CC[C@]23C)O[C@H]2[C@@H]([C@@H]([C@@H]([C@@H](CO)O2)O[C@H]2[C@@H]([C@@H]([C@@H]([C@@H](CO)O2)O[C@H]2[C@@H]([C@@H]([C@@H]([C@@H](CO)O2)O)OC)O)OC)O)OC)O)O)[C@]2(CC[C@H](C(C)OC(C)=O)[C@@]12C)O
N758	A	-0.819543936	CC(=C)C1CCC2(CCC3(C)C(CCC4C5(C)C(CCC34C)C(C)(C)C=C5C=O)C12)C(O)=O
N759	A	-0.819543936	CC(C)=CCc1c(cc(cc1O)O)C1=CC(c2c(cc(c(CC=C(C)C)c2O1)O)O)=O
N760	A	-0.819543936	CC(C1CCC2C3CCC4CC(CCC4(C)C3CCC12C)N(C)C(c1ccccc1)=O)NC
N761	A	-0.819543936	CC1(C)C=Cc2c(ccc3C4C(COc23)c2ccc(cc2O4)O)O1
N762	A	-0.819543936	CC1CCC2=C(CCCC2(C)C)C1(C)CCC(C)=CCn1c[n+](C)c2c1c(N)ncn2.[Cl-]
N763	A	-0.819543936	COc1c(cc2c(C(C=C(c3ccc(c(c3)O)O)O2)=O)c1O)O
N764	A	-0.824776463	CC1=CC(c2c3cc(c(c(C)c3oc2C1=O)O)OC)=O
N765	A	-0.824776463	CC1=CC(c2c3cc(c(c(C)c3oc2C1=O)O)OC)=O
N766	A	-0.826074803	CC(C)=CCc1c(cc2c(C(C=C(c3ccc(cc3)O)O2)=O)c1O)O
N768	A	-0.826074803	COc1cc2c(cc1O)C1CC(CC3CCCN13)OC(C=Cc1ccc(c2c1)O)=O

N769	A	-0.826074803	COc1ccc(cc1)[C@@H]1CC(c2c(cc(c(c2O1)[C@@H]1C(c2c(cc(cc2O[C@H]1c1ccc(cc1)O)O)O)=O)O)O)=O
N767	A	-0.826074803	COc1ccc(cc1c1c(cc(c2C(C=C(c3ccc(cc3)O)Oc12)=O)O)O)C1=CC(c2c(cc(cc2O1)O)O)=O
N770	A	-0.832508913	CCC=C(C)C=CCCCC1(CC(C)C(CC(=O)OC)OO1)OC
N771	A	-0.832508913	COc1cc(ccc1O)C1=COc2cc(ccc2C1=O)O
N772	A	-0.838849091	CC(=C)C1CC2=C(c3c(cc(c(c13)O)O)O)Oc1c3C=CC(C)(C)Oc3cc(c1C2=O)O
N773	A	-0.838849091	CC(C)C=Cc1c(cc2c(C(C(CC=C(C)C)=C(c3ccc(cc3O)O)O2)=O)c1O)OC
N774	A	-0.838849091	COc1cc(c(C(C=Cc2cc(c(c(c2)OC)OC)OC)=O)c(c1)OC)O
N775	A	-0.84509804	[H]C(CC(=CCCC(C)=CCCC(C)=CCc1cc(cc(c1O)O)C(O)=O)C(O)=O)C=C(C)C
N776	A	-0.84509804	[H]C1C[C@@H]([C@]2(C)[C@@]3([H])Cc4c(cco4)[C@@]([H])(C(=O)O[H])C3([H])[C@H](C([C@]2(C1(C)C)O)OC(C)=O)O)OC(C)=O
N782	A	-0.84509804	C[C@H]1CC=C[C@H]([C@H]([C@H](CC=Cc2cc(cc(c2C(=O)O1)O)OC)O)O)O
N779	A	-0.84509804	CC(C)=CC(c1c(cc2c(c1C)OC(c1c(C)cc(c(C=O)c1O2)O)=O)O)=O
N778	A	-0.84509804	CC(CCC=C(C)CC[C@@H](C(C)(C)O)O)=CCCC=C(C)CCC=C(C)CC[C@H]([C@](C)(CO)O)O
N777	A	-0.84509804	CC(CCC=C(C)CC[C@H](C(C)(C)O)O)=CCCC=C(C)CCC=C(C)CC[C@H]([C@](C)(CO)O)O
N780	A	-0.84509804	CC1CC=CC(C(C(CC2C(c3cc(cc(c3C(=O)O1)O)OC)O2)O)O)O
N781	A	-0.84509804	CCCCCC1=CC(C(CC=C(C)CCC=C(C)C)=C(C1=O)OC(C)=O)=O
N14	A	-0.847008951	C(c1cc2C(c3cccc(c3C(c2c(c1)O)=O)O)=O)O
N783	A	-0.849113248	CC=C1CN2C3CC1C(CO)(C2Cc1c2ccccc2n(C)c13)C(=O)OC
N784	A	-0.84925765	CC(C)=CCc1c(cc2c(C(c3c(cc(c(c3CC=C(C)C)OC)O)O2)=O)c1O)OC

		8	
N785	A	-0.851258349	CC1C(C(C(C(OCC2C(C(C(C(O2)OC2=C(c3ccc(c(c3)O)O)Oc3cc(cc(c3C2=O)O)O)O)O)O1)O)O)O
N786	A	-0.85308953	CC(C)[C@@H](C)C=C[C@@H](C)[C@H]1CC[C@@]23[C@]4(C=C[C@@]56C[C@H](CC[C@]5(C)[C@]4(CC[C@]12C)OO6)O)O3
N787	A	-0.857050918	CC(C)[C@@H]1CC[C@@]2(CC[C@]3(C)[C@H](CC[C@@H]4[C@@]5(C)CC[C@@H](C(C)(C)[C@@H]5CC[C@@]34C)O)[C@@H]12)CO
N788	A	-0.857332496	[H][C@@]12CC=C(C)[C@@H](C[C@@H](C3=CC(=O)OC3O)O)[C@]2(C)CCCC1(C)C
N789	A	-0.857332496	[H]C(C(=C)[C@@H]1CC[C@]2(C)CC[C@]3(C)[C@]([H])(CC[C@]4([H])[C@@]5(C)CC[C@@]([H])(C(C)(C)[C@]5([H])CC[C@@]34C)O)[C@@]12[H])=O
N792	A	-0.857332496	CC(C)=CC(c1c(C)c2c(c(c1OC)O)OC(c1c(C)c(c(c(C=O)c1O2)O)[Cl])=O)=O
N791	A	-0.857332496	CC=C(C)C(=O)OC1CC2(C)C(C=C(C(CO)=CC3C1C(=C)C(=O)O3)O2)=O
N793	A	-0.857332496	CC1CC2=C(C(C=C(C2=C(C)N1)OC)=O)c1c(C)cc(c2c(cccc12)O)OC
N794	A	-0.860338007	CC(C)=CCc1cc2C(c3c(cc(c(CC=C(C)C)c3O)OC)Oc2c(c1OC)O)=O
N795	A	-0.86332286	c1cc(ccc1C1C(C2C(c3ccc(cc3)O)Oc3cc(cc(c3C2=O)O)O)C(c2c(cc(cc2O1)O)O)=O)O
N796	A	-0.86332286	CC1Cc2cc3c(c(cc(c3c(c2C(=O)O1)O)O)O)c1c(cc(c2c(c3C(=O)OC(C)Cc3cc12)O)O)O
N797	A	-0.86332286	COC(CC1(C=CC(C=C1)=O)O)=O
N798	A	-0.863591927	CN1CCc2cc(c(cc2C1Cc1ccc(cc1)Oc1c2CC3c4c(CCN3C)c(c(c(c4c2cc(c1OC)OC)OC)OC)OC)OC)OC
N799	A	-0.86923172	[H]C1C=C2C[C@H](CC[C@]2(C)C2C[C@H]([C@]3(C)[C@H](CC[C@@]3(C12)O)C(C)OC(C)=O)OC(C)c1ccccc1)O[C@H]1[C@@H]([C@@H]([C@@H]([C@@H](CO)O1)O[C@H]1[C@@H]([C@@H]([C@@H]([C@@H](CO)O1)O[C@H]1[C@@H]([C@@H]([C@@H]([C@@H](CO)O1)O)OC)O)OC)O)OC)O
N800	A	-0.86923172	CC(C)C1CC[C@H]2C(=CC[C@H]3C(C)(C)CCC[C@]23C)C=1
N801	A	-	[H][C@@]12CC=CC([C@]2(C)C=C2C(C1)=C(C)C(=

		0.875061263	O)O2)=O
N802	A	-0.875061263	[H]C1C=C2C[C@H](CC[C@]2(C)C2C[C@H]([C@]3(C)[C@H](CC[C@@]3(C12)O)C(C)OC(C)=O)OC(C)c1ccccc1)O[C@H]1[C@@H]([C@@H]([C@@H]([C@@H](CO)O1)O[C@H]1[C@@H]([C@@H]([C@@H]([C@@H](CO)O1)O[C@H]1[C@@H]([C@@H]([C@@H]([C@@H](CO)O1)O[C@H]1[C@@H]([C@@H]([C@@H]([C@@H](CO)O1)O)O)O)OC)O)OC)O)OC)O
N803	A	-0.875061263	c1c(C(O)=O)c(c2c3C(=O)Oc4c5c(C(=O)Oc(c35)c(c2O)O)c(c2c(cc(c(c2O)O)O)C(O)=O)c(c4O)O)c(c(c1O)O)O
N804	A	-0.875639937	C=CC(CO)C1CCN2CCc3c4ccccc4[nH]c3C2C1
N805	A	-0.880813592	CC(C)=CC(CC(C)=CC(C1COC(C1=C)=O)O)=O
N806	A	-0.880813592	COc1cc(c2C(C=C(c3ccc4c(c3)OCO4)Oc2c1)=O)OC
N808	A	-0.886490725	CC(C)=CCc1cc(cc(CC=C(C)C)c1O)C1CC(c2ccc(cc2O1)O)=O
N809	A	-0.886490725	CC(C)C(C(N(C)C(C(C)C)C(N(C)C(C(C)C)C(N(C)C(Cc1ccccc1)C(N)=O)=O)=O)=O)N(C)C(C(C)CCCCC#C)=O
N813	A	-0.886490725	CC(C)C=Cc1c(cc2c(C(C3CC(C(C)(C)O)Oc4cc(ccc4C=3O2)O)=O)c1O)OC
N810	A	-0.886490725	CC(Cc1ccc(c(c1)O)O)C(C)Cc1ccc(c(c1)O)O
N811	A	-0.886490725	CC1(C)C2=CCC3C(C)(C2CC(C1=O)O)C(CC1(C)C(C(CC13C)O)C(C)(C(C=CC(C)(C)O)=O)O)=O
N812	A	-0.886490725	CC1C(CCC2C1(C)CCC1C2(C)CCC2(C)C3CC(C)(C)CCC3(C)CCC12C)=O
N814	A	-0.886490725	COc1ccc2c(c1)c(c1c3c4c(CCN3)cc3c(c4c4ccc(cc14)OC)OCO3)c1c3c(CCN1)cc1c(c23)OCO1
N815	A	-0.892094603	C(=Cc1ccc(cc1)O)C(c1ccc(cc1O)O)=O
N816	A	-	C1CC2C(C(C1O2)C(O)=O)C(O)=O

		0.892094603	
N817	A	-0.892094603	CC1(C)C2=CCC3C(C)(C2CC(C1=O)O)C(CC1(C)C(C(CC13C)O)C(C)(C(C=CC(C)(C)O)=O)O)=O
N819	A	-0.897627091	CC(=C)C1CC2=C(c3c1c(c1c(C=CC(C)(C)O1)c3O)O)Oc1cc(cc(c1C2=O)O)O
N820	A	-0.897627091	CC(C)C(=O)OC1(c2cccc(c2C(c2c1cc(c(C(=O)OC)c2C)O)=O)O)c1ccc2C(c3cc(c(C(=O)OC)c(C)c3C(c2c1O)=O)O)=O
N818	A	-0.897627091	CC(c1c(cc(c(c2c(C)cc(c3C(c4c(cccc4O)C(c23)=O)=O)O)c1O)OS(O)(=O)=O)O)=O.[Na]
N821	A	-0.897627091	CC1CCC23C(CC(C=C3C(OC2OC(C)=O)OC(C)=O)O)C1(C)CC=C(C)C=C
N822	A	-0.901677032	C[n+]1cc2c3c(ccc2c2ccc4cc5c(cc4c12)OCO5)OCO3
N823	A	-0.903089987	[H][C@@]1(C)C[C@H]([C@]([H])(C=C)C2C=C3c4c(CCN3C(=C)C1=2)c1ccccc1[nH]4)OC1C(C(C(C(CO)O1)O)O)O
N826	A	-0.903089987	C[C@@H]1C(c2cc3c(cc2[C@@H](c2ccc(c(c2)OC)OC)[C@@H]1C)OCO3)=O
N827	A	-0.903089987	C[C@]12C[C@@H](C[C@@]3(C)C1[C@@H]([C@@H]1[C@@]4(COC(C=C24)=O)O1)OC3=O)O
N824	A	-0.903089987	CC=C(C)C(=O)O[C@H]1C[C@]2(C)C(C=C(C(C)[C@@H]([C@@H]3[C@@H]1C(=C)C(=O)O3)O)O2)=O
N825	A	-0.903089987	CCC(C)C(C(N(C)C(Cc1ccccc1)C(NC(CC(C)C)C(Nc1ccccc1C(O)=O)=O)=O)=O)=O)N
N828	A	-0.903632516	C[C@]12C[C@@H](C[C@@]3(C)C1[C@@H](C=C1COC(C=C12)=O)OC3=O)O
N829	A	-0.903632516	C[C@]12C=C[C@@H]([C@@]3(C)C1[C@@H]([C@@H]1[C@@]4(COC(C=C24)=O)O1)OC3=O)O
N830	A	-0.903632516	C[C@]12C=C[C@@H]([C@@]3(C)C1[C@@H](C=C1COC(C=C12)=O)OC3=O)O
N832	A	-0.90363251	C[C@]12C=CC[C@@]3(C)C1[C@@H]([C@@H]1[C@@]4(COC(C=C24)=O)O1)OC3=O

		6	
N833	A	-0.903632516	C[C@]12C=CC[C@@]3(C)C1[C@@H](C=C1COC(C=C12)=O)OC3=O
N831	A	-0.903632516	C[C@]12CC[C@H]([C@@]3(C)C1[C@@H](C=C1COC(C=C13)=O)OC2=O)O
N834	A	-0.903632516	C[C@]12CCC[C@@]3(C)C1[C@@H](C=C1COC(C=C12)=O)OC3=O
N835	A	-0.903632516	CC12CCCC(C)(C1C=CC1(COC(CC12)=O)O)C(O)=O
N836	A	-0.908485019	CCCCCCCCCCCCCCCCCOC1C(C(C(C(COC(CCCCCCCCCCCCCCC)=O)O1)O)O)OC1C(C(C(C(C)O1)OC(C)=O)OC(C)=O)OC(C)=O
N837	A	-0.913813852	c1cc(c(cc1C1=C(C(c2ccc(cc2O1)O)=O)O)O)O
N838	A	-0.913813852	CC(C)=CCc1c(cc(c(C(C=Cc2ccc(cc2)O)=O)c1O)OC)O
N839	A	-0.913813852	CC(C)C1c2nc(cs2)C(NC(C)c2nc(C(NC(C)c3nc(cs3)C(N1)=O)=O)c(C)o2)=O
N840	A	-0.913813852	CC(C)C1c2nc(cs2)C(NC(C)c2nc(C(NC(C)c3nc(cs3)C(N1)=O)=O)c(C)o2)=O
N841	A	-0.919078092	CC1(C)C=Cc2c(ccc3C4C(COc23)c2ccc(cc2O4)O)O1
N842	A	-0.919078092	CC1C(C)C(c2ccc(c(c2)OC)OC)OC1c1ccc(c(c1)OC)OC
N843	A	-0.919078092	CC1CCC2(C)C(C)C=CCC2C1(C)CCC(C)=CCn1c[n+](C)c2c1c(N)ncn2.[Cl-]
N844	A	-0.924279286	[H][C@@]12c3c(cc(c(c3O)O)O)C(=O)O[C@@]1([H])[C@@H]([C@H]([C@H](CO)O2)O)OC(c1cc(c(c(c1)O)O)O)=O
N845	A	-0.924279286	CC[C@H](C)[C@H](C(=O)O[C@@H](CC(C)C)C(N[C@@H](CC(C)C)C(N[C@@H](C)C=CC(N1C(C=C([C@@H]1C)OC)=O)=O)=O)=O)N(C)C
N846	A	-0.929418926	CC1(C)C=Cc2c(cc(c3C(C4CC5c6c(C=4Oc23)c(cc(c6OC5(C)C)O)O)=O)O)O1

N847	A	-0.93027393	[H]c1c(c2C(C3CC4c5c(C=3Oc2c2C=CC(C)(C)Oc12)c(cc(c5OC4(C)C)O)O)=O)O
N848	A	-0.931966115	COc1cc(c2C(C(=COc2c1)c1ccc(cc1)O)=O)O
N849	A	-0.934498451	C(CCCCc1ccccc1)CCCC(c1c(cccc1O)O)=O
N851	A	-0.934498451	CC1(C)CC[C@@]2(CC[C@]3(C)C(=CCC4[C@@]5(C)C[C@H]([C@@H](C(C)(C)C5CC[C@@]34C)O)O)C2C1)C(O)=O
N850	A	-0.934498451	CC1=CC(c2c(CC(C)=O)cc(cc2O1)O)=O
N852	A	-0.934498451	Cc1c2C(CC(C)(C)Oc2cc2c1OC(c1c(C)c(c(c(C=O)c1O2)O)[Cl])=O)=O
N853	A	-0.934498451	Cc1cc(c2c(cccc2c1c1ccc2cc(C)nc(C)c2c1O)O)OC
N854	A	-0.939519253	[H][C@@]12CC[C@@H]([C@](C)(CO)C1=C[C@@H]([C@]1([H])[C@@]2(C)CC[C@]2(C)[C@H](CC[C@@]12C)[C@H](C)C[C@H](C=C(C)C)O)O)O
N855	A	-0.939519253	CC(C)=CCc1c(ccc2C3C(COc12)c1ccc(c(CC=C(C)C)c1O3)O)O
N858	A	-0.939519253	CC(C)=CCCc1cc(cc(c1OC)O)C1=COc2cc(cc(c2C1=O)O)O
N857	A	-0.939519253	CC1C(c2ccccc2)Oc2cc(c(cc12)O)OC
N856	A	-0.939519253	CCC=C[C@H](CC)CCC[C@@]1(CC)C[C@@]2(CC)[C@H](CC(=O)O2)O1
N859	A	-0.944482672	C1C2C(C3C(C(O)O2)OC(c2cc(c(c(c2c2c(cc(c(c2O)O)O)C(=O)O3)O)O)O)=O)OC(c2cc(c(c(c2c2c3C(=O)Oc4c5c(C(=O)Oc(c35)c(c2O)O)c(c2c(cc(c(c2O)O)O)C(=O)O1)c(c4O)O)O)O)O)=O
N860	A	-0.944482672	COc1cc2c(C(C[C@@H](c3ccc(cc3)O)O2)=O)c(c1OC)O
N862	A	-0.949390007	CC(C)=CCc1c2C(c3c(cccc3Oc2c(c(c1OC)O)OC)O)=O
N861	A	-	CN1c2c(cccc2O)C(c2c(cc(c(c12)OC)O)O)=O

		0.949390007	
N863	A	-0.950364854	CC(C1=COCC2C1CC1c3c(CC2N1C)c1cc(CC2C4CC5c6c(CC(C4COC2(C)O)N5C)c2ccccc2n6C)c(cc1[nH]3)OC)=O
N865	A	-0.954242509	CC(C)=CCc1cc2C3C(COc2cc1O)(c1ccc(c(CC=C(C)C)c1O3)OC)O
N864	A	-0.954242509	Cc1cc(c2c(cccc2c1c1c(cc(c2c1C[C@H](C)N(C)[C@@H]2C)OC)OC)OC)OC
N866	A	-0.954242509	COc1ccc(CC(=C)C(=C)Cc2ccc(cc2)O)c(c1)O
N867	A	-0.954242509	COc1ccc(cc1)C1=CC(c2c(cc(c(c2O)O)OC)O1)=O
N868	A	-0.959041392	[H][C@]12[C@H](C(C(=C)C(=O)O2)[C@@H](C2(C)[C@@]1(C)[C@H](CC[C@]2(C)O)O)OC(C(C)=C)=O)OC(C(C)=CC)=O
N869	A	-0.959041392	CC(C)=CCc1cc(ccc1O)C1CC(c2c(cc(c(CC=C(C)C)c2O1)O)O)=O
N871	A	-0.959041392	CC1CC23C4C(C(C)(C=C2C1=O)OC41C(CC(C)C3=O)C1(C)C)O
N870	A	-0.959041392	CCC=C(C)CCC=C(C)CCCCCC(C)=CC=C(C)CCCCCCC(=O)OC
N872	A	-0.963787827	CC1=CCCC2(C)C1=C(C(c1cc3C(C=CC(c3cc12)=O)=O)=O)O
N873	A	-0.968105485	COC1CC2C3(C=C1)C(CN2Cc1cc2c(cc13)OCO2)O
N874	A	-0.968482949	COc1ccc2c(c3ccoc3nc2c1OC)OC
N875	A	-0.969759617	CC(=O)O[C@H]1C[C@H](C(C)(C)C2C[C@H]([C@]3(C)C(CC[C@@]4(C)[C@H](c5ccoc5)OC([C@@H]5[C@]34O5)=O)[C@@]12C)O)OC(C)=O
N876	A	-0.977723605	[H]c1c(cc(c(C(C=Cc2ccccc2)=O)c1O)OC)O
N878	A	-0.97772360	CC1(C)C=Cc2c(ccc(C(CC(c3ccc4c(c3)OCO4)=O)=O)c2OC)O1

		5	
N877	A	-0.977723605	Cc1c(c(C)c2c(C(CC(c3ccccc3)O2)=O)c1O)O
N879	A	-0.981365509	CC(C)=CCCC(C)=CCC[C@]1(C)C=Cc2cc(ccc2O1)O
N880	A	-0.982271233	C1CCN2CC[C@H]3C(=C[C@@](CCC=CC1)([C@H]1[C@@]3(C[C@@H]3C=CCCCCN13)C2)O)[C@@H]1c2c(CCN1CCc1c3c(ccn1)c1ccccc1[nH]3)c1ccccc1[nH]2
N883	A	-0.982271233	CC(c1ccccc1)O[C@@H]1CC2C(C=C[C@@]3(C)C[C@H](CC[C@]23C)O[C@H]2[C@@H]([C@@H]([C@@H]([C@@H](CO)O2)O[C@H]2[C@@H]([C@@H]([C@@H]([C@@H](CO)O2)O[C@H]2[C@@H]([C@@H]([C@@H]([C@@H](CO)O2)O[C@H]2[C@@H]([C@@H]([C@@H]([C@@H](CO)O2)O)O)O)OC)O)OC)O)OC)O)[C@]2(CC[C@H](C(C)OC(C)=O)[C@@]12C)O
N882	A	-0.982271233	CC1(C)C=Cc2cc(C=CC(c3ccc(cc3O)O)=O)ccc2O1
N884	A	-0.985426474	CC(C1=COCC2C1CC1c3c(CC2N1C)c1ccccc1n3C)=O
N885	A	-0.986771734	CC=C(COC1C(C(C(C(CO)O1)O)O)O)C1C=C2c3c(CCN2C(C=1CO)=O)c1ccccc1[nH]3
N886	A	-0.986771734	CC1CCC2C(C=C(C)C(CC=1)O)OC(C2=C)=O
N887	A	-0.991226076	CC1=CC(CC2C(C)(CCC3=CCOC3=O)C(CCC12C)COC(C)=O)=O
N888	A	-0.991226076	CC1=CCC(C2(C)CC3C(C(C12)O)C(=C)C(=O)O3)O
N889	A	-0.995635195	CC(=C)C1CCC2(CCC3(C)C(CCC4C5(C)CCC(C(C)(C)C5CCC34C)O)C12)C(O)=O
N890	A	-0.995635195	CC(C)C=Cc1c(cc2c(C(C3C(C=C(C)C)Oc4cc(ccc4C=3O2)O)=O)c1O)OC
N891	A	-0.995635195	CCCCCCCc1cc(cc(c1)OC(c1c(CCCCCCC)cc(cc1O)O)=O)O

N898	N	-1	CC(C)=CCCc1c(ccc2C[C@@H](COc12)c1ccc(c(c1OC)O)OC)OC
N892	N	-1	CC[C@@H](C)C[C@]1(C)C=CC(=C2C([C@@H](CC(O)=O)OC2=O)=O)O1
N893	N	-1	CC=CC1=CC=CC(=C2C(CNC2=O)=O)O1
N894	N	-1	CC1(C)CC2C3=CCC4C5(C)CCC(C(C)(CO)C5CCC4(C)C3(C)CCC2(C)C(C1)O)O
N895	N	-1	CCCCCCC1C2CC3C4C(C=CC1C24)C(=CC3O)C(O)=O
N897	N	-1	COC1=CC(C(=CC12CCCc1cc(ccc12)O)O)=O
N896	N	-1	COc1cc(c(cc1CCCc1cccc(c1)O)OC)O
N899	N	-1.00106372	C(C=Cc1cnc(N)[nH]1)NC(c1cc(c([nH]1)[Br])[Br])=O
N903	N	-1.004321374	CC(C)(C=C)c1c(cc(c2C(C=C(c3ccc(cc3)OC)Oc12)=O)O)O
N900	N	-1.004321374	CC=CCC=CCCC(C1C(C(N)=O)O1)=O
N902	N	-1.004321374	CCCCCCC=CCCCCCCCc1cccc(c1C(O)=O)O
N901	N	-1.004321374	CCCCCCC=CCCCCCCCCc1cccc(c1C(O)=O)O
N904	N	-1.004349866	CC1C(C)C(c2ccc3c(c2)OCO3)OC1c1ccc(c(c1)OC)OC
N905	N	-1.005995123	CC=C1CN2CCC34C2CC1C1C=C(C2CC56C7CC(C8C=CC(N(C58)c5ccccc56)=O)C(CN27)=CC)C(N(C13)c1ccccc14)=O
N906	N	-1.008600172	CC1CCC2C(C)(C)C3CC12CCC3(C)O
N907	N	-1.012672098	CCCCc1cc(c2C=CC(C)(CCC(C(C)=C)O)Oc2c1OC(C)=O)O
N908	N	-1.012837225	C[C@@H]([C@H]1CC[C@H]2[C@@H]3CC[C@H]4C[C@H](CC[C@]4(C)[C@H]3CC[C@]12C)N(C)C(c1ccccc1)=O)N(C)C
N913	N	-1.012837225	CC(C(=COC)C(=O)OC)=C(C=Cc1ccc(c(c1)OC)[Cl])OC
N914	N	-1.012837225	CC(C(=COC)C(=O)OC)=C(C=Cc1ccccc1)OC

N911	N	-1.012837225	CC(C(C=Cc1ccccc1)OC)C(=COC)C(=O)OC
N909	N	-1.012837225	CC(C)=CCc1cc(C=CC(c2ccc(cc2O)O)=O)cc(c1O)O
N915	N	-1.012837225	CC(C)=CCO[C@H]1COc2ccc(C=CC(=C(C)C(=COC)C(=O)OC)OC)cc2OC1(C)C
N910	N	-1.012837225	CC(C)C1CCC(C)=CC1c1c(cc2c(C(CC(c3ccccc3)O2)=O)c1O)O
N912	N	-1.012837225	COC=C(C(C(C=Cc1ccc(c(c1)OC)[Cl])OC)OC)C(O)=O
N916	N	-1.017033339	CC(C)=CCCC(C)(C1CCC2(C)C1C(CC1C3(C)CCC(C(C)(C)C3CCC12C)O)O)O
N917	N	-1.017033339	CC1(C)C2=CC(C=CC2=c2c(c(c(c3C=CNC1=c23)OC)OC)O)=O
N918	N	-1.017033339	CC1(C)C2CC[C@]3(C)C(C[C@H]([C@@H]4[C@H](CC[C@@]34C)[C@]3(C)CC[C@H](C(C)(C)O)O3)O)[C@@]2(C)CC[C@H]1O
N919	N	-1.021189299	[H]C1CC(C)(C)C2([H])CCC3=C[C@](C)(C[C@H]([C@]3([H])[C@@]2(C)[C@H]1O)O)C=C
N920	N	-1.021189299	CC1=C(C(C2CCc3cc(ccc3OC=2C1=O)O)=O)OC
N922	N	-1.021189299	CC1=CC(NC2=C1C(c1ccccc1C2=O)=O)=O
N921	N	-1.021189299	Cc1cc(c2c(cccc2c1c1ccc2cc(C)nc(C)c2c1O)OC)OC
N923	N	-1.021841531	COc1ccc(cc1)C1=CC(=O)Oc2c1c(cc(c2OC)OC)OC
N924	N	-1.023268633	CC(C#CC#CC#CC#CCCCCCCCC(O)=O)O
N925	N	-1.025172946	CC1(C)[C@H](CC[C@@]2(C)[C@H]1CC[C@]1(C)[C@@H]2CC[C@@H]2[C@H]3[C@@H](CC[C@]3(C)CC[C@@]12C)C(=C)C=O)O
N926	N	-	CN1Cc2c(ccc(c2OC)OC)c2ccc3cc4c(cc3c12)OCO4

		1.025305865	
N927	N	-1.026328939	CC1C(C(C(C(O1)OC1=C(c2ccc(cc2)O)Oc2cc(cc(c2C1=O)O)O)O)O)O
N928	N	-1.026875996	CC1(C)C=Cc2cc3C(C([C@@H](c4ccccc4)Oc3cc2O1)O)OC
N929	N	-1.029383778	CC1C(C)C(c2ccc3c(c2)OCO3)OC1c1ccc(c(c1)OC)OC
N930	N	-1.029520557	COC(C(=C)C1C(C2C(=C)C(=O)OCC2(CC1OC(C(=C)CO)=O)C=C)O)=O
N931	N	-1.034894235	CC(=C)C(CC=C(C)C)Cc1c(cc(c2C(CC(c3c(cccc3O)O)Oc12)=O)O)O
N932	N	-1.037426498	CCC=CCC=CCC=CCCCCCCCC(=O)OC
N933	N	-1.037426498	CCCCCC1=CC(C=C(C1=O)OC)=O
N934	N	-1.037426498	COc1cc2C3CC(CC4CCCCN34)OC(C=Cc3ccc(c(c3)c2cc1OC)O)=O
N935	N	-1.037824751	COc1ccc(cc1)C1=COc2cc(c(cc2C1=O)OC)O
N936	N	-1.041392685	[H][C@]12C[C@@H](C)C([C@@]34C[C@H](C)C(C3=C[C@@]3(C)[C@@H]([C@@]4([H])[C@@]2(C1(C)C)O3)O)=O)=O
N937	N	-1.041392685	C1=C(c2ccc(c(c2)O)O)Oc2cc(cc(c2C1=O)O)O
N940	N	-1.041392685	C1=C(c2ccc(c(c2)O)O)Oc2cc(cc(c2C1=O)O)O
N938	N	-1.041392685	CCCCCCC1C2CC3C4C(C=CC1C24)C(=CC3O)C(O)=O
N939	N	-1.041392685	COc1cc(c(cc1OC)O)[C@@H]1COc2cc(ccc2C1=O)O
N941	N	-1.05307844	[H][C@]12[C@@H]([C@H]([C@H](COC(c3ccc(cc3)O)=O)O[C@]1([H])c1c(cc(c(c1O)O)O)C(=O)O2)O)O

		4	
N942	N	-1.056904851	CC1C=CNC2C=1C(C1=CC=C(C(C1=2)=O)OC)=O
N943	N	-1.056904851	CCCCCc1cc(c2C(C(CCC(C)=O)C(C)(C)Oc2c1)=O)O
N944	N	-1.056953365	COc1cc(C=CC(CC(C=Cc2ccc(c(c2)OC)O)=O)=O)ccc1O
N945	N	-1.058978686	CC1(C)C(CC(=O)OC)C2(C)C3CCC4(C)C(c5ccoc5)OC(CC4(C3=C)OC2CC1=O)=O
N946	N	-1.071882007	CC(c1cc2C(c3ccc(c(c3C(c2o1)=O)OC)O)=O)=O
N947	N	-1.074484805	CC(=O)OC1C2C(C)(C)C(CC[C@]2(C)C2CCC3(C)C(CC=C3C2C1O)c1ccoc1)=O
N950	N	-1.079181246	C(C(O)=O)c1cc(ccc1O)O
N948	N	-1.079181246	C[C@H]1[C@H](C)[C@@H](c2ccc(c(c2)O)O)O[C@@H]1c1ccc(c(c1)O)O
N949	N	-1.079181246	C=CC(C=Cc1ccc(cc1)O)c1ccc(cc1)O
N951	N	-1.079181246	C1C2C(Cc3ccc4c(c3)OCO4)C3C=CC4C(C1C(C=C4C(O)=O)=O)C23
N952	N	-1.079181246	COc1cc(C2=Cc3ccc(cc3OC2=O)O)c(cc1O)O
N953	N	-1.079181246	COc1cc(ccc1O)C1C(COC(c2ccccc2)=O)c2cc(C=CCOC(c3ccccc3)=O)cc(c2O1)OC
N954	N	-1.086359831	[H]C(c1cc2c3ccc(cc3[nH]c2cc1O)OC)=O
N955	N	-1.086359831	CC(C)=CCc1c(cc2c(c1C)OC(c1c(C)c(c(c(C=O)c1O2)O)[Cl])=O)O
N956	N	-1.088789974	[H][C@]12C(C)C(C=C(C)[C@]1(C)C[C@]([H])([C@@H](C)[C@]2(C)CC[C@H](C)CC(O)=O)O)=O

N957	N	-1.089905111	Cc1cc(c(C=O)c2c1C(=O)Oc1c(cc3c(C(CC(C)(C)O3)=O)c1C)O2)O
N958	N	-1.089905111	CN1c2ccccc2C(c2c1cc(c(c2O)OC)OC)=O
N959	N	-1.092233986	[H]C1=C(c2cc([H])c(c(c2)OC)O)Oc2c([H])c(c([H])c(c2C1=O)OC)OC
N960	N	-1.093421685	[H][C@]12CCN3CCCCC=CCC[C@](C=C1C1c4c(CCN=1O)c1ccccc1[nH]4)([C@H]1[C@@]2(CCC=CCCCCN1)C3)O
N961	N	-1.103803721	[H]c1cc(cc(CC=C(C)C)c1C)C(=O)OC
N964	N	-1.103803721	C(CCCCCCCCCCCCC(=O)OCC(CO)O)CCCCCCCCCCCCO
N962	N	-1.103803721	C[C@@H]1C2C3=CCC4[C@@]5(C)CC[C@@H]([C@@](C)(CO)C5CC[C@@]4(C)[C@]3(C)CC[C@]2(CC[C@H]1C)C(O)=O)O
N963	N	-1.103803721	CCC1=COCC2C1CC1c3c(CC2N1C)c1ccccc1n3C
N965	N	-1.10720997	CC(C)=CCc1c(cc(c2C(C(COc12)(c1ccc(c(CC=C(C)C)c1OC)OC)O)=O)O)O
N966	N	-1.10720997	COc1c2c(c3c4ccccc4C(c4c3c1ccn4)=O)OCO2
N967	N	-1.113943352	CCCCCCC1C2CC3C=CC(C4C=CC1C2C34)C(O)=O
N968	N	-1.117271296	[H]N12C=C(C(C)C)C3CC1c1c(CC2C3(C)CO)c2ccccc2n1C
N969	N	-1.120573931	C(C1C(C(C(C(O1)Oc1cc(C=Cc2ccc(cc2)O)cc(c1)O)O)O)O)O
N970	N	-1.123851641	CCC=CCC=CCC=CCCCCCCCCCc1cccc(c1C(O)=O)O
N971	N	-1.133079407	CCCCCCC1=CC(=C(CCCCCC)C(=O)O1)O
N972	N	-1.133538908	CC(C)=CCc1cc(cc(c1O)OC)C1CC(c2ccc(cc2O1)O)=O

N973	N	-1.146128036	C1C2C=CC(C3C=CC4C(Cc5ccc6c(c5)OCO6)C1C4C23)C(O)=O
N974	N	-1.146128036	CCCCCCCCCCCCCC1CC(NCCCNCCCCNCCCN1)=O
N975	N	-1.149619277	CC(=O)OC(C1C(C)(C)C(C2CC3C(CCC4(C)C(c5ccco5)OC(CC=34)=O)C1(C)C2=O)O)C(=O)OC
N976	N	-1.152288344	CC=Cc1ccc(cc1)O
N977	N	-1.161368002	C([C@@H]1[C@H]([C@@H]([C@@H](O)O1)O)O)OC1=C(c2ccc(c(c2)O)O)Oc2cc(cc(c2C1=O)OC(c1cc(c(c(c1)O)O)O)=O)O
N978	N	-1.163811455	[H][C@]12CCC=C(COC(C=Cc3ccc(c(c3)O)O)=O)[C@]1(C)C[C@H]([C@@H](C)[C@]2(C)CC[C@H](C)CC(O)=O)O
N979	N	-1.167317335	CN1c2ccccc2C(c2c1c(c(c(c2O)OC)OC)OC)=O
N980	N	-1.167317335	COc1cc(ccc1O)C1=C(C(c2c(cc(cc2O1)O)O)=O)O
N981	N	-1.176091259	C[C@H]1C([C@@H](C[C@H]2[C@]3(C)CC[C@@]4(C)C5CC(C)(C)CC[C@@]5(CC[C@]4(C)[C@H]3CC[C@]12C)CO)O)=O
N982	N	-1.176091259	C1=C(c2ccc(cc2)O)Oc2c(C3C(c4ccc(cc4)O)Oc4cc(cc(c4C3=O)O)O)c(cc(c2C1=O)O)O
N985	N	-1.176091259	CC(=C)C(CC=C(C)C)C[C@@]12C[C@@H](CC=C(C)C)C(C)(C)[C@@](CC=C(C)C)(C(C(=C1O)C(c1ccc(c(c1)O)O)=O)=O)C2=O
N983	N	-1.176091259	CC(C)=CC(CC(=CCCC(C)=CCCC(C)=CCc1cc(cc(c1O)O)C(O)=O)C(O)=O)O
N984	N	-1.176091259	CC(C)=CC[C@@H]1C[C@]2(CC3CCC(C)(C)C=C3C)C(=C(C(c3ccc(c(c3)O)O)=O)C([C@@](CC=C(C)C)(C2=O)C1(C)C)=O)O
N986	N	-1.176091259	CC1C(CCC2C1(C)CCC1C2(C)CCC2(C)C3CC(C)(C)CCC3(CCC12C)C=O)=O
N987	N	-1.176091259	CCCCCCCCCCCCCCCC1CC(NCCCNCCCCNCCCN1)=O
N988	N	-	CCC(C)C1C(N(C)C(Cc2ccccc2)C(=O)OC(C(C)C)C(N

		1.17719176 5	(C)C(Cc2ccccc2)C(=O)OC(C(C)C)C(N(C)C(Cc2cccc c2)C(=O)O1)=O)=O)=O
N989	N	- 1.18103920 6	C1=C(C(c2c(cc(cc2O1)O)O)=O)c1ccc(cc1)O
N990	N	- 1.18403466 5	CC1CC2C(CC=C1C=CC(C)=O)C1(C(=CC3(C(C)CC 4C(CC3O1)C(=C)C(=O)O4)O)C(C)=O)C(=O)O2
N991	N	- 1.18469143 1	CC1=CC2C(CCC(=C)C(CC1O)O)C(=C)C(=O)O2
N992	N	- 1.19033169 8	[H]C(c1cc2c3ccc([H])cc3[nH]c2cc1O)=O
N993	N	- 1.19865708 7	CN1c2ccccc2C(c2c1cc(c(c2O)OC)OC)=O
N994	N	- 1.19970977 1	CC1Cc2c(c3ccc(c4c(cc(C)cc34)O)OC)c(cc(c2C(C)N1)OC)O
N995	N	- 1.20006988 2	COc1cc2c(C(c3ccccc3N2)=O)c(c1OC)O
N996	N	- 1.20139712 4	Cc1cc(c2c(cc(cc2c1c1c(cc(c2c1C[C@H](C)N(C)[C@ @H]2C)OC)OC)OC)OC)OC
N997	N	- 1.20311577 8	CC(=C)[C@H](CC=C(C)C)Cc1c(cc(c2C(C[C@@H](c 3c(cccc3O)O)Oc12)=O)O)OC
N100 2	N	- 1.20411998 3	C[C@@](C=C)([C@H](C=CC(=C[Cl])C([Cl])[Cl])[Cl])[Cl]
N998	N	- 1.20411998 3	CC(C)=CCc1c(c(CC=C(C)C)c2c(C(c3cccc(c3O2)O)= O)c1O)O
N999	N	- 1.20411998 3	CC1C(C(CC2C1(C)CCC1C2(C)CCC2(C)C3CC(C)(C) CCC3(C)CCC12C)O)=O
N100 0	N	- 1.20411998 3	CC1C(CCC2C1(C)CCC1C2(C)CCC2(C)C3CC(C)(C) CCC3(C)CCC12C)=O
N100 1	N	- 1.20411998 3	CC1CCC2(C(C)C3C(CC4C5=CCC6=CC(C(CC6(C)C 5CC(C34C)O)O)=O)O2)OC1
N100 3	N	- 1.21218760	[H][C@@]12CCN3CCCCC=CCC[C@]4([C@H]([C@ @]2([H])[C@@]2([H])c5c(CCN2CCc2c6c(ccn2)c2ccc

		4	cc2[nH]6)c2ccccc2[nH]5)O4)[C@H]2[C@@]1(C[C@]1([H])C=CCCCN12)C3
N1005	N	-1.212187604	C[C@@H](CC(c1c(cc2c(C=CC(C)(C)O2)c1O)OC)=O)c1ccccc1
N1004	N	-1.212187604	CC(C)=CCc1cc(cc(c1C)O)C(=O)OC
N1006	N	-1.217483944	CC(CCC=C(C)C)c1ccc(C)cc1O
N1007	N	-1.217747073	CC(C)CC(=O)OC1C2C(C=C(C)C3=CC(C(C)(C1O)O3)=O)OC(C2=C)=O
N1008	N	-1.227886705	COc1cc2c(C(C=C(c3ccc(cc3)O)O2)=O)c(c1OC)O
N1009	N	-1.228400359	CC1(C)C=Cc2c(ccc(C(CCc3ccc(cc3)OC)=O)c2O)O1
N1010	N	-1.230448921	C[C@](C=C)([C@@H](C=CC(=C)C([Cl])[Cl])[Cl])[Cl]
N1011	N	-1.230448921	C1=C(c2ccc(c(c2)O)O)Oc2c(C3C(c4ccc(cc4)O)Oc4cc(cc(c4C3=O)O)O)c(cc(c2C1=O)O)O
N1012	N	-1.230448921	CC(=O)OC1CCC2(C)C(CCC3(C)C2CC=C2C4CC(C)(C)CCC4(CCC23C)C(Nc2ccc(C)cc2)=O)C1(C)C
N1013	N	-1.230448921	CC1C(CCC2C1(C)CCC1C2(C)CCC2(C)C3CC(C)(C)CCC3(CCC12C)CO)=O
N1014	N	-1.230448921	COc1cc2c(C(c3ccccc3N2)=O)c(c1OC)O
N1015	N	-1.230515667	c1cc(c2C(c3cc(ccc3Oc2c1)O)=O)O
N1016	N	-1.230704314	CC1(C)C=Cc2c(ccc(C(CCc3ccc(c(c3)O)O)=O)c2O)O1
N1017	N	-1.237907154	CCCC(=O)OC1(C)CCC2C(C)C(=O)OC3(C)CCC(C(=C)CC4C1C2C3O4)OC(C)=O
N1018	N	-1.24054924	C1=C(c2ccc(cc2)O)Oc2cc(cc(c2C1=O)O)O

		8	
N1019	N	-1.247814115	CC(=O)O[C@H]1C[C@H](C(C)(C)C2C[C@H]([C@]3(C)C(CC[C@@]4(C)[C@H](c5ccoc5)OC([C@@H]5[C@]34O5)=O)[C@@]12C)O)OC(C)=O
N1020	N	-1.252607094	CCCCCc1cc(c(CC=C(C)CCC=C(C)C)c(c1O)OC(C)=O)O
N1021	N	-1.252847634	CC(C)=CCc1c(c(C=O)cc2c3ccccc3[nH]c12)O
N1022	N	-1.252853031	[H]C(c1c2c(cc(cc2oc1c1ccc(c(c1OC)O)O)OC)O)=O
N1025	N	-1.255272505	CC(C)=CCOc1cc(c2C(c3c(cc(C)cc3Oc2c1)O)=O)O
N1024	N	-1.255272505	CC1(C)[C@H](Cc2cc(C[C@@]3(C(=C(C(=C)O3)O)c3ccc(cc3)O)C(=O)OC)ccc2O1)O
N1023	N	-1.255272505	CC1CCC=C2C1(C)CCC(C)C2(C)CCC(C)=CCn1c[n+](C)c2c1c(N)ncn2.[Cl-]
N1026	N	-1.257678575	C(C1[C@H](C(C([C@H](c2c(cc3c(C(c4cc(c(cc4O3)O)O)=O)c2O)O)O1)O)OC(C=Cc1ccc(cc1)O)=O)O)O
N1027	N	-1.259699298	CC(=C)C1CC2=C(c3c1c(c1c(C=CC(C)(C)O1)c3O)O)Oc1cc(cc(c1C2=O)O)O
N1028	N	-1.278753601	CC12CCCC(CO)(C1C(CC1CC(=C)C3CCC12C3)OC(c1ccccc1)=O)C(O)=O
N1029	N	-1.278753601	CCCCCCCCC[C@]1(CC[C@@H](C)C(=O)O[C@@](CCCCCCCCC)(CC[C@@H](C)C(=O)O1)CO)CO
N1030	N	-1.283301229	CC(C)=CCc1c(C=Cc2ccccc2)cc(cc1OC)O
N1031	N	-1.284430734	C1CCc2ccccc2C1
N1032	N	-1.295324839	CC1CC=CC(C(CCC=Cc2cc(cc(c2C(=O)O1)O)O)O)O
N1033	N	-1.29666519	CC1(C)C2CC[C@]3(C)C(CCC4C5C(CC[C@]5(C)CC[C@@]34C)C(C)(C)O)[C@@]2(C)CC[C@H]1O
N103	N	-	Cc1c(cc(c2C(C[C@@H](c3ccccc3)Oc12)=O)OC)OC

4		1.29666519	
N1035	N	-1.297042082	CC([C@H]1CC[C@@]2(C3CC=C4C[C@H](CC[C@]4(C)C3C[C@H]([C@]12C)Oc1ccccc1)OC1C(C(C(C(CO)O1)O)O)O)O)OC(C)=O
N1037	N	-1.301029996	CC1(C)C=Cc2c(cc(c3C(CC(c4ccccc4)Oc23)=O)O)O1
N1036	N	-1.301029996	CC1(C)C=Cc2c(ccc3C(C4C(COc5cc(c(cc45)OC)OC)Oc23)=O)O1
N1038	N	-1.301029996	CC1(C)C2CC=C(C=O)C1C2
N1039	N	-1.301029996	CC1(C)C2CC=C(CO)C1C2
N1040	N	-1.301029996	CC1(C)C2CC1C(=C)C(C2)O
N1041	N	-1.301029996	CN(CCc1cc(c(c(c1)[Br])OCCCNC(C1CC2(CC(=C(C(=CO2)[Br])OC)[Br])ON=1)=O)[Br])C(N)=O
N1042	N	-1.303181748	c1cc(c(cc1C1=C(C(c2c(cc(cc2O1)O)O)=O)O)O)O
N1043	N	-1.311753861	C=C(Cc1ccc(cc1)O)C(=C)Cc1ccc(cc1)O
N1044	N	-1.322219295	CC(C)=CCCc1c(ccc2c3COc4cc(ccc4c3oc12)O)OC
N1045	N	-1.324282455	C[C@@H]1C[C@@]2(C)C(CC[C@]3(C)C2CC=C2C4CC(C)(C)CC[C@@]4(CC[C@]23C)C(O)=O)C(C)(C)[C@H]1O
N1046	N	-1.325773959	[H]C1=C(c2ccc(cc2)O)Oc2c(CCC=C(C)C)c(c(CCC=C(C)C)c(c2C1=O)O)O
N1047	N	-1.330436191	CCCC(=O)OC1(C)CCC2C(C)C(=O)OC3(C)CCC(C(=C)CC4C1C2C3O4)O
N1048	N	-1.333129942	C([C@@H]1[C@H]([C@@H]([C@H]([C@H](Oc2cc(c3C(C(=C(c4ccc(c(c4)O)O)Oc3c2)O)=O)O)O1)O)O)O)O
N1049	N	-1.336459734	CC1C(C(C(C(O1)OC1C(c2ccc(cc2)O)Oc2cc(cc(c2C1=O)O)O)O)O)O

N1050	N	-1.339025844	[H][C@]12C(C)C(C=C(C)[C@]1(C)CC([C@@H](C)[C@]2(C)CC[C@H](C)CC(O)=O)(O)O)=O
N1051	N	-1.342422681	[H][C@@]12CCc3cc4c(cc3[C@]1(C(c1ccc3c(C=CC(C)(C)O3)c1O2)=O)O)OCO4
N1052	N	-1.342422681	CC(C)=CC(c1c(C)c2c(c(c1OC)O)OC(c1c(C)cc(c(C=O)c1O2)O)=O)=O
N1053	N	-1.352182518	[H]c1cc(cc2c1C(C(=CO2)c1cc([H])c(c(CCC=C(C)C)c1)OC)=O)O
N1054	N	-1.355920837	CCC=CC(CC)CC=CC1(CC)CC(CC)C(CC(=O)OC)OO1
N1055	N	-1.35836451	CC1C(c2cc3c(cc2c2cccc(c12)N)OCO3)=O
N1056	N	-1.360418472	CC1C2CCC(C)(C3C2C2C(C)(CCC(C(=C)CC3O2)O)OC1=O)OC(C)=O
N1057	N	-1.361727836	CC1CC(C2(COC(C)=O)C(CCCC23CO3)C1(C)CCC1COC(C=1)=O)OC(C)=O
N1058	N	-1.36512252	CC(=C)C(=O)OC1CC23C(CCC(COC2O)=CC2C1C(=C)C(=O)O2)O3
N1059	N	-1.367542274	C[C@H]1[C@@H]([C@H]([C@H]([C@@H](O1)O[C@H]1C(c2c(cc(cc2O[C@@H]1c1ccc(c(c1)O)O)O)O)=O)O)O)O
N1060	N	-1.371499665	[H]c1cc(C=CC(=O)OC[C@@]2(C)CCC[C@@]3(C)[C@]([H])(CC[C@H](C)CC(O)=O)C(=C)[C@@H](C[C@]23[H])O)ccc1O
N1061	N	-1.374748346	CC(CO)=CCc1c(cc2c(C(C(CO2)c2ccc(cc2O)O)=O)c1O)O
N1062	N	-1.376576957	C([C@@H]1[C@H]([C@@H]([C@H]([C@H](c2c(cc3c(C(c4cc(c(cc4O3)O)O)=O)c2O)O)O1)O)O)O)O
N1063	N	-1.380211242	CCOC(CC(C=C)=CC[C@H]1C(=C)CCC2C(C)(C)CCC[C@]12C)OCC
N1064	N	-1.380979773	CCCCCC=CC=CC(NCC(C)C)=O
N1065	N	-1.383399246	CCCCCC=CC=CC(NCC(C)C)=O

N1066	N	-1.389166084	CC(C)(C(COc1ccc2c(c3ccoc3nc2c1OC)OC)O)O
N1067	N	-1.389166084	CC(C)(C(COc1ccc2c(c3ccoc3nc2c1OC)OC)O)O
N1068	N	-1.389166084	CC1C(C(C(C(O1)OC1CCC2(C)C(CCC3(C)C2C=CC24C5CC(C)(C)CCC5(CO4)C(CC23C)O)C1(C)CO)O)OC1C(C(C(C(CO)O1)O)O)O)OC1C(C(C(CO1)O)O)O
N1069	N	-1.390935107	CC1(CCC2C(C1)=CCC1C(C)(C)C(CCC12C)O)C=C
N1070	N	-1.391366611	C1CCN2C3CC4(C2C1)C(C=C3)=CC(=O)O4
N1071	N	-1.392696953	COc1cc(c2C(=CC(=O)Oc2c1)c1ccc(c(c1)O)OC)OC1C(C(C(C(CO)O1)O)O)O
N1072	N	-1.392725984	COc1cc2cc3COC(c3c(c3ccc4c(c3)OCO4)c2cc1OC)=O
N1073	N	-1.394451681	COc1cc(c(cc1C1=COc2cc(cc(c2C1=O)O)O)O)OC
N1074	N	-1.397940009	[H]C1=C(C(C=C(CC(C)(C)O)NC1=O)=O)O
N1075	N	-1.397940009	C(C1C(C(C(C(OCC2C(C(C(C(O2)OC2=Cc3c4c(ccn3)c3ccccc3n4C2=O)O)O)O)O1)O)O)O)O
N1076	N	-1.397940009	CC(C)(CC1=CC(C(=C(C(N1)=O)[Cl])O)=O)O
N1077	N	-1.397940009	CC1(C)CCCC2(C)C1CCC1(CO1)C2CC=C(CC=O)C=O
N1078	N	-1.401240165	CC(=C)C1CCC2(C)CCC(C(C)=C2C1)=O
N1079	N	-1.401400541	[H][C@]12C[C@@]3(C)C(C=CC[C@]3([H])CC1=C(C)C(=O)O2)=O
N1080	N	-1.408239965	CC(C)c1ccc2c(CCC3C(C)(CCCC23C)CO)c1
N108	N	-	CC(C)=CCc1c(ccc2C(C3(C(COc4cc5c(cc34)OCO5)

1		1.411619706	Oc12)O)=O)OC
N1082	N	-1.413299764	COc1cc(c2C(=CC(=O)Oc2c1)c1ccc(c(c1)O)O)OC1C(C(C(C(CO)O1)O)O)O
N1083	N	-1.414973348	C[C@@]1(C)CCC[C@]2(C)[C@@H](C3C=C(CC(O)OO3)C=O)C(=C)CC[C@@H]12
N1084	N	-1.419158855	CC(C)=CCc1c(cc(C=Cc2ccc(cc2O)O)cc1O)O
N1085	N	-1.419671922	CC(C)(C=C)C1=Cc2c(c3C=CC(C)(C)Oc3c(c2OC1=O)C(C)(C)C=C)O
N1086	N	-1.423245874	C(C1[C@H](C(C([C@H](c2c(cc3c(C(c4cc(c(cc4O3)O)O)=O)c2O)O)O1)O)OC(c1ccc(cc1)O)=O)O)O
N1087	N	-1.426741498	[H][C@]12CC[C@@]3(C)[C@H](c4ccco4)OC([C@@H]4[C@@]3([C@]1(C)C(C[C@@]1(C)C(C)(C)O[C@@H](CC(=O)OC)[C@@]12CO)=O)O4)=O
N1088	N	-1.431363764	CC1(C)C=Cc2c(ccc3C(C(=COc23)c2cc(c(cc2OC)OC)OC)=O)O1
N1089	N	-1.43837687	CN1CCC23C4C5C(CC2=O)C(C1)=CCOC5CC(N4c1ccccc13)=O
N1090	N	-1.444044796	COc1c2c3ccccc3C(c3c2c(ccn3)c(c1OC)OC)=O
N1091	N	-1.447158031	CC(CCCC(C)(C)O)=CCc1c2C(c3c(cc(cc3Oc2cc(c1OC)O)O)O)=O
N1092	N	-1.450459845	CCC1CC2(CC)C3C1C(C(C)O)OOC3(CC)C(=CC(=O)OC)O2
N1093	N	-1.470514028	CC=C1CN2CCC34C2CC1C(CO)C3N(C(C)=O)c1ccccc14
N1094	N	-1.474216264	C1=C(c2ccccc2)Oc2cc(cc(c2C1=O)O)O
N1095	N	-1.475671188	CN1c2c(cccc2O)C(c2c(cc(c(c12)OC)OC)O)=O
N1096	N	-1.477121255	C1=C(c2ccc(cc2)O)Oc2c(C1=O)c(cc(c2c1c(cc(c2C(C=C(c3ccc(cc3)O)Oc12)=O)O)O)O)O

N1097	N	-1.477907297	CC1CCC2C1CC1(C)CC=C(C(C)C(C(C=C(C)C(O)=O)O)O)C1CC=C2C(O)=O
N1098	N	-1.478984506	C=C(CO)C(=O)OC1CC23C(CCC(COC2O)=CC2C1C(=C)C(=O)O2)O3
N1099	N	-1.48128478	CC1(C)C=Cc2c(cc(C=Cc3ccc(cc3O)O)cc2O1)O
N1100	N	-1.481442629	C[C@H]1CC[C@@H]2[C@@H](C)C[C@H]3[C@@H]4C(C)(C)C(C([C@]4(C)C([C@@]23C1=O)=O)=O)=O
N1101	N	-1.481619031	CC(C)(C=Cc1c(cc(c2C=CC(=O)Oc12)OC)OC)O
N1102	N	-1.486268106	CC(C)(C=C)c1c2c(C=CC(=O)O2)c(c2C=CC(C)(C)Oc12)OC
N1103	N	-1.491361694	CC1(C)CCC2(C)CCC3(C)C4CC=C5C(CCC(C5(C)C)O)C4(C)CCC3(C)C2C1
N1104	N	-1.4915523	CCC(CC1(CC)C=C(CC)C(=CC(=O)OC)O1)C=CC(C)O
N1105	N	-1.497087275	CC1CC2C(CC=C1CCC(C)=O)C(=C)C(=O)O2
N1106	N	-1.50242712	CC1C[C@@]23C(C(=C(C)C([C@@]34[C@@H](C[C@H](C)[C@H]4CC[C@@H]2C)C=1)=O)O)=O
N1107	N	-1.51315757	COc1c(C=O)c(cc2C(c3ccccc3C(c12)=O)=O)O
N1108	N	-1.5132176	COc1cc2CCN3Cc4c(CC3c2cc1OC)ccc(c4OC)OC
N1109	N	-1.51851394	CC1(C)CCc2c(ccc3C[C@@H](COc23)c2ccc(c(c2OC)O)OC)O1
N1110	N	-1.518714796	[H][C@@]12CCC(=C)[C@@]([H])(CC[C@H](C)CCOC(C=Cc3ccc(c(c3)O)O)=O)[C@]2(C)CCC[C@]1(C)C(O)=O
N1111	N	-1.520964492	CC=C(C)C(=O)OC(C)c1cc2C=CC(C)(C)Oc2cc1OC
N1112	N	-1.522444234	CC1(C)C=Cc2c(ccc3C(C4=C(COc5cc6c(cc45)OCO6)Oc23)=O)O1
N1113	N	-1.522444234	CC1(C)C=Cc2c(ccc3CC(COc23)c2ccc(c(c2OC)O)OC)O1
N111	N	-	CC(C)=CCc1c(cc(C=Cc2ccccc2)cc1OC)O

4		1.53529412	
N1115	N	-1.556302501	CC1CCC2(CCC3(C)C(=CCC4C5(C)CCC(C(C)(C)C5CCC34C)O)C2C1C)C(O)=O
N1116	N	-1.57054294	CC#CC#CC#Cc1ccccc1
N1117	N	-1.576248058	Cc1c2c(ccn1)c1ccc(cc1[nH]2)OC
N1118	N	-1.578595044	CC(=O)OC1CC2C(C)(C=CC(=O)OC2(C)C)C2C(CC3(C)C(c4ccoc4)OC(C4C3(C12C)O4)=O)O
N1119	N	-1.579783597	CCC=CCC=CCC=CCCCC(NCCc1ccc(cc1)O)=O
N1120	N	-1.595496222	c1ccc(cc1)C1=C(C(c2c(cc(cc2O1)O)O)=O)O
N1121	N	-1.602059991	C(C1[C@H](C(C([C@H](c2c(cc3c(C(c4cc(c(cc4O3)O)O)=O)c2O)O)O1)O)O)O)OC(C=Cc1ccc(cc1)O)=O
N1122	N	-1.605305046	CN1CCc2cc(c(cc2C1Cc1cc(c(cc1Oc1cc2CC3c4c(CCN3C)cc(c(c4c2cc1OC)OC)OC)OC)OC)OC)OC
N1123	N	-1.611723308	COc1ccc2C(C=C(c3ccccc3)Oc2c1OC)=O
N1124	N	-1.612783857	C[C@H]1C[C@H]2C[C@@H](C)[C@](C)([C@H]3CC[C@H]4[C@H]([C@H]23)[C@@H]1CC[C@]4(C)NC=O)NC=O
N1125	N	-1.613841822	CCC1CN2CCc3c4ccccc4[nH]c3C2CC1C(=COC)C(=O)OC
N1126	N	-1.615950052	CC=C1CN2CC[C@@]34C(=C(C(=O)OC)[C@H]1C[C@H]24)Nc1ccccc13
N1127	N	-1.622707889	[H][C@@]1(CC[C@H](C)CC(O)=O)C(=C)[C@@H](C[C@]2([H])[C@](C)(CCC[C@@]12C)COC(C=Cc1ccc(c(c1)O)O)=O)O
N1128	N	-1.625089987	CC1COC2=C1C(C(c1c2ccc2c1CCCC2(C)C)=O)=O
N1129	N	-1.632659713	CC1=CC(Nc2c1c(c1ccc(c(c1c2OC)OC)OC)OC)=O
N113	N	-	CCC=CCC=CCCC=CC=CC(NCC(C)C)=O

0		1.633468456	
N1131	N	-1.638659296	[H][C@]12C(C)C(=[H])C=C(C)[C@]1(C)C[C@]([H])([C@@H](C)[C@]2(C)CC[C@H](C)CC(O)=O)O
N1132	N	-1.64246452	COc1c2c(cc3c1c1C(CO)Oc4cc(ccc4c1o3)O)OCO2
N1133	N	-1.643452677	C=CC1C(OC=C2C(=O)OCCC12O)OC1C(C(C(C(CO)O1)O)O)O
N1134	N	-1.646649477	COc1ccc(cc1)C1=CC(c2c(cc(cc2O1)O)O)=O
N1135	N	-1.650412133	CCCCCCC1=C(CC(CCCCC)OC1=O)O
N1136	N	-1.653212514	CCCCCCCCCC(C)CC(NCCc1ccc(cc1)O)=O
N1137	N	-1.662757832	COc1cc(c2C(C=C(c3ccc(c(c3)O)O)Oc2c1)=O)O
N1138	N	-1.67329744	COc1ccc(cc1)C1=CC(c2c(cc(c(c3cc(ccc3O)C3=CC(c4c(cc(cc4O3)OC)O)=O)c2O1)O)O)=O
N1139	N	-1.681241237	C1=C(c2ccc(cc2)O)Oc2c(C3C(c4ccc(cc4)O)Oc4cc(cc(c4C3=O)O)O)c(cc(c2C1=O)O)O
N1140	N	-1.681241237	CC1(C)CCC[C@@]2(C)C1CCC(=C)[C@@H]2CC=C(C=O)C=O
N1141	N	-1.682027185	Cc1cc2C(c3ccccc3C(c2c(c1O)OC)=O)=O
N1142	N	-1.695481677	COc1ccc(cc1)C1=CC(c2c(cc(c(c3cc(ccc3O)C3=CC(c4c(cc(cc4O3)O)O)=O)c2O1)O)O)=O
N6	N	-1.698970004	C[C@@]12CCC[C@]3(C)c4cc5C(C=C(C(c5cc4C([C@]([C@H]23)(O)OC1)=O)=O)OC)=O
N8	N	-1.698970004	C[C@@]12CCC[C@]3(C)c4cc5C(C=CC(c5cc4C([C@]([C@H]23)(O)OC1)=O)=O)=O
N1143	N	-1.698970004	C1Cc2ccc(cc2OC1c1ccc2c(c1)OCO2)O
N114	N	-	COc1cc(c2C(c3c(ccc(c3O)O)Oc2c1)=O)O

7		1.698970004	
N1144	N	-1.698970004	COc1cc(c2C(c3c(ccc(c3O)OC)Oc2c1)=O)O
N1145	N	-1.698970004	COc1cc(c2C(c3c(ccc(c3OC)OC)Oc2c1)=O)O
N1146	N	-1.698970004	COc1ccc(CC(CO)(C(=C)Cc2ccc(cc2)O)O)c(c1)O
N1148	N	-1.705007959	C1=C(c2ccc(c(c2)c2c(cc(c3C(C=C(c4ccc(cc4)O)Oc23)=O)O)O)O)Oc2cc(cc(c2C1=O)O)O
N1149	N	-1.708807013	CC(CC(C=C(C)C)c1ccc2cc[nH]c2c1)c1c[nH]c2cc(CC=C(C)C)ccc12
N1150	N	-1.716003344	CC(C)=CCCC(C)=CCO
N1151	N	-1.718501689	COc1cc2CC3c4cc(c(cc4CCN3Cc2cc1OC)OC)OC
N1152	N	-1.72427587	CN1c2ccccc2C(c2c1c(c(c(c2OC)OC)OC)OC)=O
N1153	N	-1.73239376	CC1C(C)(CC(C2C(C)(C)O2)=O)C2=C(c3c(C)cccc3OC2=O)O1
N1154	N	-1.73239376	Cc1cccc2c1C1=C(C(=O)O2)[C@](C)(CC(C2C(C)(C)O2)=O)[C@H](C)O1
N1155	N	-1.743646091	CC(C)(CCc1c(cc(c2C=CC(=O)Oc12)OC)OC)O
N1156	N	-1.755112266	CN1c2cc3c(c(c2C(c2cccc(c12)OC)=O)OC)OCO3
N1157	N	-1.758154622	c1c(cc(c(c1O)O)O)C1=C(C(c2c(cc(cc2O1)O)O)=O)O
N1158	N	-1.760422483	COc1ccc(C2Cc3ccc4c(cco4)c3OC2)c(c1O)OC
N1159	N	-1.762678564	CN1c2cc3c(c(c2C(c2ccc(c(c12)OC)OC)=O)OC)OCO3
N1160	N	-1.76342799	COc1ccc(c(c1O)OC)[C@H]1Cc2ccc3c(cco3)c2OC1

		4	
N1161	N	-1.767799657	CC(=C)C1CCC2(C)CCC3(C)C(CCC4C5(C)CCC(C(C)(C)C5CCC34C)O)C12
N1162	N	-1.770852012	COc1c2ccccc2nc2c1cco2
N21	N	-1.77815125	CC(C)(CO)C(C(NCCCO)=O)O
N1163	N	-1.780317312	CC(=O)Oc1ccc(cc1OC(C)=O)C1=CC(=O)Oc2cc(cc(c12)OC(C)=O)OC
N1164	N	-1.781454713	C[C@H]1[C@H](C)[C@@H](c2ccc3c(c2)OCO3)O[C@@H]1c1ccc(c(c1)OC)O
N1165	N	-1.781755375	C[C@H]1[C@H](C)[C@@H](c2ccc3c(c2)OCO3)O[C@@H]1c1ccc(c(c1)OC)O
N1166	N	-1.789465383	C1=Cc2ccccc2OC1=O
N1167	N	-1.79669537	CC1(C)C(CC[C@@]2(C)[C@H]1CC[C@]1(C)[C@@H]2CC([C@@H]2[C@@]3(C)CC[C@@]([C@H]3[C@H](C[C@@]12C)O[C@@H]1[C@H]([C@@H]([C@@H](CO1)O)O)O)(C(C)(C)O)O)O)=O
N1168	N	-1.806179974	C1=C(c2ccc(c(c2)O)O)Oc2c(C1=O)c(cc(c2[C@@H]1C(c2c(cc(cc2O[C@H]1c1ccc(cc1)O)O)O)=O)O)O
N1169	N	-1.814807008	CC(C)=CC(CC(C)=C[C@@H]([C@@H]1COC(C1=C)=O)OC(C)=O)=O
N1170	N	-1.8162413	C1=C(c2ccccc2)Oc2cc(ccc2C1=O)O
N1171	N	-1.818885415	C1=C(c2ccccc2)Oc2cccc(c2C1=O)O
N1172	N	-1.819543936	CC(=C)C1CCC(C)=CC1
N1173	N	-1.822168079	CCC1CN2CCc3c4ccccc4[nH]c3C2CC1C(=COC)C(=O)OC
N1174	N	-1.824106108	CN1CCc2cc(c3cc2C1Cc1ccc(c(c1)Oc1ccc(CC2c4c(CCN2C)cc(c(c4O3)O)OC)cc1)OC)OC
N117	N	-	[H][C@@]12CC=CC([C@]2(C)C[C@]2(C(C1)=C(C)C

5		1.828015064	(=O)O2)O)=O
N1176	N	-1.83197113	CC1(C)C(CC[C@@]2(C)[C@H]1CC[C@]1(C)[C@@H]2CC([C@@H]2[C@@]3(C)CC[C@@]([C@H]3[C@H](C[C@@]12C)O[C@@H]1[C@H]([C@@H]([C@@H](CO1)O)O)O)(C(C)(C)O)O)O)=O
N1177	N	-1.835056102	CC(C)C1CCC(C)(C2C1c1c(cc(c(C(CCc3ccccc3)=O)c1O2)O)OC)O
N1178	N	-1.836710514	C[C@@]1(CCC2C1[C@H](OC1C(C(C(C(COC(C=Cc3ccc(cc3)O)=O)O1)O)O)O)O[C@H](C=2C(=O)OC)OC)O
N1179	N	-1.836956737	CC=Cc1ccc(cc1)OC(C(C)C)=O
N1180	N	-1.84509804	CCCc1ccc(cc1)OC(C=C)=O
N1181	N	-1.865103975	COC1=C(c2ccc(c(c2)OC)OC)Oc2cc(cc(c2C1=O)O)OC
N1182	N	-1.866712282	CC(=O)O[C@@H]1C(c2c(cc(cc2O[C@@H]1c1ccc(cc1)O)O)O)=O
N1183	N	-1.869481364	CC(=O)OC1CC(C2(C)c3cc4c(cco4)c(C)c3CCC2(C1(C)C)O)OC(C)=O
N1184	N	-1.874481818	COc1ccc(cc1)C1=CC(c2c(cc(c(c2O)OC)OC)O1)=O
N1185	N	-1.878905064	CC(C)=CC(CC(C)=CC(C1COC(C1=C)=O)O)=O
N1186	N	-1.909020854	COC=C(C1CC2c3c(CCN2CC1C=C)c1ccccc1[nH]3)C(=O)OC
N1187	N	-1.910090546	C(C(C(O)=O)N)c1c[nH]c2ccccc12
N1188	N	-1.911931549	[H]C(C(CC(O)=O)=CC[C@H]1C(=C)CCC2C(C)(C)CCC[C@]12C)=O
N1189	N	-1.962752079	CC1C2CCC3C(C)(CCCC3(C)C(=O)OC)C2Cc2c1cco2
N1190	N	-1.99961911	C1Oc2cc3cc(c4ccc(cc4O)O)oc3cc2O1

		4	
N1191	N	-2.001603924	CC(=O)O[C@H]1[C@H]([C@@]2(C)C3=CC[C@@H](c4ccoc4)[C@]3(C)CC[C@@H]2[C@@]2(C)C(C=C[C@@H]([C@H]12)O)=O)O
N1192	N	-2.005138734	CC1CC2C(CC=C1C(CC(C)=O)OC(C)=O)C(=C)C(=O)O2
N1193	N	-2.00548081	CC(C)C1CCC2(C)CC1c1c(cc(c(C(CCc3ccccc3)=O)c1O2)O)OC
N1194	N	-2.008600172	CN1c2ccccc2C(c2c1c1ccccc1[nH]2)=O
N1195	N	-2.015778756	C1=C(C(c2ccc(cc2O1)O)=O)c1ccc(cc1)O
N1196	N	-2.018453178	CC(C)=CCc1ccc2CC(Nc2c1)=O
N4	N	-2.027349608	C[C@@]12CCC[C@@](C)(C(O)=O)[C@H]1CC[C@@]13CC(=C)[C@H](CC[C@@H]23)C1
N1197	N	-2.042969073	c1cc(C2=C(C(c3c(cc(cc3O2)O)O)=O)O)c(cc1O)O
N1198	N	-2.060320029	CC1=CC(CC2C(C)(CCC3=CCOC3=O)C(CCC12C)CO)=O
N1199	N	-2.064457989	CC1C2=COC=CC2=C(C=O)C(=O)O1
N1200	N	-2.068726419	CC1=C2C(CCN1)=c1ccc(cc1=N2)OC
N1201	N	-2.079181246	COc1cc(c2C(CC(c3ccccc3)Oc2c1)=O)O
N1202	N	-2.087746178	CC1(C)C2CC(C3(C)C(CCC4(C)C(C(C=C34)=O)c3ccoc3)C2(C)C=CC1=O)O
N1203	N	-2.11058971	CC=C1CN2CCC34C2CC1C(CC=O)=C3Nc1ccccc14
N1204	N	-2.136720567	CC(CCC=C(C)CCC(C(C)(C)O)O)=CCCC=C(C)CCC=C(C)CCC(C(C)(COC(C)=O)O)O
N1205	N	-2.13987908	CC=C1CN2C3CC1C1(C2CC2(C3N(C)c3ccccc23)C1O)C(=O)OC

		6	
N1206	N	-2.149219113	C1C(c2ccc(cc2)O)Oc2cc(cc(c2C1=O)O)O
N1207	N	-2.192650338	COc1cc(c2c(c1)c(c1ccoc1n2)OC)OC
N16	N	-2.217483944	C1C2C(c3ccc(cc3O1)O)Oc1cc3c(cc12)OCO3
N1208	N	-2.221468359	COc1ccc(cc1O)C1=COc2cc(cc(c2C1=O)O)O
N1209	N	-2.242517044	CC(=C)C(CCC(C)=O)C=CC1=C(C)C(O)OC1=O
N1210	N	-2.245248936	COc1ccc(cc1)C1=COc2cc(cc(c2C1=O)O)O
N1211	N	-2.255272505	CC(=CCO)C1CC2C(C)(C)C(C=CC2(C)OO1)=O
N1212	N	-2.270407479	COc1ccc(cc1)C1=COc2cc(ccc2C1=O)O
N10	N	-2.301029996	C=CC1C(OC=C2C1=CCOC2=O)OC1C(C(C(C(CO)O1)O)O)O
N1213	N	-2.301029996	COC(C1C2CC3c4c(CCN3CC2CCC1O)c1ccccc1[nH]4)=O
N1214	N	-2.342422681	C=CC1C(OC=C2C1=CCOC2=O)OC1C(C(C(C(CO)O1)O)O)O
N1215	N	-2.344392274	C=CC1C2CCOC(C2=COC1OC1C(C(C(C(CO)O1)O)O)O)=O
N1216	N	-2.359835482	c1ccc2c(c1)cc1c(c3ccccc3[nH]1)n2
N1217	N	-2.40980633	CN1CCc2cc(c3cc2C1Cc1ccc(cc1)Oc1cc(CC2c4c(CCN2C)cc(c(c4O3)OC)OC)ccc1OC)OC
N1218	N	-2.468047733	CN1(C)C[C@@H](C[C@H]1C(O)=O)OC(C=Cc1ccc(cc1)O)=O
N121	N	-	CN1CCc2cc(c3cc2C1Cc1ccc(cc1)Oc1c2C(Cc4ccc(c(

9		2.547774705	c4)O3)O)N(C)CCc2cc(c1O)OC)OC
N1220	N	-2.76902589	C1CO[C@H]2C=C(CO)[C@@H](CO)[C@@H]12
N1221	N	-2.914749791	c1nc2c(C(NC(N2)=O)=O)[nH]1
N1222	N	-2.950364854	CN1CCc2cc(c(c3c2C1Cc1ccc(cc1)Oc1c2C(Cc4ccc(c c4)O3)N(C)CCc2cc(c1O)OC)O)OC
N1223	N	-3.170489336	COc1ccc2CC3c4cc(c(c(c4CCN3Cc2c1OC)O)OC)OC
N1224	N	-3.245062539	COc1c(cc2CCNC3Cc4ccccc4c1c23)O
N1225	N	-3.391014599	COc1cc2CCN3Cc4c(CC3c2cc1OC)ccc(c4OC)O
N1226	N	-3.711160014	COc1ccc2CC3c4c(CCN3Cc2c1OC)cc(c(c4O)OC)OC
N1227	N	-3.716363037	CN1CCc2cc3c(c4c5ccc(cc5CC1c24)OC)OCO3
N1228	N	-3.717928376	COc1cc2CC3c4c(CCN3Cc2cc1OC)cc(c(c4O)OC)OC
N1229	N	-3.78010879	CN1CCc2cc3c(c4c5ccc(cc5CC1c24)O)OCO3
N1230	N	-3.841746335	CN1CCc2cc3c(c4c5cc(c(cc5CC1c24)O)OC)OCO3
N1231	N	-3.855005711	C1CNC2Cc3ccccc3c3c2c1cc1c3OCO1
N1232	N	-3.875847486	CN1CCc2cc3c(c4c5cc(c(cc5CC1c24)OC)OC)OCO3
N1233	N	-3.885714645	COc1ccc2c(CC3c4c(CCN3)cc3c(c24)OCO3)c1
N1234	N	-3.947030384	CN1CCc2cc3c(c4c5cc(c(cc5CC1c24)OC)O)OCO3
N123	N	-	COc1cc2C=CC(=O)Oc2c(c1O)OC

5		4.553634592	
