# Supplementary material for: Predictive classifier models built from natural products with antimalarial bioactivity using machine learning approach
Source: PLoS One. 2018 Sep 28;13(9):e0204644. doi: 10.1371/journal.pone.0204644 (PMC6161899; doi:10.1371/journal.pone.0204644)
Supplement: S2 Table — (PDF) [file pone.0204644.s002.pdf]

S2 Table. The antiplasmodial bioactivity class predictions of 450 natural compounds from a private natural product chemical library from InterBioScreen (http://www.ibscreen.com) by Sequential Minimization Optimisation (SMO) and Random Forest (RF) classifier models.

Molecule Name	Smiles	Class prediction SMO	Class prediction RF
STOCK1N-00001	C[N+](CC1)(Cc(c([C@H]2[C@H]3O)c4)cc5c4OCO5)[C@H]2C1=C[C@H]3O.[I-]	A	A
STOCK1N-00002	CC(N[C@@H](CCc(c(C1=CC=C2NCc(cc3)ccc3F)c3OC)cc(OC)c3OC)C1=CC2=O)=O	N	A
STOCK1N-00004	COc(cc(CC1(CCCCC1)N=C1)c1c1)c1OC.Cl	N	A
STOCK1N-00006	CC(C1Oc(cccc2)c2OC1)NC(c(cc1)ccc1OCC=C)=O	N	A
STOCK1N-00007	COc1cc(CN(C/C=C/c2ccccc2)CC23CCOCC3)c2cc1OC.Cl	N	A
STOCK1N-00010	CCOC(CCCCCCCCCCc1ncccc1)=O	N	N
STOCK1N-00013	CCCCCCCCCO[C@H]([C@H]([C@@H]1OC(C)=O)NC(C)=O)O[C@@H](COC(C)=O)[C@@H]1OC(C)=O	A	A
STOCK1N-00022	C[C@@H]1[C@@]2(COC(NC3CCCC3)=O)C(C)C=C(C)C1C(c1ccco1)OC2	N	A
STOCK1N-00024	CC(C1Oc(cccc2)c2OC1)NCC1Oc(cccc2)c2OC1.CC(C1Oc(cccc2)c2OC1)NCC1Oc(cccc2)c2OC1.OC(C(O)=O)=O	A	A
STOCK1N-00026	N=C1N=C(c2c(C3)cccc2)N3Cc2c1cccc2.Cl	N	N
STOCK1N-00028	CC1(C)c(cccc2)c2N(C2)[C@@]1(/C=C/c(cc1)ccc1N(C)C)NC2=O	A	A
STOCK1N-00030	CCOc(cc1)ccc1OC1=COc(cc(cc2OC(C)=O)OC(C)=O)c2C1=O	A	A
STOCK1N-00031	CC(C)C[C@@H](C(NCC(O)=O)=O)NC(C(CC1)N(C(OCc2ccccc2)=O)C1=O)=O	A	A

STOCK1N-00032	Cc1nc(C2=COc(cc(c(C)c3)OCC(OC)=O)c3C2=O)cs1	A	A
STOCK1N-00033	C[C@@H](CCC=C(C)C)NCCCN(C)C	N	A
STOCK1N-00034	CC(C(C(O)=O)NC(c1cnccc1)=O)O.Cl	N	A
STOCK1N-00036	CC1(C)Oc2c(C)ncc(CCl)c2CO1.Cl	N	N
STOCK1N-00039	CCCc(c(OC(C)C)c1)cc2c1OC=C(c(cc1)cc3c1OCCO3)C2=O	A	A
STOCK1N-00044	CCOC([C@H](Cc1c[nH]c2c1cccc2)NC(CCC(OC)=O)=O)=O	A	A
STOCK1N-00047	CC(C)CC(C(NCC(OC(C)(C)C)=O)=O)NC(C(CC(N)=O)NC(C(Cc1c[nH]cn1)N)=O)=O	A	A
STOCK1N-00048	C[C@H](C(Oc(cc1)cc(OC(C)=C2c(cc3)cc4c3OCCCO4)c1C2=O)=O)N.Br	A	A
STOCK1N-00051	CC(CC(CCC=C(C)C)=CC1)C1C(OC1)OCC11COC(C2C(C)CC(CCC=C(C)C)=CC2)OC1	N	A
STOCK1N-00054	C(CC1)CC11OC(CCCC2)[C@H]2C2=CCCCC12	N	A
STOCK1N-00055	C[C@H](CCC([C@]1(C)COC(c2ccccc2)=O)[C@]2(C)CC[C@@H]1OC(c1ccccc1)=O)[C@@]12O[C@@](CCOC(c2ccccc2)=O)(COC(c2ccccc2)=O)CC1	A	A
STOCK1N-00062	CCCOc1ccc(CC2=N[C@@](C)(CO)CO2)cc1	N	A
STOCK1N-00064	CC(C)(C1CC2CC1)[C@@]2(C)c(cccc1)c1O	N	A
STOCK1N-00067	CCCc(c(OC(C)=O)c1)cc2c1OC=C(c1cc(cccc3)c3o1)C2=O	N	N
STOCK1N-00068	CC1(C)OC(C)(C)O[C@H]1COc1ccc(c(OC)c(cco2)c2n2)c2c1OC	A	A
STOCK1N-00070	CCCCSCC(CN(C[C@@H](C1)CN23)C[C@H]1C3=CC=CC2=O)O	A	A
STOCK1N-00074	CC(Nc(cc1)cc(O2)c1C(C)=CC2=O)=O	N	A
STOCK1N-00075	CCCC(C1)C[N+]2(C)[C@]1(C)C(CCC1)=C1CC2.[I-]	A	A
STOCK1N-00080	[O-][N+](c1ccc(/C=C(\CCN23)/C3=Nc(cccc3)c3C2=O)cc1)=O	A	A
STOCK1N-00081	CC(C)OC(CC(C(Nc1c2cccc1)=O)=C2O)=O	N	A

STOCK1N-00082	CC([C@H]1C(N2CCC[N+](C)(C)C)=O)C=C(C)C[C@@H]1C2=O.[I-]	A	A
STOCK1N-00090	CC(N[C@@H](CCc(c(C1=CC=C2NCc(cc3OC)cc(OC)c3OC)c3OC)cc(OC)c3OC)C1=CC2=O)=O	N	A
STOCK1N-00093	N[C@@H](CC(O)=O)c1ccccc1	N	A
STOCK1N-00095	C[C@@](CC[C@@H]([C@H]1CC2)[C@@](C)(CC3)[C@H]2C[C@H]3OC(C)=O)([C@H](CC2)C(CO3)=CC3=O)[C@@]12O	A	A
STOCK1N-00096	CN(CCC1)[C@@H]1c1cccnc1N.I	N	A
STOCK1N-00099	CCCC(Oc1c2cc(CC)c(OC(C)C)c1)=C(c(cc1)cc3c1OCCCO3)C2=O	A	A
STOCK1N-00101	CCOC(/C(/C#N)=C(/CC1)\[C@@](C)(CC2)C1[C@H]1[C@H]2[C@@](C)(CCCC2)C2CC1)=O	N	A
STOCK1N-00102	CCOc(cc1)cc(OC(C)=C2Oc(cc3)cc4c3OCCCO4)c1C2=O	N	A
STOCK1N-00103	CCOC(C(C)Oc(cc1OC(C)C(OCC)=O)cc(O2)c1C(c1ccccc1)=CC2=O)=O	A	A
STOCK1N-00105	Oc(cc1)cc(O)c1C(COc(cccc1)c1Br)=O	N	A
STOCK1N-00106	CC([C@]1(CCC2)C2C2CC1CC2)N.Cl	N	A
STOCK1N-00108	NC(C(N1)=O)=C(C(O)=O)NC1=O	N	A
STOCK1N-00113	NC(COc(cc1)cc(O2)c1C(CCC1)=C1C2=O)=O	N	A
STOCK1N-00116	CC(CCC=C(C)C)N(C(C)CCC=C(C)C)C=O	N	A
STOCK1N-00117	CCCCOC(COc(c(CC)c1)cc(O2)c1C(C)=CC2=O)=O	N	A
STOCK1N-00124	CC(C)COc1ccc([C@H](C(N2)=O)NC2=O)cc1	N	A
STOCK1N-00126	C[C@@](CN(C)CC([C@@H](C[C@H]([C@](C)(CCC1)C2)C1=C)[C@@H]2O1)C1=O)(c1ccccc1)O	N	A
STOCK1N-00127	C[C@@H](C(O)=O)Oc(cc1)cc(OC(C)=C2Oc3ccccc3)c1C2=O	A	A
STOCK1N-00130	C/C(/CCc(c(C)c1)c(C)c(C)c1OC)=C\C=C\C(C)=O	N	N
STOCK1N-00132	CC#CCCCCCCCC(O)=O	N	A
STOCK1N-00133	CC(C)c(cc1)ccc1OC1=C(C)Oc(cc(cc2)OC(C)=O)c2C1=O	A	A
STOCK1N-00139	C[C@@H](CC=C(c1ccccc1)c1ccccc	A	A

	1)[C@H](CC1)[C@](C)(CC2)[C@H]1[C@@H]1[C@@H]2[C@](C)(CC[C@H](C2)O)C2=CC1		
STOCK1N-00141	OC(C(CC(C1C(O)=O)C(O)=O)C1C(O)=O)=O	N	A
STOCK1N-00145	C[N+](C)(C[C@H]1C2)C(CC=C)C1=Cc1c2cccc1.[Br-]	A	N
STOCK1N-00146	CC(C)(C1)OCC[C@]1(CCO)O	N	A
STOCK1N-00147	CC(C)[C@@H](C(OC)=O)NC(COc(cc1)cc(OC=C2Oc3ccccc3)c1C2=O)=O	A	A
STOCK1N-00148	CC(OC(N)=C([C@@]1(c(cccc2)c2N2)C2=O)C(OC)=O)=C1C(OCC=C)=O	N	A
STOCK1N-00150	CCOC(C(C1)C2(CC(C)(C)OCC2)OC1=O)=O	N	A
STOCK1N-00151	CC(C)(C(CC1)C2OC1(C)C#N)Oc1c2cccc1	A	A
STOCK1N-00154	CCCOc(cc1)ccc1C(OC[C@@H]1[C@H](CCCC2)[N+]2(C)CCC1)=O.[I-]	A	A
STOCK1N-00156	CN(C(SCC(CS[C@H]1[C@H]2N)=C(C(O)=O)N1C2=O)=NC1=O)NC1=O	A	A
STOCK1N-00157	CC(C)(CC(C)=C1)[C@@H](C(C)=O)C1=O	N	A
STOCK1N-00159	CCc(c(O)c1)cc2c1OC(C(O)=O)=C(c1nc(C)cs1)C2=O	A	N
STOCK1N-00162	CC(C)(C)OC(N[C@@H](Cc1ccccc1)C(Oc(cc1)cc(OC(C)=C2c(cc3)cc4c3OCCCO4)c1C2=O)=O)=O	A	A
STOCK1N-00165	C[C@](CCC1)(C[C@@H]([C@@H](C2)C3CN4CCC(Cc5ccccc5)CC4)OC3=O)[C@H]2C1=C	A	A
STOCK1N-00166	COc(cc1)cc(OC(c(cc2)cc3c2OCCCCO3)=C2)c1C2=O	N	A
STOCK1N-00167	O=Cc1ccc(SCCSc2ccc(C=O)s2)s1	A	A
STOCK1N-00169	C(c1ccccc1)=C/c1cccc(/C=C/c2ccccc2)n1	N	N
STOCK1N-00175	C[C@@H](CCc1ccccc1)CCO	N	A
STOCK1N-00178	Cc1c(C(c2cc(cc(cc3)OC)c3o2)=O)c(cc(cc2)OC)c2o1	N	A
STOCK1N-00179	C[C@@H](CCc(c(OC)c1)cc(OC)c1OC)N.Cl	N	A
STOCK1N-00181	CC(C)(Cc1c-2cccc1)n1c2nnc1-c1cccnc1	N	A

STOCK1N-00185	CCc(cc1)ccc1OC1=COc(cc(cc2)O)c2C1=O	N	A
STOCK1N-00188	CC(C1)N(Cc2c3cccc2)C3c2c1c(cccc1)c1[nH]2.OC(CC(O)=O)(CC(O)=O)C(O)=O	A	A
STOCK1N-00192	COC(CCN(CC1)Cc(cc2OC)c1cc2OC)=O.Cl	N	A
STOCK1N-00193	CC(O[C@H](CO[C@@H]([C@@H]1OC(C)=O)N(C(N2)=S)N=CC2=O)[C@H]1OC(C)=O)=O	A	A
STOCK1N-00197	OC(COc(cc1)cc(OC=C2c3cscn3)c1C2=O)=O	A	N
STOCK1N-00199	Oc(cc1O)cc(OC=C2Oc(cc3)ccc3F)c1C2=O	N	N
STOCK1N-00205	C[C@](CC1)([C@@](C)(CC2)C1[C@H](CC1)C2[C@@](C)(C=C2)C1=CC2=O)O	N	A
STOCK1N-00209	CC(c(ccc(OCCCCl)c1)c1O1)=CC1=O	N	A
STOCK1N-00210	OC([C@H](C1)NCc2c1cccc2)=O.Cl	N	A
STOCK1N-00212	C[C@@H](C(O)=O)NCc(c(O1)c(cc2)C(CCC3)=C3C1=O)c2O	N	A
STOCK1N-00215	CC(C)(C)OC(N[C@@H](Cc(cc1)ccc1OC(OC(C)(C)C)=O)C(NCC(OC)=O)=O)=O	N	A
STOCK1N-00217	CC(C)c1c(cc(C)c(-c(c(C)cc(c2c3/C=N/Cc(cccc4)c4O)c(C(C)C)c(O)c3O)c2O)c2O)c2c(/C=N/Cc(cccc2)c2O)c(O)c1O	A	A
STOCK1N-00221	CCCCC(C)(C(NC(C1SC(C)(C)C(C([O-])=O)N11)C1=O)=O)OC(CC(C)CC1)C1C(C)C.[Na+]	A	A
STOCK1N-00222	CC(C)(C)OC(CNC(CNC([C@@H](Cc(cc1)ccc1O)NC(OC(C)(C)C)=O)=O)=O)=O	A	A
STOCK1N-00225	O=C1N(CC/C2=C\c3ccccc3)/C2=Nc2c1cccc2	A	A
STOCK1N-00228	CC(CCc(oc1c2cc(C)cc1)c2-c1ccc(C)cc1)=O	N	A
STOCK1N-00229	CCCCCC(C(C)C(C(OCC#C)=O)O1)C1=O	A	A
STOCK1N-00237	CC(C)(C1)OCC[C@]1(CC1)C=CC1=O	N	A
STOCK1N-00239	C1C[C@@H](c2cccnc2)NCC1.Cl	N	N
STOCK1N-00242	CCc(c(OC)c1)cc(C(Cc(cc2)cc3c2OC	N	A

	CCO3)=O)c1O		
STOCK1N-00243	C1c2cc3ccccc3nc2-c2c1cccc2	N	N
STOCK1N-00244	C[C@@H](CO)NCCn1c(C(N(C)C(N2C)=O)=O)c2nc1	N	A
STOCK1N-00249	CC1(C)Cc2nc(C)c3[nH]c(cccc4)c4c3c2CC1	N	A
STOCK1N-00252	CCCOc1cc(C)cc(O2)c1C(c1ccccc1)=CC2=O	N	A
STOCK1N-00253	O=C(COc(cc1)cc(OC=C2Oc3ccccc3)c1C2=O)ON(C(CC1)=O)C1=O	A	A
STOCK1N-00256	CC([C@@H]1C2)=CC[C@@]2(CO)COC1c(cccc1)c1O	N	A
STOCK1N-00258	OCCNc1ncnc2c1nc[nH]2	N	A
STOCK1N-00259	CC(C)(Cc(cc(c(OC)c1)OC)c1C1=C/C2=N/O)N1/C2=O	N	A
STOCK1N-00262	CC1(CC(OC)=O)OCC2(CC=C(C)CC2)CO1	N	A
STOCK1N-00263	O=C(c1/c2cccc1)O/C2=C\C=C\c1ccccc1	N	N
STOCK1N-00265	CN(C)CC(Nc1c(CCC2)c2nc2ccccc12)=O.OC(C(O)=O)=O	A	A
STOCK1N-00266	CC(C)(C1)CC(NC(C)=C([C@@H]2c3ncccc3)C(OC)=O)=C2C1=O	N	A
STOCK1N-00268	C[C@@]1(CCC=C(C)C)N(C=O)N=C(CCC=C(C)C)C1	A	N
STOCK1N-00270	NC(CN(CCCC1)C1=O)=O	N	A
STOCK1N-00271	CN(CCCC1)[C@@H]1c1cccnc1N.Cl	N	A
STOCK1N-00272	CC(C)=CCCC(CC1C(O)=O)=CCC1C(O)=O	N	A
STOCK1N-00273	CC(C)COc(cc1)ccc1C(N/C(/C(Nc(cc1)ccc1OC)=O)=C/c1ccccc1)=O	A	A
STOCK1N-00274	C[C@](CC1)([C@@](C)(CC2O)C1C(CC[C@@H](C1)[C@]3(C)CCC1=O)[C@@]23F)O	N	A
STOCK1N-00277	CCc1nc2ccccc2c(NC(CN2CCC(C)CC2)=O)c1C.OC(C(O)=O)=O	N	A
STOCK1N-00280	C[C@@](CC1)(C(CC2)[C@@H](C3)C1[C@](C)(CCC(C1)=O)[C@@H]1[C@@H]3O)[C@H]2OC(C)=O	N	A
STOCK1N-00282	COC(c(cc(c(OC)c1)OC)c1NC(c(cc1OC)cc(OC)c1OC)=O)=O	N	A
STOCK1N-00284	Cc(cc1)cc2c1OC(c1c3OCOCc3cc(Cl)c1)=CC2=O	N	N

STOCK1N-00285	CCCCCCCC[N+](C)(C)CCOc(cc1)cc(O2)c1C(C)=CC2=O.[Br-]	A	A
STOCK1N-00286	CC(C)(C)OC(N[C@@H](Cc1ccccc1)C(Oc(cc1)cc(O2)c1C(C)=CC2=O)=O)=O	A	A
STOCK1N-00288	CCOc1cc2nc(NC(N)=N)nc(C)c2cc1	N	A
STOCK1N-00289	O=C(CCC(N(C[C@@H](C1)CN23)C[C@H]1C3=CC=CC2=O)=O)NC1C(Cc2ccco2)CCCC1	A	A
STOCK1N-00290	CCCc1nc(C(CC(C)(C)C2)=O)c2o1	N	A
STOCK1N-00291	CCOC1=CC2=N[C@@](c(cccc3)c3N3C)(C3=O)N=C2C=C1	N	A
STOCK1N-00292	CC(Oc(cc1O)cc(OC(c(cc2)cc(OC(C)=O)c2OC(C)=O)=C2OC(C)=O)c1C2=O)=O	A	A
STOCK1N-00296	CCCCOc(cc1OCCCC)cc(OC(c2ccccc2)=C2)c1C2=O	N	N
STOCK1N-00298	CCC(C(C)/N1)=C(C)/C1=C/c1c(C)c(C)c(C)[nH]1.Br	N	A
STOCK1N-00299	CC(Oc(cc1)cc(OC=C2c3cc(cccc4)c4o3)c1C2=O)=O	A	A
STOCK1N-00300	CN(C)CCCCCN(C)Cc1cc(cccc2)c2o1.Cl	N	A
STOCK1N-00301	CC(C)=CCOc(cc1O)cc(OC(c2ccccc2)=C2)c1C2=O	N	A
STOCK1N-00305	COc(cc1)cc2c1[nH]cc2CCNC(c(cccc1)c1Cl)=O	N	N
STOCK1N-00308	CCCc(c(OC)c1)cc2c1OC(C(OCC)=O)=C(c1ccc(C(OCC)=O)o1)C2=O	A	A
STOCK1N-00309	CN(C)C(CN(C([C@@H](CC1)[C@H]2c3c1[nH]c1c3cccc1)=O)C2=O)=O	A	A
STOCK1N-00310	CC(NCCCC(N[C@@H](CCC(OCc1ccccc1)=O)C(OCc1ccccc1)=O)=O)=O	A	A
STOCK1N-00311	CC(c(ccc(/N=C/c1cnccc1)c1)c1O1)=CC1=O	N	A
STOCK1N-00315	C[C@@H]([C@@H]([C@H](C1)O2)[C@@](C)(CC3)[C@@H]1[C@H](CC1C)[C@H]3[C@@](C)(CC[C@H](C3)O)[C@]13O)[C@]12OCC(C)CC1	N	A
STOCK1N-00316	CC(C)(CCC1)C(CC2C(N3)=O)=C1CC2C3=O	N	A
STOCK1N-00320	OC[C@H]([C@H]([C@H]([C@H]1O)O)O)O[C@H]1SC(C(N1)=O)=NNC1	A	A

	=O		
STOCK1N-00322	Cc([nH]nc1-c(ccc(OC)c2)c2O)c1-c(cc1)cc2c1OCCCO2	N	N
STOCK1N-00323	Oc(cc1)cc(OC(C(F)(F)F)=C2c3cc(cccc4)c4o3)c1C2=O	N	A
STOCK1N-00324	O=C([C@@H]([C@H]([C@@H]1O[C@@H]2OC1)[C@H]([C@@H]13)C2=O)N1C=Cc1c3cccc1)C1(CC(C2)C3)CC3CC2C1	N	A
STOCK1N-00326	CN(C)CCCN1C(c2ccccc2)OC(COc2ccccc2)C1	N	A
STOCK1N-00328	COc1ncnc2c1[nH]c1c2cccc1	N	A
STOCK1N-00329	CC1(C)c(cccc2)c2N(C2)[C@@]1(/C=C/c1ccccc1)NC2=O	A	N
STOCK1N-00330	Cc1c(/C=C/C(N2)=Nc(cccc3)c3C2=O)c(cc(cc2)OC)c2n1C	A	A
STOCK1N-00331	Nc1ncnc2c1nc(CO)[nH]2.O	N	A
STOCK1N-00336	Oc1cc(-c(c(O)cc(O)c2)c2O)cc(O)c1.O	N	A
STOCK1N-00337	CCc(cc1)ccc1C(C[C@H]1c(c2c3OC)ccc3OC)=Nc(cccc3)c3N1C2=O	A	A
STOCK1N-00338	CCCCCCCCNc(c1cc(C)cc(C)c1nc1CC(C)(C)C2)c1C2=O.Cl	A	A
STOCK1N-00340	Cn1c(C(N(C)CC(N2C)=O)=O)c2nc1	N	A
STOCK1N-00347	Cc(cc(c1c2)Nc(cc3)cc(O)c3C(O)=O)nc1ccc2OC	N	A
STOCK1N-00348	CCOC([C@H](C)Oc(cc1)c(C)c(OC=C2c3cc(cccc4)c4o3)c1C2=O)=O	A	A
STOCK1N-00349	CC(C)[C@@H](C(Oc(cc1)cc(OC(C)=C2Oc(cc3)ccc3Cl)c1C2=O)=O)NC(OCc1ccccc1)=O	A	A
STOCK1N-00350	CC(C)(C)OC(NC1(CCCCC1)C(N[C@@H](CCSC)C(OC)=O)=O)=O	N	A
STOCK1N-00351	CC(C)(C)OC(CC(C(NC(CCC(N)=O)C(O)=O)=O)NC(OCc1ccccc1)=O)=O	A	A
STOCK1N-00352	COC(c(cc1)ccc1N[C@H](c(c1c2OC)ccc2OC)OC1=O)=O	N	A
STOCK1N-00355	CCc(c(OC)c1)cc(-c(nc(N)nc2)c2-c(cc2)cc3c2OCCO3)c1O	A	A
STOCK1N-00357	CC(C)COc(cc1)ccc1/C(/O)=N/C(/C(NCCCCCC(O)=O)=O)=C/c1ccccc1	A	A
STOCK1N-00359	CCCc(c(OC(C)=O)c1)cc2c1OC=C(c1ccc(C(OCC)=O)o1)C2=O	A	A

STOCK1N-00361	COc1cc([C@H](c(cc2)ccc2[N+]([O-])=O)NCC23CCCC3)c2cc1OC.Cl	A	A
STOCK1N-00364	CCCC(CCC)(/C(/NC(N1)=O)=N/CCc(cc2)cc(OC)c2OC)C1=O	A	A
STOCK1N-00368	Oc1ccc(CCC(N(C[C@@H](C2)CN34)C[C@H]2C4=CC=CC3=O)=O)cc1	A	A
STOCK1N-00370	COC(c(cc1)ccc1OC1=COc(cc(cc2)OC([C@H](Cc3c[nH]c4c3cccc4)NC(OCc3ccccc3)=O)=O)c2C1=O)=O	A	A
STOCK1N-00371	CCCC(CC[C@]1(C)N2CCCc3c1[nH]c1c3cccc1)C2=O	N	A
STOCK1N-00373	Oc(cc1)cc(OC=C2Oc(cc3)ccc3Cl)c1C2=O	N	A
STOCK1N-00374	CCCCCCCCC1OC1CCCCC(C)C	N	N
STOCK1N-00377	Cn1ncc(Nc2c3cccc2OC)c1C3=O	A	A
STOCK1N-00379	CC(CC1)CN(CC([C@@H]2C3)C4CC[C@H]2C(C2)[C@H]3[C@@](C)(CCC(C3)OC(c5ccco5)=O)[C@H]3C2=O)[C@@H]1[C@@]4(C)O	A	A
STOCK1N-00382	O[C@@H](C1)CN=C2N1C=CC=C2.Cl	N	A
STOCK1N-00383	COc(ccc(C[C@@H](C(O)=O)c(cc1)cc(OC)c1OC)c1)c1OC	N	A
STOCK1N-00384	C[C@](CCc1c(-c2ccc(C)o2)c(cc(C)cc2)c2o1)(c1ccccc1)O	N	A
STOCK1N-00387	OC(CCCNc(cccc1)c1C(O)=O)=O	N	A
STOCK1N-00388	CC(C)(C)OC(NCC(Oc(cc1)cc(O2)c1C(C)=CC2=O)=O)=O	N	A
STOCK1N-00392	CC(C)CC1=C(C)c(ccc(OCC=C)c2)c2OC1=O	N	N
STOCK1N-00396	CCOC(C([C@@H]1c2ccco2)=C(N)OC(c(cccc2)c2O2)=C1C2=O)=O	N	A
STOCK1N-00400	CC(C)(CC(C1C(C(C(CC(C)(C)C2)=O)C2=O)c(cc2)ccc2OCC(OC)=O)=O)CC1=O	N	A
STOCK1N-00402	C=CC1OC(CCCC2)C2C2=CCCCC12	N	A
STOCK1N-00405	C[C@@H](Cc1c[nH]c2c1cccc2)NC	N	A
STOCK1N-00407	CCOc1ccc2nc(NC(N3)=NCC3=O)nc(C)c2c1	A	A
STOCK1N-00409	Oc(cc1O)cc(OC=C2c3ncccc3)c1C2=O	N	N
STOCK1N-00411	CCCCc(c(OC)c1)cc2c1OC=C(c1cccc	N	A

	c1)C2=O		
STOCK1N-00414	CCOc(cc1)ccc1OC1=C(C)Oc(cc(cc2)OCc3cc(Oc4ccccc4)ccc3)c2C1=O	A	A
STOCK1N-00416	C[C@@H](C(NCC(NCC(N)=O)=O)=O)NC(C(Cc(cc1)ccc1OC(OC(C)(C)C)=O)NC(OC(C)(C)C)=O)=O	A	A
STOCK1N-00418	COc(cc1OC)cc(OC(c(cc2)cc(OC)c2OC)=C2OC)c1C2=O	A	N
STOCK1N-00419	OC(CC(C(Nc1c2cccc1)=O)=C2O)=O	N	A
STOCK1N-00422	COC([C@H](Cc1ccccc1)NC(COc(cc1O)cc(OC=C2c(cc3)cc4c3OCCO4)c1C2=O)=O)=O	A	A
STOCK1N-00423	C/C(/CCC1=C(C)CC[C@H]1C(C)=C)=N\NC(N)=S	N	A
STOCK1N-00425	CC(C)(Cc1c2cccc1)N=C2N[C@@H](Cc1c[nH]c2c1cccc2)C(O)=O	A	A
STOCK1N-00426	Cc1ccc(C(CN2)C(C(C[N+]([O-])=O)c3ccco3)(C(OC)=O)C2=O)cc1	A	A
STOCK1N-00427	CCC(C)[C@@H](C(O)=O)NCc(c(OC=C1Oc(ccc(Cl)c2)c2Cl)c(cc2)C1=O)c2O	A	A
STOCK1N-00429	CCCN(C([C@H](Cc1c2[nH]c3c1cccc3)N1C2c(cc2OC)cc(OC)c2OC)=O)C1=O	A	A
STOCK1N-00434	CC1[C@]2(COC(C)=O)COC[C@H]1C1=CCCC(C)(C)C1C2	N	A
STOCK1N-00436	Oc1cc(OC([C@H](CCC(OCc2ccccc2)=O)NC(OCc2ccccc2)=O)=O)cc(OC(c2ccccc2)=C2)c1C2=O	A	A
STOCK1N-00439	CC(C)(C/C=C(\C)/CCC=C(C)C)[C@@H]1OCCN1C(C)=O	N	N
STOCK1N-00442	CC(C)COc(cccc1)c1C(NCCC(O)=O)=O	N	A
STOCK1N-00446	Cc(cc1OC(CCNC(OCc2ccccc2)=O)=O)cc(O2)c1C(c1ccccc1)=CC2=O	A	A
STOCK1N-00450	CC(c(ccc(OCC(N[C@@H](Cc1ccccc1)C(O)=O)=O)c1)c1O1)=CC1=O	A	A
STOCK1N-00453	CCCCN(CCCC)CCCC.Nc1c2ncn([C@H]([C@H]3O)O[C@@H](COP(O)(O)=O)[C@@H]3O)c2ncn1	A	A
STOCK1N-00454	Cc(cc1)c(C)cc1N(CC(C(C(CO)O)O)O)N=O	N	A
STOCK1N-00456	CCCCOc(cc1)ccc1C(N[C@@H](CC(	N	A

	C)C)C(O)=O)=O		
STOCK1N-00458	CC1(C)Cc2nc(C)c3n(CCCCCl)c(cccc4)c4c3c2CC1	A	A
STOCK1N-00459	OC(C(O)=O)=O.Oc1ccc(CCNCC(Nc2c(CCC3)c3nc3ccccc23)=O)cc1	A	A
STOCK1N-00460	CC(C)=CCC/C(/C)=C/CCCOC(C)=O	N	N
STOCK1N-00461	CCN(C(/C=C/c(cccc1OC)c1O)=Nc1c2cccc1)C2=O	A	N
STOCK1N-00462	CCCCCCC(C(CC(OC)=O)CC1)C1=O	N	A
STOCK1N-00464	CNC(N(C[C@@H](C1)CN23)C[C@H]1C3=CC=CC2=O)=O	N	A
STOCK1N-00466	OCC(OC=C1O)=CC1=O	N	A
STOCK1N-00467	OC(c1cc(C(O)=O)c([C@@H](C2)[C@@H]2c2ccccc2)cc1)=O	N	N
STOCK1N-00469	CC(C)CC1OC2c(cccc3)c3OC(C)(C)C2C1	N	A
STOCK1N-00470	C[C@@H](CCc(ccc(OC)c1)c1OC)NC(CCc1ccccc1)=O	N	N
STOCK1N-00471	C[C@H](CC([C@H](CC1)C2[C@@](C)(CC3)C1CC3=O)[C@]1(C)C[C@H]2O)[C@@H]1C(C)=O	N	A
STOCK1N-00474	CCOC([C@@H]1[C@H](CCCC2)[N+]2(C)CCC1)=O.[I-]	A	A
STOCK1N-00475	CC(C)CCC[C@@H](C)[C@@H](CC1)[C@@](C)(CC2)[C@@H]1[C@H](CC1)[C@H]2[C@]2(C)[C@@H]1CC(OC(C)=O)=CC2	N	A
STOCK1N-00476	O=C(/C=C/C=C/c1ccccc1)C1=Cc(cccc2)c2OC1=O	N	A
STOCK1N-00481	C=CCN(C([C@H](Cc1c2[nH]c3c1cccc3)N1C2c(cc2)ccc2O)=O)C1=S	A	A
STOCK1N-00483	COc1ccc([C@H](CC(C2CC2)=O)OC2=O)c2c1OC	A	A
STOCK1N-00484	OC(CCc1c[nH]c2c1cccc2)=O	N	A
STOCK1N-00491	CCc1c(C)[nH]c(/C=C2\N=C(C)C(CC)=C2C)c1C.Br	N	N
STOCK1N-00494	O=C1OC(c2cccs2)=CC=C1	N	A
STOCK1N-00496	CC(NC(N1)=O)=C(CO)C1=O	N	A
STOCK1N-00497	COc(ccc(C1(CNC[C@@H](c(cc2)cc(C(N)=O)c2OCc2ccccc2)O)CCCC1)c1)c1OC.Cl	A	A
STOCK1N-00499	Oc(cc(c(C1=C2CCC1)c1)OC2=O)c1Cl	N	A

STOCK1N-00501	Cc(cc1)cc2c1n1c3c2CCC[C@@H]3N(Cc2ccccc2)CC1	A	A
STOCK1N-00504	CC1C(CNCCCN(C)C)CC(C(C)(C)CCC2)=C2C1	N	A
STOCK1N-00505	O=C1c(cccc2)c2OC(c(cc2)cc3c2OCCCO3)=C1	N	A
STOCK1N-00508	CN(C[C@H](C1)CN23)C[C@H]1C3=CC=CC2=O	N	A
STOCK1N-00510	CCc(c(OC(C)=O)c1)cc2c1OC(C)=C(c(cc1)cc3c1OCCO3)C2=O	A	A
STOCK1N-00511	CC(COc1cc(OC(C=C2C)=O)c2c(OCC(C)=C)c1)=C	N	A
STOCK1N-00512	Cc1nc(Cc(cc2)cc(OC)c2OC)c(cc(c(OC)c2)OC)c2c1	N	N
STOCK1N-00514	CC(C)(C)OC(N[C@@H](Cc1c[nH]c2c1cccc2)C(Oc(cc1)cc(OC=C2Oc3ccccc3)c1C2=O)=O)=O	A	A
STOCK1N-00519	CC(CC1=Cc(cccc2)c2OC1=O)=O	N	A
STOCK1N-00521	OC(COc(cc1)cc(O2)c1C(c1ccccc1)=CC2=O)=O	N	A
STOCK1N-00523	CCCCNCc(cc1)cc(OC)c1OC	N	N
STOCK1N-00526	COC(C(c(cccc1)c1C1=O)=O)=C1O	N	N
STOCK1N-00533	CC(Nc(cc1)cc(NC([C@H]2O)O[C@@H](C(CO)O)[C@H]2O)c1NC(C)=O)=O	N	A
STOCK1N-00538	COc1cc(COCC23CCCC3)c2cc1OC	N	A
STOCK1N-00540	C[C@](CCc1c-2c(cccc3)c3o1)(c1c2oc(C)c1)c1ccccc1	N	A
STOCK1N-00541	CCCCc(c(C)nc1ccccc11)c1O	N	A
STOCK1N-00543	CCCCOC(COc(cc1OCC(OCCCC)=O)cc(O2)c1C(c1ccccc1)=CC2=O)=O	A	A
STOCK1N-00545	C1CC2C3=CCCCC3C(c3ccccc3)OC2CC1	N	A
STOCK1N-00550	Cc(cc1)cc(OC(c(cc2)cc3c2OCCO3)/C2)c1/C2=N/Nc1ccccc1	N	A
STOCK1N-00552	CCc(c(OC(C)C)c1)cc2c1OC(C)=C(c(cc1)cc3c1OCCO3)C2=O	A	A
STOCK1N-00557	CN(C=Nc1c2[nH]c3c1cccc3)C2=O	N	A
STOCK1N-00561	COc1ccc([C@@H](N2)Oc(cccc3)c3C2=O)cc1	N	N
STOCK1N-00562	CCCc(c(OC)c1)cc2c1OC=C(c1ccc(C(OCC)=O)o1)C2=O	A	N

STOCK1N-00565	CC1C=C(C)C2C(c3ccccc3)OCC1[C@@H]2COC(CCl)=O	N	A
STOCK1N-00567	Cc1c(C(c2c(C)[nH]c3c2cccc3)c(cc2)ccc2OC)c(cccc2)c2[nH]1	N	A
STOCK1N-00569	CCC(OC[C@@]1(C2)C(C)C=C(C)[C@H]2C(C)OC1)=O	N	A
STOCK1N-00570	Cc1c(C(CN(C)C)=O)c(cccc2)c2[nH]1	N	A
STOCK1N-00576	CC(C(N[C@@H](CCC(O)=O)C(O)=O)=O)Oc(cc1)cc(O2)c1C(c1ccccc1)=CC2=O	A	A
STOCK1N-00577	CCC[C@@H](CCC(O)=O)N	N	A
STOCK1N-00578	CC([C@@H]1Oc(c2ccc3)c3OC)[C@@H]2Oc2c1cccc2	N	N
STOCK1N-00580	CCCCCCCCCCCCCCCC(Nc1c(CCC)c(CCCC)nc2ccccc12)=O	A	A
STOCK1N-00583	CCCc(c(OC(C)C)c1)cc2c1OC(CCC)=C(c(cc1)cc3c1OCO3)C2=O	A	A
STOCK1N-00585	CC(Oc(cc1OC(C)=O)c(CN(CCC2)[C@@H]2C(O)=O)c(OC=C2Oc(cc3)ccc3F)c1C2=O)=O	A	A
STOCK1N-00586	CCOC(C(C)Oc1cc(OC(C=C2C)=O)c2c(OC(C)C(OCC)=O)c1)=O	N	A
STOCK1N-00590	OC[C@@H](C(O)=O)NC(C(NCC(O)=O)=O)=O	N	A
STOCK1N-00591	CC(C)[C@@H](C(OC)=O)NC(C(CSC1(C)C)N1C=O)=O	A	A
STOCK1N-00593	CC(CCc(c(C)nc(c1c2)ccc2OC)c1O)=O	N	A
STOCK1N-00597	CC(OC[C@@H]([C@@H]([C@H]([C@@H]1OC(C)=O)OC(C)=O)OC(C)=O)OC1Oc(ccc(C(NCCCN1CCCCC1)=O)c1)c1OC)=O.Cl	A	A
STOCK1N-00598	CC(CCCCCCCCCCCCCO)(C(O)=O)C(O)=O	N	A
STOCK1N-00600	O=C(CCc1c[nH]c2c1cccc2)c1ccccc1	N	A
STOCK1N-00601	COC(C([C@@H](C(C1O)O)O)O)[C@H]1O	N	A
STOCK1N-00607	C(CC1)CCC1(CC1)Cc2c1[nH]c1c2cccc1	N	A
STOCK1N-00614	CCCOC(C1(CCC(C)(C)C2)C2C2=CCC([C@](C)(CC3O)C(CC4)C(C)(C)C3O)[C@@]4(C)[C@]2(C)CC1)=O	N	A
STOCK1N-00618	CCC(NC(CC(C)(C)C1)=C([C@H]2c(c	N	A

	cc(OC)c3)c3OC)C1=O)=C2C(OCC)=O		
STOCK1N-00620	C[C@]1(CC2)C(C(c3ccccc3)=O)=CCC1C1C2[C@](C)(CCC(C2)OC(C)=O)C2=CC1	A	A
STOCK1N-00622	CC(C)COc(cc1)ccc1C(N/C(/C(NCCCO)=O)=C\c(cc1)cc2c1OCO2)=O	A	A
STOCK1N-00624	CC(n1c(cccc2)c2c(C(C(Oc2c3cccc2)=O)=C3O)c1)=O	N	A
STOCK1N-00625	CC(C(C1C(N2)=O)C2=O)C(C)=CC1C=C(C)C	N	A
STOCK1N-00626	CCc(c(OCC(NCCCC(O)=O)=O)c1)cc(C(C)=C2)c1OC2=O	A	A
STOCK1N-00629	C(CN1CSCC1)c1c[nH]c2c1cccc2.Cl	N	A
STOCK1N-00630	CCOC(CN(CCCC1)[C@@H]1c1cnccc1)=O	N	A
STOCK1N-00632	CC(c(ccc(OCC=C)c1C)c1O1)=CC1=O	N	A
STOCK1N-00636	CCCCCC(CC1CC(O)=O)OC1=O	N	N
STOCK1N-00637	C[C@@H]1C2(CO)C(C)C=C(C)C1C(c1ccco1)OC2	N	A
STOCK1N-00641	CC(c1c(/C=C2\N=C(/C=C(/C(C)/C3CCC(OC)=O)\N/C3=C\C(C(CCC(OC)=O)/C3C)=N/C3=C3)C(C(C)OC)=C2C)[nH]c3c1C)OC	N	A
STOCK1N-00642	CC1(C)c(cccc2)c2N(C2)[C@@]1(/C=C/c1cccs1)NC2=O	A	N
STOCK1N-00644	CC(C)(C1)OCC[C@@H]1C(O)=O	N	A
STOCK1N-00645	CC(C)C[C@@H](C(O)=O)NCc(c(O1)c(cc2)-c(cccc3)c3C1=O)c2O	N	A
STOCK1N-00651	CC(Oc(cc1OC(C)=O)cc(OC(C)=C2c3csc(C)n3)c1C2=O)=O	A	A
STOCK1N-00653	CCCCCc(cc1)ccc1C(/C=C1/c(cc(cc2)OC)c2OC(c2ccc(CCCCC)cc2)=C1)=O	A	A
STOCK1N-00657	OC(CCCC1OC(CCCC(O)=O)OC(CCCC(O)=O)O1)=O	N	A
STOCK1N-00658	CC(C(C(C1)/O2)[C@](C)(CC3)C1C1C3[C@](C)(CCC(C3)OC(C)=O)C3=CC1)/C2=C(\C)/CC(CNC(C)=O)I	A	A
STOCK1N-00660	C[C@@H](CN(CCc1ccccc1)S([O-])(=O)=O)O.[K+]	A	A
STOCK1N-00661	CC(C)(Cc1nc2ccccc2c(NCc2c(C)cc(C)cc2)c11)CC1=O.OC(C(O)=O)=O	A	A

STOCK1N-00662	CC(C)(Cc1c2ccc3ccccc13)N=C2NCC(O)=O	N	A
STOCK1N-00664	C[C@@H](C(O)=O)NCc(c(OC=C1Oc(cc2)ccc2F)c(cc2)C1=O)c2O	N	A
STOCK1N-00666	CCCCC(Nc1c(C)c(CC)nc2ccccc12)=O	N	A
STOCK1N-00667	CCN(c(cccc1)c1C(O)=C1C(N2)=Nc(cccc3)c3C2=O)C1=O	A	A
STOCK1N-00670	COc(ccc(CC=C)c1)c1O	N	A
STOCK1N-00673	CCC(C)[C@@H](C(OC)=O)NC(COc(cc1)cc(OC=C2Oc3ccccc3)c1C2=O)=O	A	A
STOCK1N-00674	CC(C)(CC(C1C(/C=C/c2ccco2)=O)=O)CC1=O	N	A
STOCK1N-00678	CCOC(C([C@]1(c(cccc2)c2N2)C2=O)=C(c2ccccc2)OC(N)=C1C(OCC)=O)=O	A	A
STOCK1N-00680	CCCCC[C@@H](C[N+](C)(C)C)C(/C=C/c1ccccc1)=O.[I-]	A	A
STOCK1N-00685	CC(C)(C)OC(N[C@H](CSCNC(c1ccccc1)=O)C(Oc(cc1)cc(OC=C2c(cc3)cc4c3OCCO4)c1C2=O)=O)=O	A	A
STOCK1N-00686	CC(OC[C@@H]([C@@H]([C@H]([C@@H]1OC(C)=O)OC(C)=O)OC(C)=O)O[C@@H]1Sc(c(O)c(c(C(C(OC)=C1C)=O)c2O)C1=O)c2S[C@@H]([C@@H]([C@H]1OC(C)=O)OC(C)=O)O[C@H](COC(C)=O)[C@H]1OC(C)=O)=O	A	A
STOCK1N-00687	COc(cc1)cc2c1[nH]cc2CCNC(c(cc1)ccc1Cl)=O	N	N
STOCK1N-00692	CC(c(ccc(OCC(Oc(cc1)cc(OC(C(F)(F)F)=C2Oc(cc3)ccc3C(OC)=O)c1C2=O)=O)c1)c1O1)=CC1=O	A	A
STOCK1N-00694	CC/N=C1\C(C(CC(C)(C)C2)=O)=C2Nc2c1cccc2.OC(C(O)=O)=O	N	A
STOCK1N-00700	CC(C)(C1)OCC[C@@H]1C(COc(cc1)cc(O2)c1C(c1ccccc1)=CC2=O)=O	A	A
STOCK1N-00706	CCCc(c(OCC)c1)cc(C2=C3CCC2)c1OC3=O	N	A
STOCK1N-00708	CC(C)([C@@H]1CC=C(C)[C@H]2C1)OC2c(cc1)ccc1O	N	A
STOCK1N-00709	CC(N/C(/C(N)=O)=C/c(cc1)cc2c1O	N	A

	CO2)=O		
STOCK1N-00710	CCNCC1(CCCC1)c(cc1)cc(OC)c1OC.Cl	N	A
STOCK1N-00711	CC(C)CCn1c(N(C)C)nc(N(C)C(N2)=O)c1C2=O	A	A
STOCK1N-00712	CC(C)C[C@@H](C(O)=O)NC(C(C)Oc(cc1)cc(O2)c1C(c1ccccc1)=CC2=O)=O	A	A
STOCK1N-00713	OC(CNC(CCCCCCCC(NCC(O)=O)=O)=O)=O	N	A
STOCK1N-00714	CC(CCC1(C)C(C)=CCCC11C)C1OC=O	N	A
STOCK1N-00716	CCc(c(OC)c1)cc2c1OC=C(c1ccc(C(OCC)=O)o1)C2=O	A	N
STOCK1N-00717	C[C@@H](C(NCC(CC1)CCC1C(O)=O)=O)Oc(cc1)cc(O2)c1C(CCC1)=C1C2=O	N	A
STOCK1N-00718	O=C(/C=C/c(cc1)cc2c1OCCO2)c(cccc1)c1OCc1ccccc1	A	N
STOCK1N-00719	COC([C@H](Cc(cc1)ccc1O)NC([C@H](CC1)N(C(OCc2ccccc2)=O)C1=O)=O)=O	A	A
STOCK1N-00720	CC(C)([C@@H](CC1)O[C@]1(C)[C@H]([C@@H](C1)OC(C)=O)[C@](C)(CC2)[C@@]1(C)[C@@H](C[C@H]1OC(C(C3OC(C)=O)OC(C)=O)OC(COC(C)=O)C3OC(C)=O)[C@@]2(C2)[C@]2(CC2)C1C(C)(C)[C@@H]2OC(C(C1OC(C)=O)OC(C)=O)OCC1OC(C)=O)O	A	A
STOCK1N-00722	Cc1ncc(CCn2c(cccc3)c3c3c2CCN(C)C3)cc1.Cc1ncc(CCn2c(cccc3)c3c3c2CCN(C)C3)cc1.OS(O)(=O)=O.OS(O)(=O)=O.OS(O)(=O)=O	A	A
STOCK1N-00725	CC1=CCC(CN(CCCN(C)C)CC2CC=C(C)CC2)CC1	N	A
STOCK1N-00726	CCOC(c1c(CCN(C(c2c3cccc2)=O)C3=O)c(cc(cc2)Br)c2[nH]1)=O	A	A
STOCK1N-00727	CC(C)C[C@H](C(NCC(N)=O)=O)NC([C@H](CCC1)N1C(C(CC1)CCN1C([C@H](CCC1)N1C(OCc1ccccc1)=O)=O)=O)=O	A	A
STOCK1N-00729	CC(C)C1OCC2(CO)N1C1(CCCCC1)O	N	A

	C2		
STOCK1N-00730	CC(Oc1c2ccc(OCC(ON(C(CC3)=O)C3=O)=O)c1)=C(c(cc1)cc3c1OCCCO3)C2=O	A	A
STOCK1N-00734	CCCc(c(OC)c1)cc2c1OC(C)=C(c(cc1)cc3c1OCCCO3)C2=O	N	A
STOCK1N-00735	CC(NC(N[C@H](CSC1)[C@H]1OC(C)=O)=O)=O	A	A
STOCK1N-00736	CC(C)(C(CC12)C3)[C@]11[C@@]23C(C)(C)CC[C@H]1O	N	A
STOCK1N-00737	CCOc(cc1)ccc1C(OCC[C@@H]1C(CCCC2)[N+]2(C)CCC1)=O.[I-]	A	A
STOCK1N-00740	CC(C)NC[C@@H](c(cc1)cc(O)c1O)O.Cl	N	A
STOCK1N-00742	CCCCCCCCCOC(O[C@H]1CC2=CCC(C3[C@@](C)(CC4)C(CCCCCCCC)CC3)C4[C@]2(C)CC1)=O	A	A
STOCK1N-00743	OC(COc(cc1OCC(O)=O)cc(O2)c1C(c1ccccc1)=CC2=O)=O	N	A
STOCK1N-00744	CC(C)(C1)CC(NC(C[C@H]2c(cc3)ccc3OC)=O)=C2C1=O	N	A
STOCK1N-00746	CC(C)(C1)CC(N(CC2)[C@@H](C/C3=N\OC)c4c2cccc4)/C3C1=O	A	A
STOCK1N-00751	CC(C)=C(C(NCCCC(O)=O)=O)NC(c1ccccc1)=O	N	A
STOCK1N-00753	O[C@@H](CNCC1(CCCC1)c(cc1)cc2c1OCCO2)COc1ccccc1.Cl	N	A
STOCK1N-00754	CC(C)C(N(CCCN(C)C)C1)OC1c1ccccc1	N	A
STOCK1N-00755	CCOc(cc1OCC)cc(O2)c1C(c1ccccc1)=CC2=O	N	A
STOCK1N-00757	COC(CCC/C=C(\C1=O)/SC[C@@H]1NC(c1ccccc1)=O)=O	N	N
STOCK1N-00761	CN[C@@H](CCc(c(C1=CC=C2NCCO)c3OC)cc(OC)c3OC)C1=CC2=O	N	A
STOCK1N-00762	CC(C)(C=C(C)c1c2)Nc1cc(CN(C)C)c2O	N	A
STOCK1N-00764	CC(C)[C@H](OC1)OC[C@@]11[C@@H](C)CC(C)=CC1	N	A
STOCK1N-00765	CC(C1)C(COC(NC2CCCCC2)=O)(COC23)[C@@H](C)C3C1(C)Oc1c2cccc1	N	A
STOCK1N-00766	COc(cc1)cc2c1[nH]cc2CCNC(C1CC	N	A

	1)=O		
STOCK1N-00769	O=C1Oc(ccc(Cl)c2)c2C=C1c(cc1)cc2c1OCO2	N	A
STOCK1N-00770	Cc1c(C(/C=C/c(cc2)cc(OC)c2OC)=O)c(-c2ccccc2)c(cc(cc2)Br)c2n1	A	A
STOCK1N-00771	CCCCCCCCC(CC1)[C@](C)(CC2)C1C1C2[C@](C)(CC[C@H](C2)OC(/C=C/c(cc3)cc(OC)c3OC)=O)C2=CC1	A	A
STOCK1N-00772	CCCCCc(c(O)c1)cc2c1OC=C(c1nc(C)cs1)C2=O	A	N
STOCK1N-00777	OC[C@@H]([C@@H]([C@@H]1O)O)O[C@@H]1n1c2ncnc(NC3CCCCC3)c2nc1	N	A
STOCK1N-00778	COc(cc1)cc(S(c2c(C(O)=O)c(OC)ccc2OC)(=O)=O)c1OC	N	N
STOCK1N-00782	C[C@](CC1)(C(CC2)C3C1[C@@](C)(CCCC1)C1CC3)[C@@]12OCC1	N	A
STOCK1N-00784	CC(C)(C)OC(NC1(CCCCC1)C(N[C@@H](CCSC)C(O)=O)=O)=O	N	A
STOCK1N-00787	CN(C)C[C@H](CC(C(c1ccccc1)=O)=C)C(c1ccccc1)=O.Cl	N	N
STOCK1N-00788	CC(c(ccc(O)c1)c1O1)=C(C)C1=O	N	A
STOCK1N-00791	CCOc(cc1)ccc1OC1=C(C)Oc(cc(cc2)OCC(OC3CC4=CCC(C(CC5)[C@@](C)(CC6)CC5C(C)CCCC(C)C)C6[C@]4(C)CC3)=O)c2C1=O	A	A
STOCK1N-00793	C[C@](CC1)(C(CC2)[C@H](CC3)C1[C@@](C)(CC1)[C@@H]3CC1=O)[C@]2(C(COC(C)=O)=O)O	N	A
STOCK1N-00794	CC(C)(C)OC(NCCC(Oc(cc1)cc(O2)c1C(C)=CC2=O)=O)=O	N	A
STOCK1N-00795	CCCC1OC[C@]2(COC(Nc3ccccc3)=O)C(C)C=C(C)C1[C@@H]2C	N	A
STOCK1N-00799	C[C@](CC1)(C(CC2)C(CC3)C1[C@@](C)(CC1)[C@@H]3C[C@@H]1N=[N+]=[N-])[C@@H]2O	A	A
STOCK1N-00803	C[C@@H]1C2(COC(C)=O)C(C)C=C(C)C1C(CN1CCCCC1)OC2	N	A
STOCK1N-00807	CC(OCC(C(C(C1OC(C)=O)OC(C)=O)OC(C)=O)OC1OC(C(c(c1ccc2)c2O)=O)=CC1=O)=O	A	A
STOCK1N-00810	CC(C)Oc1ccc(/C=C(\C(N[C@@H](CCSC)C(O)=O)=O)/NC(c2ccccc2)=O)	A	A

	cc1		
STOCK1N-00811	CCCCCCc(c(O)c1)cc(C=C2c3cn(cccc4)c4n3)c1OC2=O	A	N
STOCK1N-00815	COc(cccc1)c1C(OC[C@@H]1[C@H](CCCC2)N2CCC1)=O	N	A
STOCK1N-00816	C[C@](C1)(CN2C(c(cccc3OC)c3O)N1C[C@]1(C)C2)C1=O	A	A
STOCK1N-00817	CCCCCCc(c(O)c1)cc(C(C)=C2CC(OCC)=O)c1OC2=O	N	A
STOCK1N-00818	CCCCCCCCSCC(CN(C[C@@H](C1)CN23)C[C@H]1C3=CC=CC2=O)O	A	A
STOCK1N-00820	O=C(/C=C/c(cc1)cc2c1oc1c2cccc1)c1ccccc1	N	N
STOCK1N-00823	OC(C[C@@H](C(O)=O)NC(c1cnccc1)=O)=O	N	A
STOCK1N-00827	OCCCCCCCCCCCC(C(O)=O)C(O)=O	N	A
STOCK1N-00828	CC(C)C[C@@H](C(O)=O)Nc1ncnc2c1[nH]cn2	N	A
STOCK1N-00830	O=C(CCNC(OCc1ccccc1)=O)Oc(cc1)cc(OC=C2c(cc3)cc4c3OCCO4)c1C2=O	A	A
STOCK1N-00831	CCCC(CC(C)(c1csc(NCCNc2nc(C(C)(CC3CCC)OC3=O)cs2)n1)O1)C1=O.Br.Br	A	A
STOCK1N-00832	CCOC(COc(cc1O)cc(OC(c2ccccc2)=C2)c1C2=O)=O	N	A
STOCK1N-00833	CC(Cc1ccccc1)NC(CC(c1ccccc1)c(ccc(C)c1C)c1O)=O	A	A
STOCK1N-00838	CC(CCOC1)(C1NCC(O)=O)O	N	A
STOCK1N-00840	Oc(cccc1C(c2c3cccc2O)=O)c1C3=O	N	A
STOCK1N-00842	CC(C(C1)C(C)(C)C1/C1=C\c(cc2)ccc2Cl)/C1=O	N	N
STOCK1N-00843	CC(O[C@@H](CO[C@@H]([C@@H]1OC(C)=O)OC(C)=O)[C@@H]1OC(C)=O)=O	A	A
STOCK1N-00844	OC(c1cc(/C=C/C(c(cccc2)c2O)=O)c2OCOCc2c1)=O	N	A
STOCK1N-00846	CC[C@@H](C(OCC)=O)Oc(cc1)cc(O2)c1C(C)=C(CC)C2=O	N	A
STOCK1N-00847	CCc(c(OC)c1)cc2c1OC(C(OCC)=O)=C(c1csc(-c3ccccc3)n1)C2=O	A	A
STOCK1N-00849	COc1ccc(C(C(C(Oc2c3cccc2)=O)=C3O)C(C(Oc2c3cccc2)=O)=C3O)cc1	N	A

STOCK1N-00851	CCC[C@](CC1)([C@@](C)(CC2)C1C(CC1)C2[C@@](C)(C[C@H]2C)C1C[C@]12OC1)O	A	A
STOCK1N-00853	OC(CCCC1)(C1CC1(CC/C2=C\c(cc3)ccc3F)/C2=O)OC1c(cc1)ccc1F	A	A
STOCK1N-00861	CC(Oc(cc1OC(C)=O)cc(OC=C2Oc(cc3)ccc3Br)c1C2=O)=O	A	A
STOCK1N-00863	CCc(c(O)c1CN(C)C)cc2c1OC=C(c(cc1)cc3c1OCO3)C2=O	A	A
STOCK1N-00864	CCC(/N=C(\N)/Nc1nc2cc(OCC)ccc2c(C)n1)=O	A	A
STOCK1N-00865	CCCCCCCCC(CC1)[C@](C)(CC2)C1C1C2[C@](C)(CC[C@H](C2)SC(CC3CCCC3)=O)C2=CC1	N	A
STOCK1N-00868	COc(ccc(CC(c(ccc(O)c1)c1O)=O)c1)c1OC	N	A
STOCK1N-00869	CC(c(cc1OC)c(CC(OC)=O)cc1OC)=O	N	A
STOCK1N-00870	Cc(cc1)ccc1S(NCCCC(Oc(cc1)cc(OC(c2ccccc2)=C2)c1C2=O)=O)(=O)=O	A	A
STOCK1N-00872	CCOC(C1=Cc(ccc(OC(C)=O)c2)c2OC1=O)=O	N	A
STOCK1N-00874	CC(CCCCCCCCCCCCCCCO)(C(O)=O)C(O)=O	N	N
STOCK1N-00875	CC(C)(C1)OCC[C@@H]1C(CN(C)C)O.Cl	N	A
STOCK1N-00876	CN1c2nc3ccccc3c(N)c2CCCC1.OC(c1cc(Br)cnc1)=O	A	N
STOCK1N-00878	O=C1Oc(cc(cc2)OCc3ccccc3)c2-c2c1cccc2	N	A
STOCK1N-00880	CCCCCOP(C)(OC[C@@H]1[C@H](CCCC2)N2CCC1)=S.Cl	N	A
STOCK1N-00881	C[C@H](C(C(C1)O2)[C@](C)(CC3)C1C1C3[C@](C)(CCC(C3)O[C@H]([C@H]([C@@H]4OC(C)=O)O[C@H]([C@H]([C@@H]5OC(C)=O)OC(C)=O)O[C@@H](COC(C)=O)[C@@H]5OC(C)=O)O[C@@H](COC(C)=O)[C@@H]4O[C@H]([C@H]([C@H]4OC(C)=O)OC(C)=O)OC[C@H]4OC(C)=O)C3=CC1)[C@]12OCC(C)CC1	A	A
STOCK1N-00885	CC1(C)Nc(cc(cc2)OC)c2C(C)=C1	N	N
STOCK1N-00887	CC(C([C@]1(c(cc(cc2)Br)c2N2)C2=O)=C(C)OC(N)=C1C(OCCOC)=O)=O	A	A

STOCK1N-00890	CC1[C@@H]2C(C)=CC(C)[C@H]1COC2/C=C/C	N	N
STOCK1N-00891	CCc(c(O)c1)cc2c1OC(CC)=C(c(cc1)cc3c1OCO3)C2=O	A	A
STOCK1N-00892	CC(C)COC(N(c(cccc1)c1O1)C1=O)=O	N	A
STOCK1N-00894	OC(c(cc1)ccc1NC(c1cnccc1)=O)=O	N	A
STOCK1N-00897	Cc1c(CCNC(CCC(N(C[C@@H](C2)CN34)C[C@H]2C4=CC=CC3=O)=O)=O)c(cccc2)c2n1-c1ccccc1	A	A
STOCK1N-00898	CC([C@@H](C1)Cc2c1c(C)nn2C(c1ccccc1)=O)=C	N	A
STOCK1N-00902	CC(C)C[C@@H](C(N1C2CCC1)=O)NC2=O	N	A
STOCK1N-00903	CC(OCC(c1c(C)n(C)c(cc2)c1cc2OC)=O)=O	N	A
STOCK1N-00905	CCCCCC(C)(C1SC2)NCCN1C2=O.Cl	N	A
STOCK1N-00908	CN(CC12CCOCC2)Cc(cc2OC)c1cc2OC	N	A
STOCK1N-00910	COc(ccc(C(c1c(C[C@H]2C(N3CC=C)=O)c(cccc4)c4[nH]1)N2C3=S)c1)c1OC	A	A
STOCK1N-00911	CC(C)Oc1ccc([C@@H](CC(O)=O)C(O)=O)cc1	N	A
STOCK1N-00918	O=Cc(cc1)cc2c1N(Cc1ccccc1)CC2	N	N
STOCK1N-00919	CC(c1nc(cccc2)c2n1CC(Cn1c(cccc2)c2c2c1cccc2)O)O	A	A
STOCK1N-00920	CC(CC(CCC=C(C)C)=CC1)C1C1OC(CCl)CO1	N	N
STOCK1N-00921	CC(C)Oc1ccc(/C=C(\C(NCCCC(O)=O)=O)/NC(c2ccccc2)=O)cc1	A	A
STOCK1N-00923	CS(CCC(C(O)=O)N)=O	N	A
STOCK1N-00926	C[C@@H](Cc1c[nH]c2c1cccc2)NC(NC(c(cc1)ccc1OC)=O)=O	A	N
STOCK1N-00928	CC(C)(C1C/C2=O)C1/C2=C(\C)/NC(c1ccccc1)=O	A	A
STOCK1N-00929	CCCCCCCCCCCC(C(C([C@@H](C(C)(C)C1)C(OC)=O)=O)=C1OC)=O	A	A
STOCK1N-00932	CN(CC1)Cc2c1n(Cc1ccccc1)c1c2cccc1.OS(c1ccc(cc(cc2)S(O)(=O)=O)c2c1)(=O)=O	A	A
STOCK1N-00934	CC(C)(CCC1)C(CC2C(OCCN(C)C)=O)=C1CC2C(O)=O	N	A

STOCK1N-00943	OC(COc(cc1)cc(O2)c1C=CC2=O)=O	N	A
STOCK1N-00944	OC([C@@H](C1)[C@@H](Cc2cccc c2)CCC1=O)=O	N	A
STOCK1N-00945	CC(C)CCCC(C)C(CC1)[C@](C)(CC2)C 1C(CC1)C2[C@@]2(C)C1NCC2.Cl	N	A
STOCK1N-00946	COc(cc1)cc(C(/C=C/c(cc2)cc3c2OC CCO3)=O)c1O	N	A
STOCK1N-00947	Cc(cc1OC)cc(O2)c1C(c1ccccc1)=CC 2=O	N	N
STOCK1N-00949	COc(ccc(C(O[C@@H]1C[C@H](CC 2)N[C@H]2C1)=O)c1)c1OC	N	A
STOCK1N-00951	CC(C)C(C)(C)C(CC(C)CC1)C1=O	N	A
STOCK1N-00952	C[C@@H](C(c(cc1OC)c(CC2)cc1OC)N2C(CCC1)/C2C1=O)/C2=N\NC(N) =O.Cl	A	A
STOCK1N-00956	CCc(c(OC)c1)cc(C(Cc(cc2)cc3c2OC O3)=O)c1O	N	A
STOCK1N-00958	Cc1nc(C2=COc(c3c(cc4)OCCO3)c4 C2=O)cs1	A	A
STOCK1N-00960	[O-][N+](c(cc1)ccc1C(OCC([C@@H](C(C1O)O)O)O[C@@H]1O[C@](CO)(C 1O)O[C@H](CO)C1O)=O)=O	A	A
STOCK1N-00961	CC(C)(Cc1nc2ccccc22)C=Cc1c2N.Cl	N	A
STOCK1N-00962	O=C/C=C/C1=COc(cc(cc2)Br)c2C1= O	N	N
STOCK1N-00963	CCCCCCC(c1c(C2)c(cccc3)c3[nH]1) NC2C=O	N	A
STOCK1N-00967	CC(C12)([C@@H]2C2OC1C=C2)C(OCC=C)=O	N	A
STOCK1N-00968	C[C@@H](CCc1c- 2c3cc(Br)cc(Br)c3o1)c1c2oc(C)c1	N	A
STOCK1N-00969	CCCC(OC(N)=C([C@@]1(c(cccc2)c 2N2)C2=O)C(OCC)=O)=C1C(OCC)= O	N	A
STOCK1N-00970	CC(C)N(CC[C@@]1(Cc2ccccc2)CC(C)(C)OCC1)Cc1ccccc1.Cl	A	A
STOCK1N-00973	Nc1c2nc[nH]c2nc(CCO)n1	N	A
STOCK1N-00980	CC(C)C[C@@H](C(N(C)C(C)Cc(cc1) cc2c1OCO2)=O)NC(OC(C)(C)C)=O	A	A
STOCK1N-00981	CC(C)(CCC1)[C@@H](CC2)[C@]1(C)[C@H](CC/C(/C)=C\CO)[C@@]2(C)O	N	N

STOCK1N-00984	CN(C(C(Cc(cccc1OC)c1OC)(CN(C[C@@H](C1)CN23)C[C@H]1C3=CC=CC2=O)C(N1C)=O)=O)C1=O	A	A
STOCK1N-00985	CCN(c(cc1)c(c2ccc3)c3c1NC(c1ccco1)=O)C2=O	A	A
STOCK1N-00988	Cc(cc1)cc(OC(c(cc2)cc3c2OCCO3)=C2)c1C2=O	N	A
STOCK1N-00989	CC(Oc(cc1)cc(C(C)=C2)c1OC2=O)=O	N	A
STOCK1N-00990	COC(c(cc1)ccc1OC1=C(C(F)(F)F)Oc(cc(cc2)OC([C@H](CCC(OCc3ccccc3)=O)NC(OCc3ccccc3)=O)=O)c2C1=O)=O	A	A
STOCK1N-00991	CC(C)(C)OC(N[C@@H](CCSC)C(Oc(cc1)cc(OC(C)=C2c(cc3)cc4c3OCCO4)c1C2=O)=O)=O	A	A
STOCK1N-00997	O=C1N(CCC2)C2=Nc2c1cccc2	N	A
STOCK1N-00999	OC[C@H]([C@H]([C@H]1O)O)O[C@H]1SC(C(N1)=O)=NNC1=O	A	A
STOCK1N-01003	CCCCC(Nc1c(CCC)c(CCCC)nc2ccccc12)=O	N	N
STOCK1N-01004	C[C@@H](C(NCC(O)=O)=O)Oc(cc1)cc(O2)c1C(c1ccccc1)=CC2=O	A	A
STOCK1N-01005	O=C(c(cccc1)c1C1=Nc2c3cccc2)N1C3=O	A	A
STOCK1N-01006	CC(C)(C)OC(NCC(Oc(cc1)cc(O2)c1-c(cccc1)c1C2=O)=O)=O	N	A
